# Acute diacylglycerol production activates critical membrane-shaping proteins leading to mitochondrial tubulation and fission

**DOI:** 10.1038/s41467-025-57439-9

**Published:** 2025-03-19

**Authors:** Joshua G. Pemberton, Krishnendu Roy, Yeun Ju Kim, Tara D. Fischer, Vijay Joshi, Elizabeth Ferrer, Richard J. Youle, Thomas J. Pucadyil, Tamas Balla

**Affiliations:** 1 Section on Molecular Signal Transduction, Eunice Kennedy Shriver National Institute of Child Health and Human Development, National Institutes of Health, Bethesda, MD USA; 2 Biochemistry Section, Surgical Neurology Branch, National Institute of Neurological Disorders and Stroke, National Institutes of Health, Bethesda, MD USA; 3 Department of Biology, Western University, London, ON Canada; 4 Division of Development & Genetics, Children’s Health Research Institute, London Health Sciences Centre Research Institute, London, ON Canada; 5 Indian Institute of Science Education and Research, Pune, Maharashtra India

**Keywords:** Cell biology, Membrane structure and assembly, Mitochondria, Membrane curvature, Membrane fission

## Abstract

Mitochondrial dynamics are orchestrated by protein assemblies that directly remodel membrane structure, however the influence of specific lipids on these processes remains poorly understood. Here, using an inducible heterodimerization system to selectively modulate the lipid composition of the outer mitochondrial membrane (OMM), we show that local production of diacylglycerol (DAG) directly leads to transient tubulation and rapid fragmentation of the mitochondrial network, which are mediated by isoforms of endophilin B (EndoB) and dynamin-related protein 1 (Drp1), respectively. Reconstitution experiments on cardiolipin-containing membrane templates mimicking the planar and constricted OMM topologies reveal that DAG facilitates the membrane binding and remodeling activities of both EndoB and Drp1, thereby independently potentiating membrane tubulation and fission events. EndoB and Drp1 do not directly interact with each other, suggesting that DAG production activates multiple pathways for membrane remodeling in parallel. Together, our data emphasizes the importance of OMM lipid composition in regulating mitochondrial dynamics.

## Abstract (web summary)

Using complementary live cell approaches together with a powerful in vitro reconstitution system, this study demonstrates the direct regulation of mitochondrial dynamics by localized changes to the lipid composition of the outer membrane.

## Introduction

Mitochondria exist as dynamic networks that constantly undergo coordinated cycles of fusion and fission, which are critical for balancing essential cellular functions related to metabolism, signaling, and organelle quality control^1,2^. Foundational studies from diverse eukaryotic models, including recent insights from human pathologies^3,4^, have collectively characterized essential protein effectors that serve as determinants of the mitochondrial network architecture^5^. In particular, the process of mitochondrial fission is executed by a conserved dynamin-like GTPase, dynamin-related protein 1 (Drp1)^6,7^, which functions in concert with the outer mitochondrial membrane (OMM)-localized adapters, intricate inter-organelle contacts, and cytoskeletal interactions to orchestrate mitochondrial division^8^. Mitochondrial membrane fusion events are similarly controlled by specific subfamilies of dynamin-like GTPases^9^, with two structurally related transmembrane mitofusins (Mfn1/2)^10^ functioning to facilitate OMM fusion and differentially processed variants of optic atrophy 1 (OPA1)^11,12^ coordinating the fusion of inner mitochondrial membranes (IMM).

While much is known about the control of protein assemblies that regulate mitochondrial dynamics, the influence of specific membrane lipids on the mitochondrial fusion and fission processes remains poorly understood. These shortcomings are partly due to the significant difficulties associated with directly investigating spatially restricted changes in membrane lipid composition within intact cells. Furthermore, relative to other organelles, mitochondria are uniquely comprised of two membrane bilayers and possess a characteristic lipid composition that is defined by the selective enrichment of distinctive glycerophospholipid species, namely phosphatidylglycerol (PG) and cardiolipin (CL)^13–16^. Importantly, CL is synthesized from PG as part of a multi-step enzymatic cascade that occurs within the matrix-facing inner leaflet of the IMM^15^. As a result of this compartmentalized production, CL accounts for roughly 15–20% of the total phospholipid content of the IMM, while only representing ~ 1–10% of the steady-state lipid composition of the OMM^17–20^. Interestingly, CL has been shown to be involved in multiple functional aspects of mitochondrial biology^15^, including impacting network morphology. Specifically, high CL concentrations ( ~ 10–25% total lipids) have been shown to promote Drp1 assembly and stimulate its GTPase activity, in vitro^21–26^, and externalization of CL to the outer leaflet of the OMM^27–31^ has been proposed to function as a mediator of stress-induced mitochondrial fission during apoptosis and mitophagy^32–40^. However, it remains unclear how localized increases in the CL content within the OMM are controlled and whether, other than during stress-induced fragmentation, these mechanisms contribute to homeostatic mitochondrial fission under physiological conditions.

In addition to PG and CL, mitochondrial membranes also contain other common classes of glycerophospholipids, including phosphatidylcholine (PC; ~ 45%), phosphatidylethanolamine (PE; ~35%), phosphatidylinositol (PI; ~ 5–10%), and phosphatidylserine (PS; ~ 1–3%)^13,14,19,20^. Of these membrane lipid constituents, PE and CL are important for the maintenance of cristae ultrastructure and respiratory chain functions^15,16,41–43^. Alternatively, regulatory glycerolipids like phosphatidic acid (PA)^44,45^ and diacylglycerol (DAG)^46,47^ are comparatively low-abundance at the steady-state (~ 0.5–1.5% total lipids)^17,18,20^ but serve as essential metabolic intermediates within mitochondrial membranes and have been implicated in the complex regulation of mitochondrial morphology^48–50^. Specifically, accumulation of PA within the OMM has been suggested to function downstream of Mfn1/2-dependent membrane tethering to enhance mitochondrial fusion rates^51–53^ as well as negatively regulate Drp1-dependent mitochondrial division^54,55^. Conversely, prolonged overexpression of Lipin1β, which is a ubiquitous splice variant of the highly conserved Lipin/Pah family of lipid phosphatases that selectively convert PA to DAG^56–58^, induces pronounced fragmentation of the mitochondrial network in a manner that is dependent on both its enzymatic activity as well as Drp1^52,54^. Transient knock-down of Lipin1 similarly correlates with a significant elongation of the mitochondria^52^ and Lipin1-dependent production of DAG within the OMM has also been implicated in the regulation of oxidative stress-related signaling^59^ and may function as part of the membrane recognition signals used for autophagosome assembly during stress-induced mitophagy^60^. Taken together, these studies suggest that the relative balance of PA and DAG within the OMM serves as a membrane-intrinsic signal that can significantly impact mitochondrial network dynamics, potentially through direct regulation of Drp1-dependent fission. However, all prior studies rely on long-term over-expression or knockdown of the lipid-modifying enzymes, which are likely to broadly impact cellular lipid metabolism thereby making it difficult to assess whether these reported effects are a direct manifestation of changes in the relative lipid composition of the OMM or arise from secondary metabolic changes. Thus, a direct role for specific classes of phospholipids in the control of mitochondrial dynamics has not been firmly established experimentally.

To evaluate the direct effects of OMM lipid composition on mitochondrial dynamics, and increased levels of DAG in particular, we used induced-proximity of engineered lipid-modifying enzymes to selectively convert PI to DAG in the cytosolic leaflet of the OMM. Our results demonstrate that localized production of DAG initiates rapid and uniform fragmentation of the mitochondrial network in a Drp1-dependent manner. Furthermore, DAG production also recruits the Bin/Amphiphysin/Rvs (BAR) domain-containing proteins endophilin B1 and B2 (EndoB1/2) to the mitochondrial surface, which induces acute OMM deformation. Reconstitution experiments reveal that the presence of DAG directly facilitates the membrane binding and self-assembly of Drp1 as well as both isoforms of EndoB, which ultimately function to promote distinct membrane-shaping processes. However, EndoB1/2 and Drp1 do not directly interact with each other, suggesting localized DAG production activates multiple parallel pathways for membrane remodeling. Taken together, our results reveal that OMM lipid composition directly affects mitochondrial morphology, including direct activation of Drp1-dependent membrane fission events.

## Results

### Acute production of DAG within the OMM induces fragmentation of the mitochondrial network

**Fig. 1:**
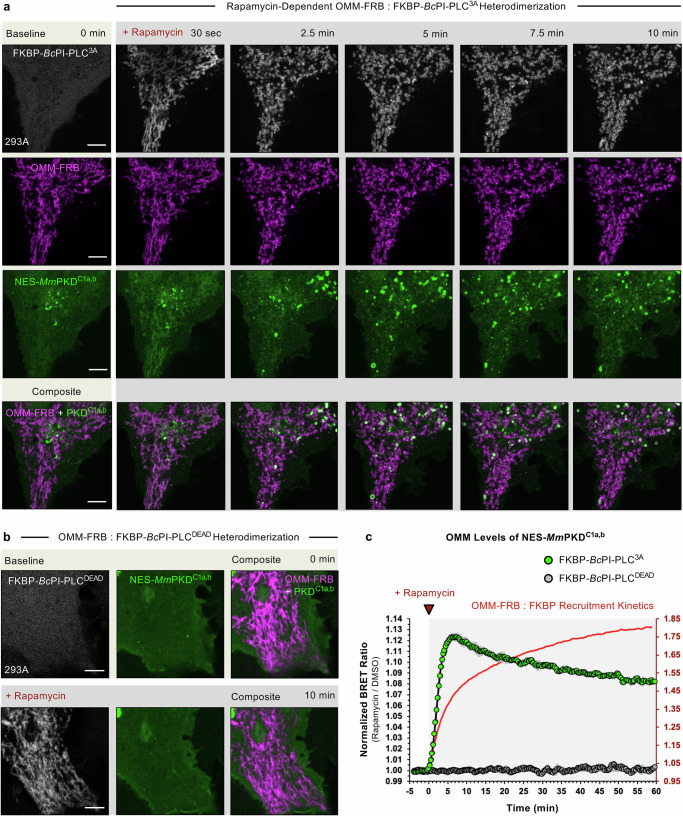
Recruitment of FKBP-*Bc*PI-PLC^3A^ to the OMM induces localized DAG production and rapid fragmentation of the mitochondrial network. **a**, **b** Representative images of HEK293A cells (5 μm scale bar) showing localization of the OMM-targeted FRB recruiter (OMM-FRB-ECFP, magenta) and a high-affinity DAG-binding probe (NES-mEGFP-*Mm*PKD^C1a,b^, green) in response to rapamycin-induced (100 nM) recruitment of either the catalytically active (**a**, mRFP-FKBP-*Bc*PI-PLC^3A^) or inactive (**b**, mRFP-FKBP-*Bc*PI-PLC^DEAD^) enzyme variants (gray) to the cytosolic membrane leaflet of the mitochondria. The composite images present an overlay of the OMM-FRB together with the DAG biosensor. **c** Kinetics of DAG production within the OMM of HEK293A cells following recruitment of iRFP-FKBP-*Bc*PI-PLC^3A^ (green trace) or iRFP-FKBP-*Bc*PI-PLC^DEAD^ (gray trace) to the cytosolic membrane leaflet of the mitochondria, as measured using the OMM-DAG^BRET^ biosensor (AKAP^TM^-mVenus-T2A-sLuc-NES-*Mm*PKD^C1a,b^). Population-level measurements in HEK293A defining the OMM-FRB:FKBP dimerization kinetics (red trace) for the FKBP-*Bc*PI-PLC^DEAD^ scaffold upon rapamycin-induced (100 nM) recruitment to the OMM, as measured using the OMM-FRB:FKBP^BRET^ biosensor (AKAP^TM^-FRB-mVenus-T2A-sLuc-FKBP-*Bc*PI-PLC^DEAD^; red trace). BRET measurements are presented as mean values ± SEM from three independent experiments carried out using triplicate wells.

Previously, we used iterative mutagenesis to generate a bacterial PI-specific phospholipase C (*Bacillus cereus* (*Bc*)PI-PLC) with minimal interfacial membrane binding (W47A/W242A; *Bc*PI-PLC^AA^) and tuned catalytic activity (R163A; *Bc*PI-PLC^3A^) to create an induced proximity system capable of rapidly hydrolyzing membrane-embedded PI, and thereby generating the direct enzymatic product, DAG, at precise locations within the endomembrane compartments of live cells^61^. Targeting this engineered enzyme system to the OMM was achieved using a chemically-induced heterodimerization approach that involves the rapamycin-dependent interaction between a *Bc*PI-PLC^3A^ variant tagged with an FK506-binding protein module, FKBP12 (mRFP-FKBP-*Bc*PI-PLC^3A^), and the FKBP-rapamycin binding (FRB) domain of mTOR, which was localized to the OMM using the membrane-anchored segment of AKAP1 (residues 34–63; OMM-FRB-ECFP)^62,63^. As expected, upon transient co-expression of these constructs, the FKBP-*Bc*PI-PLC^3A^ shows a diffuse localization throughout the cytosol, while the OMM-FRB localizes to the mitochondria in HEK293A cells (Fig. 1a). Addition of rapamycin (100 nM) causes rapid translocation of the FKBP-*Bc*PI-PLC^3A^ to the OMM within 30–45 secs. DAG generation was monitored by expressing a high-affinity DAG sensor (NES-mEGFP-*Mm*PKD^C1a,b^; C1a and C1b domains of *Mus musculus* (*Mm)* protein kinase D (PKD), Residues 134–343)^64^, which also translocates to the OMM upon rapamycin-induced recruitment of FKBP-*Bc*PI-PLC^3A^ (Fig. 1a and Supplementary Fig. 1; for further validation of the DAG biosensor, see Supplementary Fig. 2a–d). Rapamycin-induced recruitment of a catalytically inactive H32A mutant of FKBP-*Bc*PI-PLC^AA^ (mRFP-FKBP-*Bc*PI-PLC^DEAD^)^61^ to the OMM did not alter the localization of the DAG sensor (Fig. 1b). We previously used a unique bioluminescence resonance energy transfer (BRET)-based biosensor, OMM-DAG^BRET^ (AKAP^TM^-mVenus-T2A-sLuc-NES-PKD^C1a,b^), to follow the FKBP-*Bc*PI-PLC^3A^-induced changes to membrane DAG levels specifically within the cytosolic leaflet of the OMM at the cell population-level^61^. As expected, kinetic studies in HEK293A cells reveal an acute increase in DAG levels within the OMM upon rapamycin-induced recruitment of FKBP-*Bc*PI-PLC^3A^, but not with the catalytically inactive FKBP-*Bc*PI-PLC^DEAD^ (Fig. 1c). Note that the ~ 12% increase in the OMM-DAG^BRET^ ratio corresponds to a roughly 10-fold increase in the signal intensity measured for the acute translocation of the DAG biosensor to the mitochondrial network in single-cell imaging studies (Supplementary Fig. 1b). Remarkably, live imaging of HEK293A cells after rapamycin treatment showed that rapid recruitment of FKBP-*Bc*PI-PLC^3A^ was not only associated with the rapid translocation of the DAG sensor to the OMM, but also led to global fragmentation of the mitochondrial network within minutes (Fig. 1a). The widespread mitochondrial fragmentation induced by FKBP-*Bc*PI-PLC^3A^ recruitment to the OMM was also observed in other commonly used mammalian cell lines, including the HeLa (Supplementary Fig. 2e) and COS-7 lineages (Supplementary Figs. 2f and 3 and Supplementary Movie 1). Importantly, rapamycin-induced recruitment of the inactive enzyme (FKBP-*Bc*PI-PLC^DEAD^) failed to cause fragmentation of mitochondria, suggesting the requirement for local DAG generation for inducing fission (Fig. 1b).

Interestingly, the mitochondrial fission associated with FKBP-*Bc*PI-PLC^3A^ recruitment was also accompanied by swelling of the mitochondrial matrix, which also occurs within minutes of inducing the localized conversion of PI to DAG (Supplementary Movie 1). These findings raise the possibility that specific hydrolysis of PI within the OMM could potentially alter the activity of integral-membrane channels or transporters to dynamically regulate mitochondrial permeability. Although live imaging of HEK293A cells stably loaded with low concentrations of the potentiometric dye, TMRM^65^, which selectively partitions into the mitochondrial matrix, did not show a significant change in mitochondrial membrane potential in response to FKBP-*Bc*PI-PLC^3A^ recruitment and remained sensitive to the mitochondrial uncoupler, FCCP^66^ (Supplementary Fig. 4). These data indicates that PI conversion to DAG does not acutely alter membrane integrity and that the observed fission is also not the result of a change in mitochondrial membrane potential. Similar results were found using a matrix-targeted Ca^2+^ sensor, CEPIA2-mt^67^, which revealed no significant changes to mitochondrial Ca^2+^ handling, including acute uptake of cytosolic Ca^2+^ after the *Bc*PI-PLC^3A^-induced OMM fission (Supplementary Fig. 5). These findings further support the conclusion that the uniform fragmentation of the mitochondrial network does not coincide with generic damage to the OMM. In addition to these drastic structural changes to the mitochondrial network, a subset of mitochondria become significantly enriched in DAG in the OMM FKBP-*Bc*PI-PLC^3A^ recruitment (Fig. 1a and Supplementary Figs. 1, 2b, e, f, and 3 and Supplementary Movies 1 and 2). We reasoned that these DAG-enriched subpopulations of mitochondria result from the non-uniform re-supply of PI from the ER to only a subset of mitochondria, which would selectively support continued *Bc*PI-PLC^3A^-mediated DAG generation across the fragmented mitochondrial network. Non-uniform DAG levels may also reflect differential rates of clearance of DAG from the cytosolic leaflet due to the combined effects of rapid metabolic conversion of DAG^46,47^, transbilayer movement of DAG between the OMM leaflets^68–74^, or even accumulation of DAG within the interleaflet space of the OMM^75–78^.

### Mitochondrial fission resulting from acute DAG production within the OMM is dependent on Drp1

**Fig. 2:**
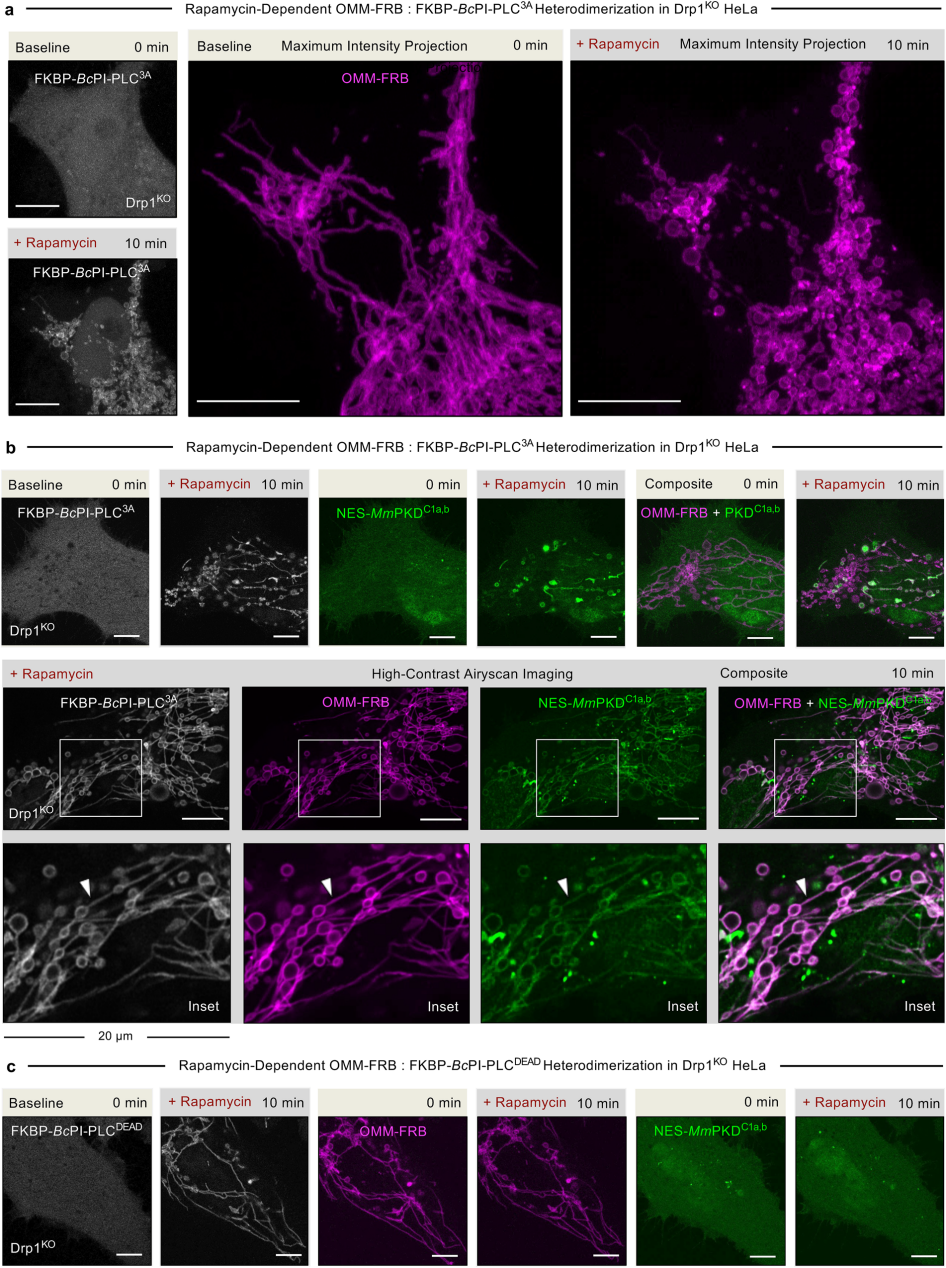
Mitochondrial fission induced by acute DAG production is dependent on Drp1. **a** Representative images of Drp1^KO^ HeLa cells (10 μm scale bar) showing the OMM-targeted FRB recruiter (OMM-FRB-ECFP, magenta) before (top panels) and 10 min after (bottom panels) rapamycin-induced (100 nM) recruitment of the catalytically active *Bc*PI-PLC^3A^ (mRFP-FKBP-*Bc*PI-PLC^3A^, gray) to the cytosolic membrane leaflet of the mitochondria. Note that to best show the overall change to the mitochondrial network morphology, these images show a maximum intensity projection that is generated from confocal slices along the *Z*-axis (4.830 μm total depth). **b** Representative images of Drp1^KO^ HeLa cells (10 μm scale bar) showing localization of the OMM-targeted FRB recruiter (OMM-FRB-ECFP, magenta) and a high-affinity DAG-binding probe (NES-mEGFP-*Mm*PKD^C1a,b^, green) before (left) or 10 min after (right and bottom row panels) rapamycin-induced (100 nM) recruitment of the catalytically active *Bc*PI-PLC^3A^ (mRFP-FKBP-*Bc*PI-PLC^3A^, gray) to the cytosolic membrane leaflet of the mitochondria. The composite images (right) show an overlay of the OMM-FRB together with the DAG biosensor. Note that the white arrowheads in the inset images (bottom row; 20 μm width) highlight hyper-constrictions of the OMM. The complete series of confocal sections associated with these images are provided as Supplementary Movie 3. **c** Representative images of Drp1^KO^ HeLa cells (5 μm scale bar) showing localization of the OMM-targeted FRB recruiter (OMM-FRB-ECFP, magenta) and a high-affinity DAG-binding probe (NES-mEGFP-*Mm*PKD^C1a,b^, green) before (left) or 10 min after (right) rapamycin-induced (100 nM) recruitment of the catalytically inactive enzyme variant (mRFP-FKBP-*Bc*PI-PLC^DEAD^, gray) to the cytosolic membrane leaflet of the mitochondria.

Drp1 has been shown to be necessary and sufficient for mitochondrial fission in mammalian cells^79^. We examined whether the rapid fragmentation of the mitochondrial network in response to acute DAG production in the OMM was dependent on Drp1. To investigate this, we tested the effects of acute FKBP-*Bc*PI-PLC^3A^ recruitment in HeLa cells lacking Drp1^38^. As previously reported, Drp1 knock-out (^KO^) HeLa cells display a characteristic elongated mitochondrial network and the presence of enlarged perinuclear mitochondrial bulbs. Upon rapamycin treatment, mitochondria in Drp1^KO^ cells showed prominent constrictions of the OMM as well as swelling of the matrix, however, the network remains inter-connected, which indicates that Drp1 is necessary for fission of the mitochondrial network (Fig. 2a). Interestingly, while fission was clearly inhibited, high-contrast airyscan imaging showed that the recruitment of FKBP-*Bc*PI-PLC^3A^ to the OMM in Drp1^KO^ cells caused the formation of extended OMM constrictions (Fig. 2b, white arrowhead) with prominent swelling of the mitochondrial matrix (Supplementary Movie 3). Again, recruitment of the catalytically inactive FKBP-*Bc*PI-PLC^DEAD^ to the OMM failed to elicit any of these changes to mitochondrial morphology in the Drp1^KO^ cells (Fig. 2C). Over-expression of fluorescently tagged Drp1 in Drp1^KO^ cells not only restored baseline mitochondrial morphology, but also supported the *Bc*PI-PLC^3A^-induced mitochondrial fragmentation upon rapamycin treatment. Consistent with these findings, treating HEK293A cells with siRNA for Drp1 similarly inhibited the rapamycin-induced fragmentation of the mitochondrial network (Supplementary Figs. 6a, b). Alternatively, over-expression of the GTPase-deficient mutants Drp1^K38A^ or Drp1^T59A^ have been shown to display a dominant-negative effect on Drp1 functions, which is characterized by a hyperfused mitochondrial network as well as the appearance of enlarged perinuclear mitochondrial aggregates^6,80^, also inhibited bulk mitochondrial fission in response to acute FKBP-*Bc*PI-PLC^3A^ recruitment. (Supplementary Figs. 6c–e). Together, these findings indicate that the rapid fragmentation of mitochondria observed upon acute DAG generation requires Drp1 functions.

**Fig. 3:**
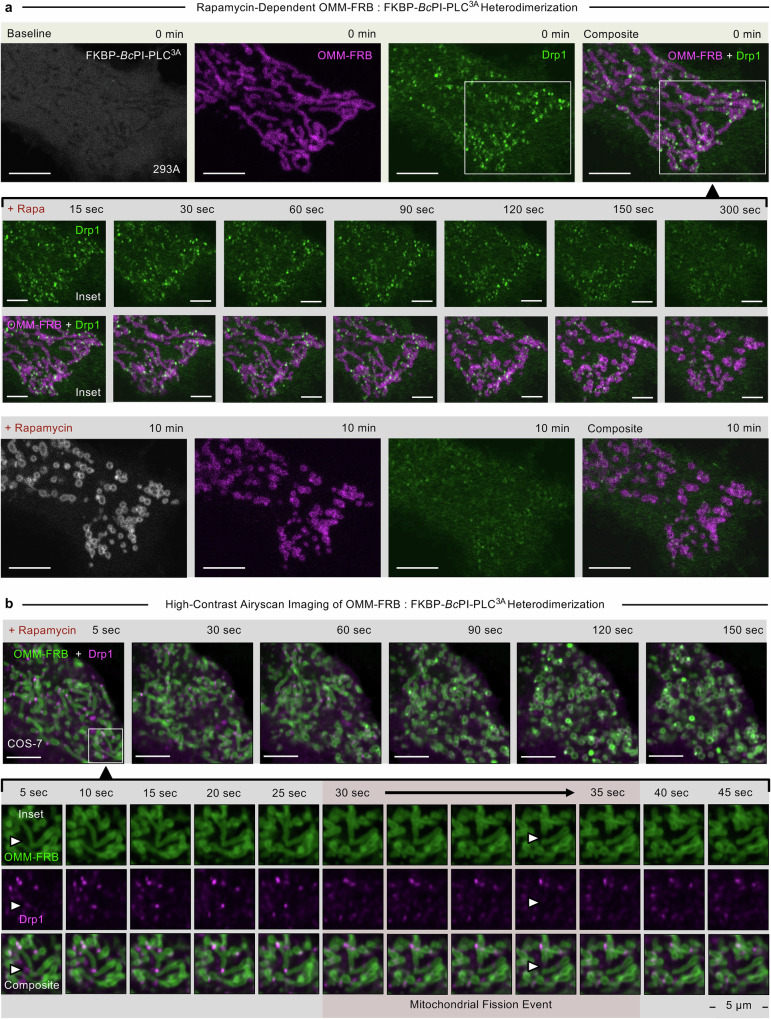
Induced mitochondrial fission by localized recruitment of FKBP-*Bc*PI-PLC^3A^ is associated with the rapid disassembly of OMM-associated Drp1 puncta. **a** Representative images of HEK293A cells (5 μm scale bar) showing the localization of the OMM-targeted FRB recruiter (OMM-FRB-mRFP, magenta) and Drp1 (mNG^HO^-Drp1, green) before (top row panels) or 10 min after (bottom row panels) rapamycin-induced (100 nM) recruitment of the catalytically active *Bc*PI-PLC^3A^ (emiRFP670-FKBP-*Bc*PI-PLC^3A^, gray) to the cytosolic membrane leaflet of the mitochondria. The early phase of the bulk fragmentation response of the mitochondrial network is shown over the initial 300 sec after rapamycin treatment in the inset panels (2.5 μm scale bar). **b** Representative composite images of COS-7 cells (5 μm scale bar) showing localization of the OMM-targeted FRB recruiter (OMM-FRB-ECFP, green) and Drp1 (mCherry-Drp1, magenta) in response to rapamycin-induced (100 nM) recruitment of the catalytically active FKBP-*Bc*PI-PLC^3A^ to the cytosolic membrane leaflet of the mitochondria. The bulk fragmentation response of the mitochondrial network is presented over the 150 sec period immediately after rapamycin treatment, while a detailed time series of the initial 45 sec post-rapamycin treatment (lower inset panels; 5 μm width); which is the time frame consistently associated with the majority of the OMM fission events, are presented separately as inset image panels. Image series presenting the individual image channels and overlays of the OMM-FRB together with either the NES-*Mm*PKD^C1a,b^ probe, or Drp1 are provided in Supplementary Movie 2.

Next, we wanted to understand the role of Drp1 in managing this process by expressing a fluorescently tagged Drp1 construct and monitoring its behavior in real-time. Live imaging of HEK293A and COS-7 cells expressing low levels of Drp1 showed a punctate localization along the OMM and enrichments in preformed assemblies, which typically localized to the middle or ends of mitochondria (Fig. 3a, b and Supplementary Fig. 3). Recruitment of FKBP-*Bc*PI-PLC^3A^ to the OMM caused some Drp1 puncta to initially become brighter, whereas, universally, completion of the associated mitochondrial fission resulted in a general decrease in the OMM-associated Drp1 puncta (Fig. 3a, b, Supplementary Fig. 3 and Supplementary Movies 2 and 4). We did not detect steady-state localization of the GTPase-deficient mutants of Drp1 (K38A or T59A) on the OMM after acute DAG production (Supplementary Fig. 6c–e). The ubiquitously expressed dynamin 2 (Dnm2)^81,82^ has also been proposed to be involved in the completion of mitochondrial fission^83^. We wanted to test if Dnm2 is involved in regulating the robust fission of the mitochondrial network upon acute DAG generation. As with the corresponding mutation to Drp1 (K38A), the GTPase-deficient K44A mutant of Dnm2 functions as a dominant negative in mammalian cells^84^. However, high levels of Dnm2^K44A^ over-expression did not significantly alter the mitochondrial network structure in HEK293A cells, nor did it have any effect on mitochondrial fragmentation upon DAG generation (Supplementary Fig. 7a). Rather, the Dnm2^K44A^ formed bright puncta throughout the plasma membrane (PM; Supplementary Fig. 7a), which is consistent with this mutant localizing to constricted pre-fission assemblies at endocytic sites^85^ and inhibiting the GTP hydrolysis cycle associated with endogenous dynamin isoforms^84^. In addition, unlike Drp1, over-expression of Dnm2 did not rescue the mitochondrial fission defect observed in the Drp1^KO^ cells, and Dnm2 also failed to associate with the remodeled OMM upon FKBP-*Bc*PI-PLC^3A^ recruitment in Drp1^KO^ cells (Supplementary Fig. 7b). We also did not detect localization of Dnm2 along the OMM at the steady-state, nor did we observe Dnm2 translocation to the mitochondria after FKBP-*Bc*PI-PLC^3A^ recruitment in HEK293A cells (Supplementary Fig. 7c). Overall, these data are in agreement with recent work showing that Drp1 is sufficient for mitochondrial fission, in vivo^79,86^, and support the conclusion that Drp1, and not Dnm2, is necessary and sufficient for the extensive mitochondrial fragmentation that is initiated upon recruitment of FKBP-*Bc*PI-PLC^3A^ to the OMM.

### DAG-dependent membrane remodeling assayed on supported membrane templates

To understand the molecular mechanism behind Drp1-dependent mitochondrial fragmentation upon the localized conversion of PI to DAG in the OMM, we tested the effects of DAG on Drp1 functions in vitro. For these studies, we utilized supported membrane templates (SMrTs)^87,88^, which are comprised of planar bilayers connected to an array of membrane tubes (Supplementary Fig. 8a). These templates, therefore, display a wide range of membrane topographies and curvatures, making them a suitable mimic of the complex topology of mitochondria. Furthermore, SMrTs have already been used previously to directly visualize Drp1-mediated membrane binding and GTP-induced fission^79^. Bulk measurements in mammalian cells show that PI constitutes ~ 5–10% of total mitochondrial phospholipids, with the OMM containing ~ 9–13% and the IMM containing ~ 2–5%^13,14,17–19^. In order to recreate the membrane lipid composition before and after the induced recruitment of FKBP-*Bc*PI-PLC^3A^ to the OMM, we prepared templates with moderate levels of 5% PI or 5% DAG together with 15% CL in a dioleoyl-phosphatidylcholine (DOPC) background. Templates also contained 1% of the fluorescent lipid TR-DHPE for visualization of the membrane. Importantly, stable templates with characteristic topographies could be readily formed with these specific lipid mixtures (Supplementary Fig. 8b–e). To directly visualize DAG distributions, we incubated templates with a recombinant low-affinity DAG sensor, mEGFP-*Mm*PKD^C1a^ (C1a domain (^C1a^) of *Mus musculus* (*Mm)* PKD, Residues 134–198)^89,90^, which selectively bound DAG-containing bilayers (Supplementary Fig. 8b, c) and curved membrane tubes (Supplementary Fig. 8d, e), but not to those prepared with PI. These data verify both the binding specificity of the DAG sensor as well as the accessibility of DAG to proteins on these prepared templates.

Owing to its negative spontaneous curvature^91–93^, DAG has been shown to induce packing defects in membranes at low concentrations and phase separate to cause localized membrane deformations at high concentrations^76,77,94–100^. To test the consequences of enzymatic conversion of PI to DAG, we incubated SMrTs containing 5% PI and 15% CL with purified recombinant *Bc*PI-PLC or the catalytically inactive *Bc*PI-PLC^H32A^ mutant for 30 mins. DAG production was confirmed by the selective enrichment of the DAG sensor on membrane tubes exposed to the active, but not the inactive *Bc*PI-PLC^H32A^ enzyme (Supplementary Fig. 8f, g). Importantly, membrane tubes retained their integrity after treatment with the *Bc*PI-PLC, and a paired image analysis of the same set of membrane tubes before and after the induced DAG production revealed a small, but significant, 1.5-fold increase in the tube radius (Supplementary Fig. 8h). This small increase in radius is still within the size range of membrane tubes where Drp1 shows high fission activity, as shown below. Thus, on membrane tubes, acute DAG production manifests in an expansion of the tube dimensions. It is important to highlight that exposure of the PI-containing templates to the recombinant *Bc*PI-PLC selectively generates DAG only in the outer leaflet of the tubule bilayer, which differs from the situation when the tubes are generated from the hydrated lipid mixture containing DAG. But the conversion of PI to DAG using the recombinant *Bc*PI-PLC was a relatively slow process and made it difficult to follow the changes to the lipid composition of the membrane templates in real time. We, therefore, resorted to using SMrTs prepared with fixed concentrations of PI or DAG, which effectively mimic the scenario before and after recruitment of FKBP-*Bc*PI-PLC3A to the OMM.

### DAG enhances Drp1-mediated fission of OMM-mimetic templates

**Fig. 4:**
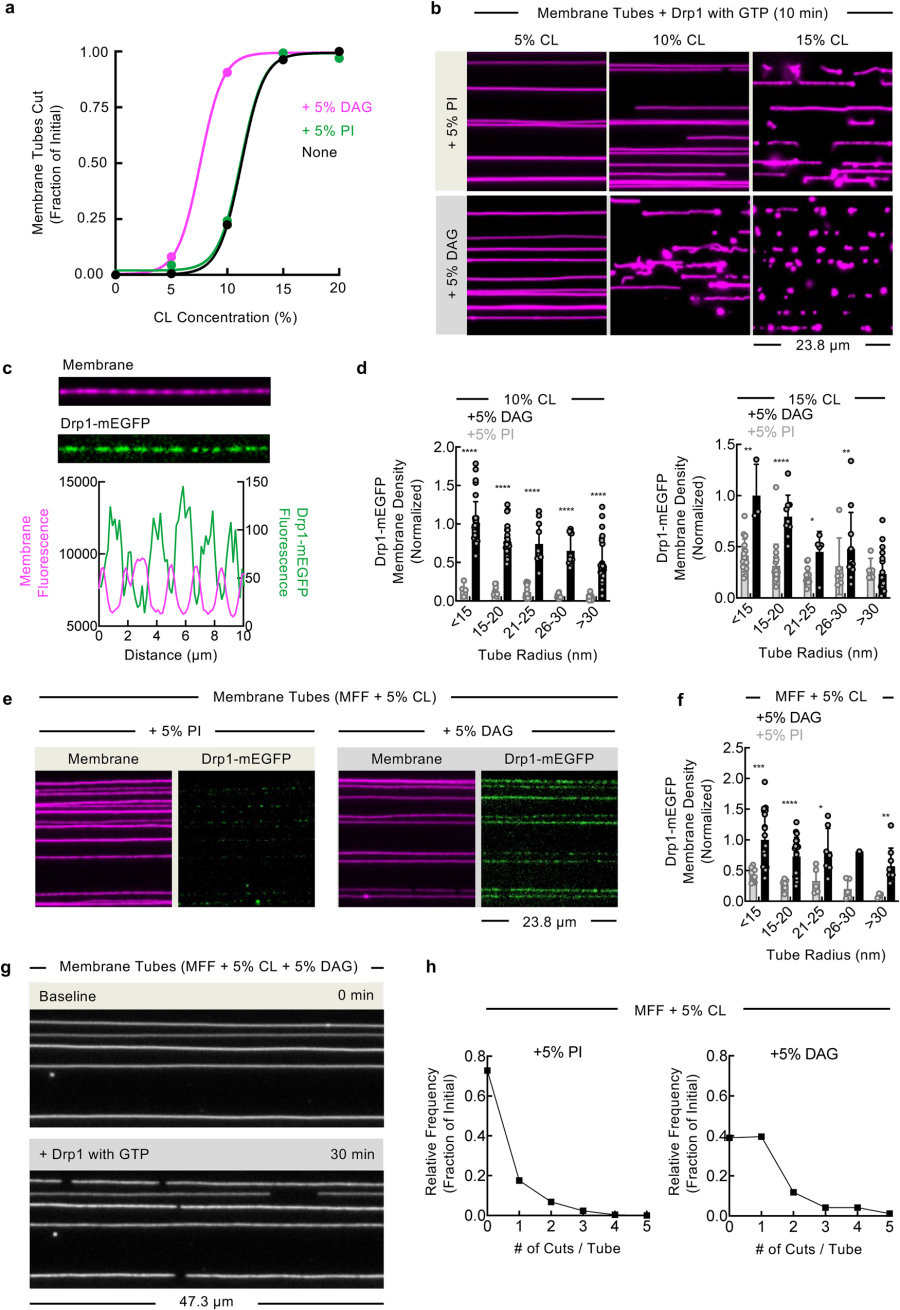
DAG enhances Drp1-mediated fission of OMM-mimetic templates. **a** Plots showing the fraction of membrane tubes with at least one cut in the presence of Drp1 and GTP. Data represents the analysis of at least 120 tubes for each of the indicated SMrT preparations. Membrane tubes contained either 5% DAG or 5% PI in a background of 5% CL (none). **b** Representative images of membrane tubes of the indicated lipid composition after 10 mins incubation with Drp1 and GTP. **c** Representative images (top) and fluorescence line profiles (bottom) of a membrane tube (magenta) with GppNHp-bound Drp1-mEGFP scaffolds (green). **d** The membrane density of Drp1-mEGFP scaffolds is reported as the ratio of the EGFP fluorescence and the membrane fluorescence on membrane tubes representing a range of sizes prepared using the indicated lipid composition. Data is normalized to the highest mean density for each condition and represents the mean $\pm \,$SD of at least 47 tubes. Statistical significance was estimated using unpaired Mann-Whitney’s test. **** denotes *p* < 0.0001, *** denotes *p* = 0.001, ** denotes *p* = 0.002, * denotes *p* = 0.02. **e** Representative images showing GppNHp-bound Drp1-mEGFP (green) on membrane tubes (magenta) displaying the 6xHis-tagged soluble domain of mitochondrial fission factor (MFF), which is recruited to the membrane by incorporating 5% of the DGS-NTA(Ni^2+^) lipid into templates. **f** The membrane density of Drp1-mEGFP is reported as the ratio of the EGFP fluorescence and the membrane fluorescence on membrane tubes representing a range of sizes prepared using the indicated lipid composition. Data is normalized to the highest mean density and represents the mean $\pm \,$SD of at least 30 tubes for each condition. Statistical significance was estimated using unpaired Mann-Whitney’s test. **** denotes *p* < 0.0001, *** denotes *p* = 0.001, ** denotes *p* = 0.009, * denotes *p* = 0.03. **g** Representative images of MFF- and CL-containing tubes (gray) before and after 30 mins incubation with Drp1 and GTP. **h** Quantification of the frequency of cuts per membrane tube for MFF-containing templates prepared with 5% PI or 5% DAG. Data represents the analysis of at least 169 tubes for each condition.

Our previous work using SMrTs to reconstitute a minimal Drp1-catalyzed membrane fission reaction revealed that the fission efficiency was highly dependent on the membrane CL concentration^79^. To assess the effects of DAG or PI on Drp1 functions, we prepared membrane templates with a range (0–20%) of CL concentrations either alone or containing moderate amounts of either 5% PI or 5% DAG and then flowed in Drp1 together with GTP. Bulk fission efficiency was analyzed by scoring the fraction of tubes that showed at least one cut after a 10 min incubation. Remarkably, the inclusion of 5% DAG caused a dramatic enhancement in Drp1-dependent fission efficiency at an intermediate concentration of 10% CL, where both the fraction of tubes cut and the number of cuts per tube showed a significant increase (Fig. 4a, b). The inclusion of 5% PI showed no effect on the fission efficiency across the entire CL concentration range (Fig. 4a, b). Collectively, these results emphasize that DAG facilitates the CL-dependent fission process catalyzed by Drp1. The bulk enhancement of the fission capacity measured for Drp1 led us to investigate whether DAG facilitates the binding of Drp1 to CL-containing membranes. Our previous work reported that mixtures of Drp1-mEGFP together with untagged Drp1 can efficiently scaffold, constrict, and cause fission of CL-containing membrane tubes in the presence of GTP^79^. Similar reactions carried out in the presence of a non-hydrolysable GTP analog, GppNHp, can be used to stall fission at a stage where Drp1 organizes as stable scaffolds that constrict the underlying tube, which is evident from the dimmer tube fluorescence under Drp1 foci (Fig. 4c). Under these conditions, measuring the ratio of the Drp1-mEGFP and membrane fluorescence intensities provides an estimate of the relative Drp1 density on the membrane. Notably, Drp1 preferentially bound membrane tubes compared to the planar bilayers, thereby indicating that Drp1 intrinsically prefers to bind membranes of high curvature (Supplementary Fig. 8i, j). Further analysis of membrane tubes revealed that the presence of DAG with intermediate (10%) and high (15%) CL concentrations recruited significantly more Drp1 across a wide range of tube sizes (Fig. 4d). Thus, these data support a role for DAG in enhancing Drp1-dependent fission by directly facilitating the binding of Drp1 to CL-containing membranes. Mitochondrial fission relies on the localized assembly of the intricate Drp1-associated divisome, which forms through coincident interactions with membrane-bound adapter proteins and lipids present on the surface of the OMM^8^. We tested if the facilitatory effects of DAG on Drp1-mediated fission also manifest in the presence of an adapter protein on the membrane. For this, we prepared membrane templates displaying the soluble domain of an important Drp1 adapter protein, mitochondrial fission factor (MFF)^101,102^, using previously described methods^79^. To decipher contributions from the MFF adapter on Drp1 functions, we lowered the membrane CL concentration to 5% and included either 5% PI or 5% DAG in the templates. Flowing Drp1-mEGFP with GppNHp onto these OMM-mimetic templates revealed significant differences depending on whether they contained DAG or PI. Compared to those prepared with PI, DAG-containing templates recruited significantly higher amounts of Drp1-mEGFP (Fig. 4e, f). On these templates, the Drp1-mEGFP signals appeared relatively non-uniform (Fig. 4e), which was unlike those observed on templates with high (15%) concentrations of CL (Fig. 4c). Notably, the addition of Drp1 and GTP showed no fission of membrane tubes prepared with 5% CL, regardless of whether they contained PI or DAG (Fig. 4a, b). However, the presence of MFF allowed Drp1 and GTP to cause fission at this relatively low level of 5% CL, even if the frequency of cuts per tube and the total fraction of tubes cut were significantly smaller than those observed at higher CL concentrations (Fig. 4g, h). In assays carried out with MFF-displaying templates with such low concentrations of CL (5%), the addition of Drp1 and GTP elicited at least one cut in 20% of the tubes for PI-containing templates, which was increased to 40% for DAG-containing templates (Fig. 4h). These data highlight the important role that adapter proteins play in facilitating Drp1-mediated fission. Importantly, even under these extremely stringent conditions, templates prepared with DAG displayed a higher frequency of cuts per tube than those prepared with PI (Fig. 4h). Together, these results show that DAG facilitates Drp1-catalyzed membrane fission on bare CL-containing membranes as well as on OMM-mimetic templates.

### DAG potentiates the mechanochemical activity of Drp1 to accelerate membrane fission

**Fig. 5:**
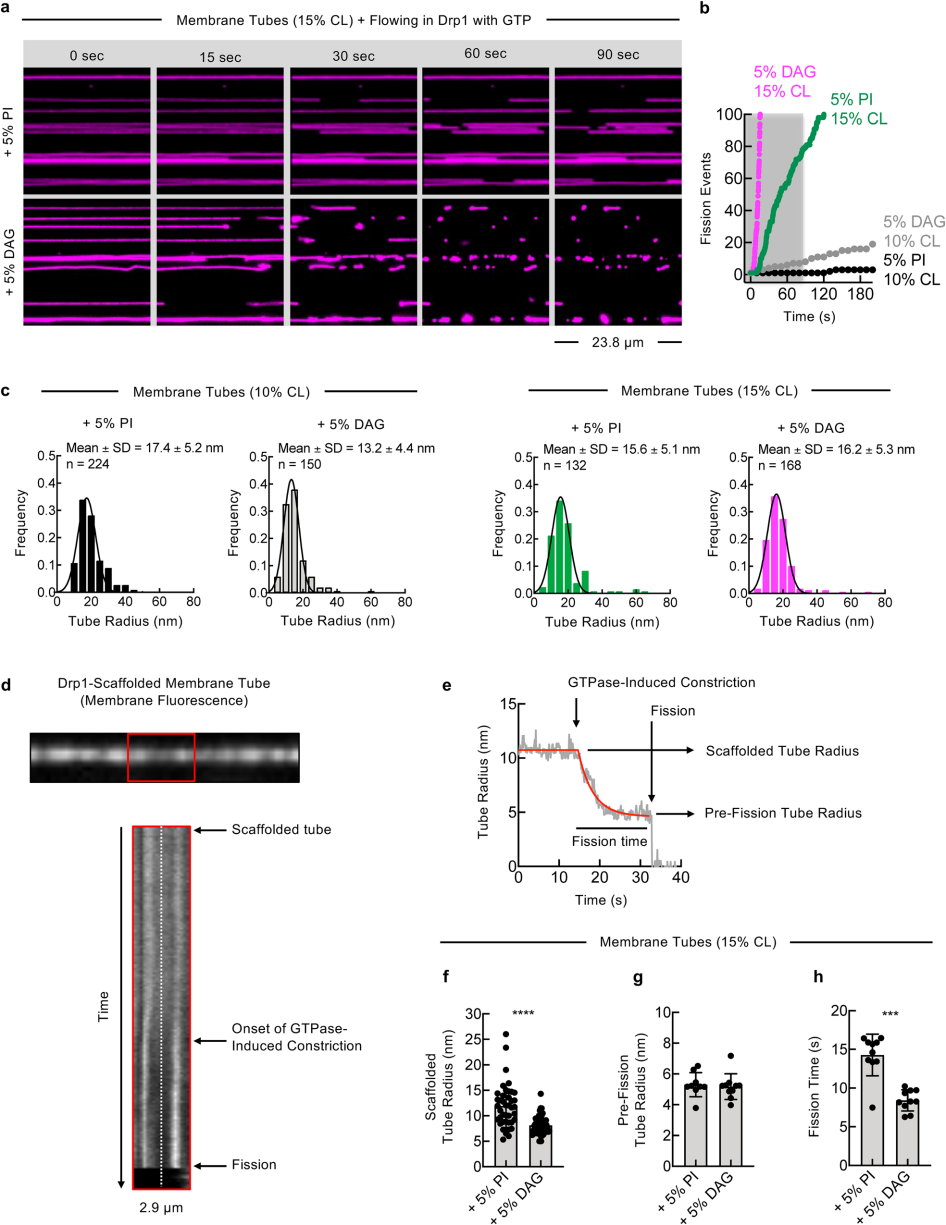
DAG potentiates the mechanochemical activity of Drp1 to accelerate membrane fission. **a** Representative images from a time series showing membrane tubes of the indicated lipid composition after flowing in Drp1 and GTP. The complete time series associated with these images are provided as Supplementary Movie 5. **b** Plots showing the kinetics of fission on tubes of the indicated lipid compositions upon flowing in Drp1 with GTP. **c** Membrane tube size distributions for templates formed of the indicated lipid compositions. Histograms were fitted to a Gaussian distribution to calculate the mean and SD. **d** Top horizontal panel shows a representative fluorescence micrograph of a membrane tube imaged for membrane fluorescence after Drp1 binding and before GTP addition. A time-lapse movie was then acquired upon flow through of GTP. The bottom vertical panel shows a kymograph generated across the central region of the membrane tube, which is boxed in red, from such a movie showing the stepwise constriction and fission of the membrane tube. **e** Plot showing the membrane fluorescence profile (gray) along the white dotted line shown in the kymograph in (**d**) after the conversion of this signal to the membrane tube radius using the previously described in situ calibration procedure^87^. The profile was fitted to a plateau with a one-phase exponential decay function (red) to estimate the scaffolded tube radius and the pre-fission tube radius. Fission time is measured as the interval between the onset of GTPase-induced constriction and membrane fission. **f** Quantification of the scaffolded tube radius on membrane templates of the indicated lipid composition. Data represents the mean$\,\pm \,$SD of at least 40 scaffolds. **g** Quantification of the pre-fission tube radius on membrane templates of the indicated lipid composition. Data represents the mean$\,\pm \,$SD of at least 9 fission events. **h** Quantification of the fission time on membrane templates of the indicated lipid composition. Data represents the mean $\pm \,$SD of 10 fission events. Statistical significance was estimated using unpaired Mann-Whitney’s test. **** denotes *p* < 0.0001 and *** denotes *p* = 0.0007.

Membrane fission requires an extreme degree of localized constriction, which forces embedded lipids to adopt non-lamellar conformations^103^. In such a scenario, the presence of lipids with a positive spontaneous curvature, such as lysophospholipids, have been shown to delay the kinetics of fission^104,105^. DAG has a negative spontaneous curvature and inclusion of DAG in the membrane tubes could directly potentiate the kinetics of the Drp1-mediated fission reaction. Interestingly, in the absence of MFF, 5% DAG had no effect on Drp1-mediated fission of membrane templates with low (5%) CL concentrations, indicating that the facilitatory effects of DAG on Drp1 functions requires a minimum membrane concentration of CL (Fig. 4a, b). At high concentrations of CL (15%), the faciliatory effects of DAG could not be clearly deciphered, as all of the prepared templates already display robust fission (Fig. 4a, b). However, a significant stimulatory effect of DAG was readily apparent, even at the high 15% CL concentration, when the membrane fission kinetics were assessed. For these analyses, the cumulative number of fission events in response to flowing in Drp1 together with GTP are plotted across the various membrane tubes as a function of time. Evidently, the inclusion of 5% DAG significantly enhanced fission kinetics at both intermediate (10%) and high (15%) CL concentrations (Fig. 5a, b and Supplementary Movie 5). Bulk fission kinetics can be biased depending on whether the tubes formed using a particular lipid composition are systemically thinner or thicker. However, this was not the case, as random sampling showed a similar mean tube radius across these different SMrTs preparations (Fig. 5c).

To further evaluate the effects of DAG on the kinetics of Drp1-mediated membrane fission, we focused on analyzing single fission events on membrane tubes with high (15%) CL concentrations, since the observed Drp1-catalyzed fission was robust on these CL-containing templates in the presence of either DAG or PI (Fig. 5a, b). Flowing in GTP on GppNHp-bound Drp1 scaffolds caused further membrane constriction of the underlying tube, which is evident from a kymograph of a representative fission event acquired in the membrane fluorescence channel (Fig. 5d). We then used a calibration procedure^106,107^ to equate the membrane fluorescence signal into a measurement of the tube radius (Fig. 5e). This kinetic assessment delineates the entire mechanical pathway that leads to fission, wherein GTP hydrolysis causes the Drp1 scaffolds to constrict the tube and reach a characteristic pre-fission intermediate with a radius of ~ 5 nm prior to the final membrane scission (Fig. 5e). Interestingly, analysis of multiple single fission events revealed that the scaffolded tube radius attained with templates containing DAG was significantly lower and less variable (8.1 ± 1.6 nm; Fig. 5f) than in templates prepared with PI (11.9 ± 4.2 nm; Fig. 5f), which implies that DAG allows the Drp1 scaffolds to constrict membrane tubes to a more uniform and thinner dimension. However, the pre-fission tube radius that is reached upon GTP hydrolysis showed no significant differences between the DAG- (5.2 ± 0.8 nm; Fig. 5g) and PI-containing membrane templates (5.3 ± 0.8 nm; Fig. 5g). To better understand the influence of DAG on the fission reaction, we measured the fission time, which we define as the interval between the onset of GTP hydrolysis-induced constriction and the severing of the membrane tube^106,107^. Remarkably, the fission time on CL-containing tubes is significantly shorter with the inclusion of DAG (8.4 ± 1.3 s; Fig. 5h) than with the inclusion of PI (14.2 ± 2.6 s; Fig. 5h). Taken together, these results indicate that DAG facilitates the mechanochemical capacity of Drp1 for constriction and accelerates the membrane fission reaction.

### DAG production recruits endophilin B1 to facilitate OMM remodeling

**Fig. 6:**
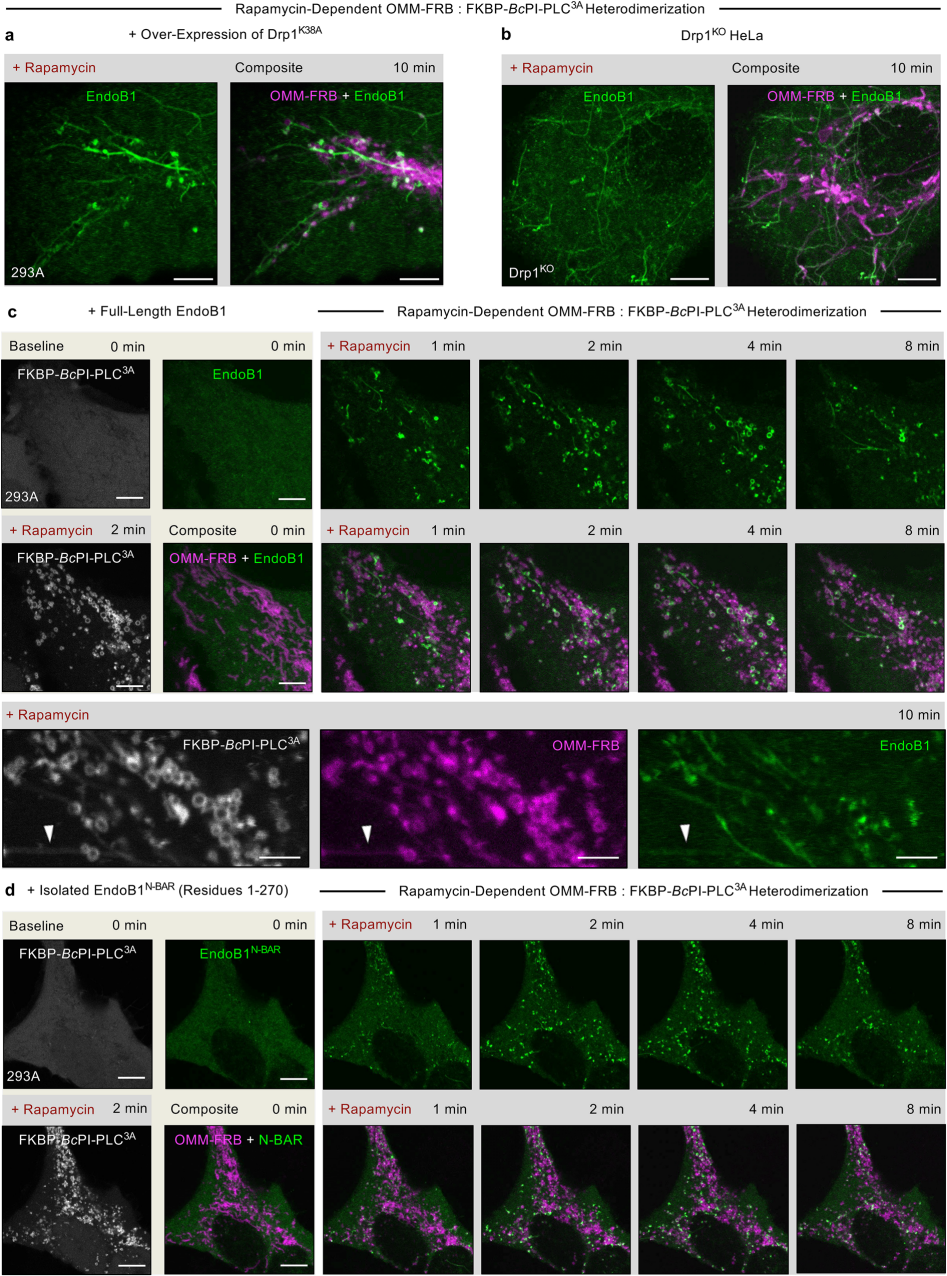
DAG production by FKBP-*Bc*PI-PLC^3A^ recruitment induces the rapid translocation of EndoB1 to the OMM. **a** Representative images of HEK293A cells (5 μm scale bar) showing the localization of the OMM-targeted FRB recruiter (OMM-FRB-mRFP, magenta), endophilin B1 (EndoB1-mEGFP, green), and the GTPase-deficient mutant of Drp1 (mCherry-Drp1^K38A^) after 10 min of rapamycin-induced (100 nM) recruitment of the catalytically active *Bc*PI-PLC^3A^ (emiRFP670-FKBP-*Bc*PI-PLC^3A^) to the cytosolic membrane leaflet of the mitochondria. The composite images present an overlay of the OMM-FRB together with EndoB1. In addition, a more detailed time series showing the individual fluorescent channels associated with the images presented here is provided as Supplementary Fig. 9a and Supplementary Movie 6. **b** Representative images of Drp1^KO^ HeLa cells (5 μm scale bar) showing the localization of the OMM-targeted FRB recruiter (OMM-FRB-mRFP, magenta) and EndoB1 (EndoB1-mEGFP, green) after 10 min of rapamycin-induced (100 nM) recruitment of the catalytically active *Bc*PI-PLC^3A^ (emiRFP670-FKBP-*Bc*PI-PLC^3A^) to the cytosolic membrane leaflet of the mitochondria. The composite images present an overlay of the OMM-FRB together with EndoB1. The complete presentation of the individual fluorescent channels from this representative image is provided as Supplementary Fig. 9b, while a complete time series for a replicate of this experiment is provided as Supplementary Movie 7. **c**, **d** Representative images of HEK293A cells (5 μm scale bar) showing the localization of the OMM-targeted FRB recruiter (OMM-FRB-ECFP, magenta) and either EndoB1 (**c**; EndoB1-mEGFP, green) or the isolated N-BAR domain (**d**; residues 1–270, EndoB1^N-BAR^-mEGFP, green) in response to rapamycin-induced (100 nM) recruitment of the catalytically active *Bc*PI-PLC^3A^ (mRFP-FKBP-*Bc*PI-PLC^3A^, gray) to the cytosolic membrane leaflet of the mitochondria. The composite images shown present an overlay of the OMM-FRB together with EndoB1. In addition, the complete time series (Supplementary Movie 8) as well as enlarged still images (bottom row panels; 2.5 μm scale bar) are presented for (**c**), which shows *Bc*PI-PLC^3A^-induced translocation of the full-length EndoB1 to the OMM.

The striking observation that *Bc*PI-PLC^3A^-induced DAG generation creates hyper-constrictions of the OMM in Drp1^KO^ cells and cells expressing Drp1^K38A^ (Fig. 2a, b and Supplementary Movie 3), suggested that other effectors may function in concert with Drp1 to regulate mitochondrial membrane remodeling. Among the established superfamilies of membrane-shaping proteins, the BAR^108,109^ domain-containing protein EndoB1^110,111^ has been previously shown to be required for the maintenance of mitochondrial morphology^112^. Mitochondrial accumulation of EndoB1 has been demonstrated in response to chronic treatments with pro-apoptotic agents or following the induction of bulk mitophagy^34,112,113^, but the molecular cues that recruit EndoB1 to the mitochondria remain unclear. Furthermore, in vitro, EndoB1 has been shown to bind and tubulate liposomes mimicking the OMM^114–116^. Given this background, we wondered if the hyper-constriction of the OMM that is evident upon acute generation of DAG in the absence of Drp1 functions was linked to the recruitment of EndoB1. Indeed, in both HEK293A cells over-expressing the Drp1^K38A^ (Fig. 6a, Supplementary Fig. 9a and Supplementary Movie 6) and Drp1^KO^ HeLa cells (Fig. 6b, Supplementary Fig. 9b and Supplementary Movie 7), fluorescently-tagged EndoB1 rapidly translocated to constrictions of the mitochondrial surface upon acute recruitment of FKBP-*Bc*PI-PLC^3A^. Remarkably, FKBP-*Bc*PI-PLC^3A^ also induced EndoB1 translocation to the OMM in wild-type HEK293A cells and coincided with prominent tubulations of the OMM, which were eventually resolved as the induced mitochondrial fragmentation progressed (Fig. 6c and Supplementary Movie 8), indicating that recruitment of EndoB1 can deform the OMM. The isolated N-BAR domain of EndoB1 (Residues 1–270; EndoB1^N-BAR^-mEGFP) similarly showed OMM enrichment following FKBP-*Bc*PI-PLC^3A^ recruitment (Fig. 6d).

### DAG promotes the assembly of EndoB isoforms on supported membrane templates

**Fig. 7:**
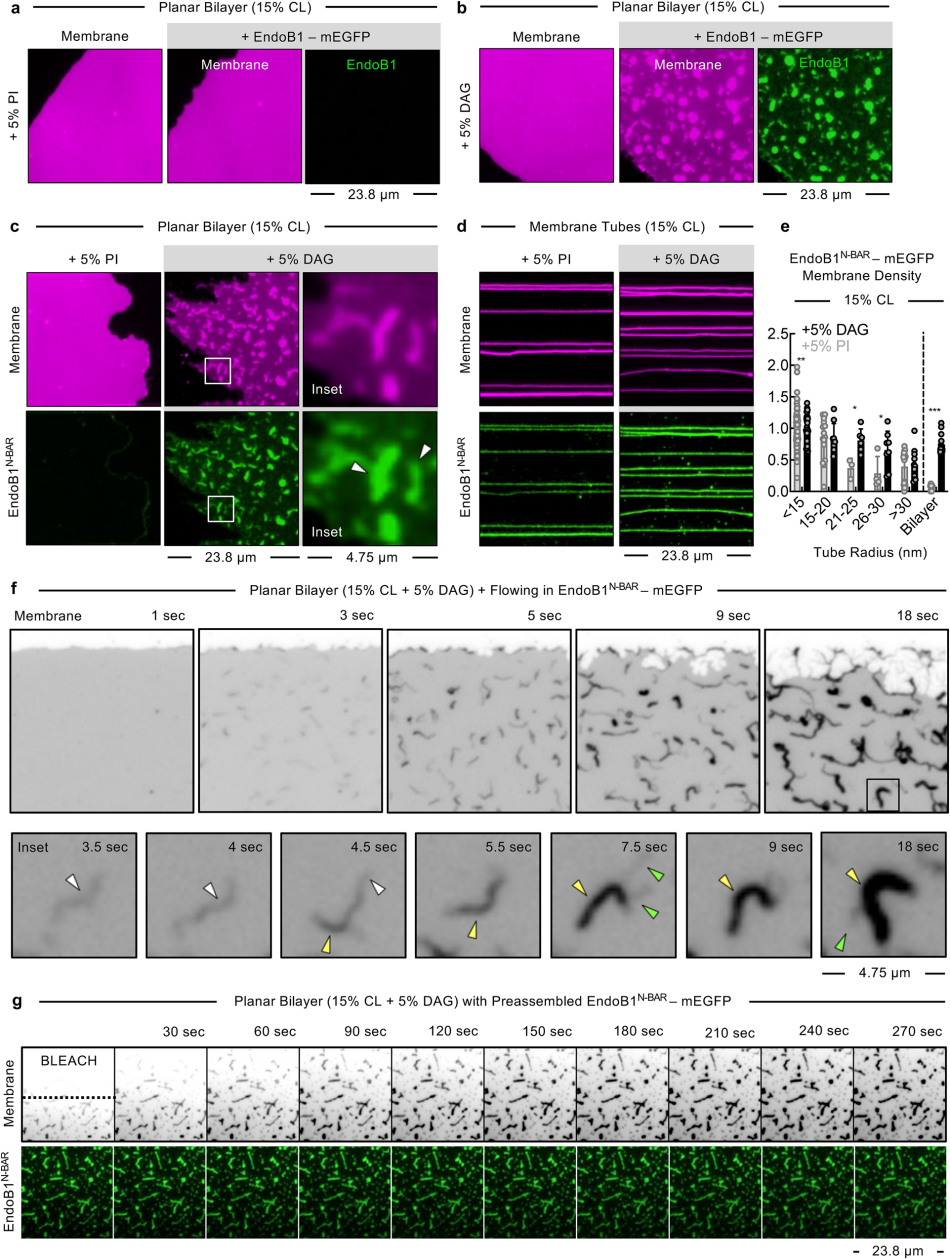
DAG facilitates membrane binding and tubulation by EndoB1. **a**, **b** Representative images of a planar bilayer (magenta) containing 5% PI (**a**) or 5% DAG (**b**) with 15% CL, before and after incubation with EndoB1-mEGFP (green). **c** Representative images of a planar bilayer (magenta) of the indicated lipid composition after incubation with EndoB1^N-BAR^ (Residues 1–270)-mEGFP (green). White arrows in magnified panels mark protein clusters that coincide with regions of high membrane fluorescence indicating tubulation. **d** Representative images of membrane tubes (magenta) of the indicated lipid composition incubated with EndoB1^N-BAR^-mEGFP (green). **e** Membrane densities of EndoB1^N-BAR^-mEGFP are reported as the ratio of EGFP and membrane fluorescence across a range of tube sizes as well as on the planar bilayer island of the indicated lipid composition. Data are normalized to the membrane density of EndoB1^N-BAR^-mEGFP on tubes of < 15 nm radius containing 5% DAG and 15% CL and represents the mean ± SD of 63 tubes and 31 different regions on planar bilayers for DAG-containing and 106 tubes and 35 different regions on planar bilayers for PI-containing membranes. Statistical significance was estimated using unpaired Mann-Whitney’s test. *** denotes *p* < 0.0001, ** denotes *p* = 0.074, * denotes *p* = 0.0238. **f** Representative time-lapse images of a planar bilayer responding to flowing EndoB1^N-BAR^-mEGFP, which are also presented as Supplementary Movie 9. The inset shows a magnification of a single representative tubulation event. Images are acquired in the membrane fluorescence channel and are inverted in contrast for clarity. White arrows mark a single tubule, yellow arrows mark events of coiling of the tubule, and green arrows mark the coalescence of other single tubules in the vicinity. **g** Representative time-lapse images showing fluorescence recovery after photobleaching the intrinsic fluorescent lipid probe in a large area of the planar bilayer displaying EndoB1^N-BAR^-mEGFP-coated tubules, which are also presented as Supplementary Movie 10. The region above the dotted line is where the lipid probe was bleached.

We tested if the observed translocation of EndoB1 to the OMM in response to the acute production of DAG, as well as the resulting membrane tubulation, reflects the ability of EndoB1 to directly bind and remodel DAG-containing membranes. For this, we first focused on planar bilayers containing 5% PI or 5% DAG in a background of DOPC together with 15% CL. EndoB1-mEGFP showed negligeable binding to templates containing 15% CL alone, and the presence of 5% PI did not have any effect on membrane residency (Fig. 7a). Remarkably, templates containing 5% DAG recruited substantial levels of EndoB1-mEGFP (Fig. 7b) and the clusters of EndoB1-mEGFP coincided with brighter membrane fluorescence, which suggests that DAG facilitates both the membrane recruitment of EndoB1 as well as subsequent remodeling of the planar bilayer (Fig. 7b). The isolated EndoB1^N-BAR^-mEGFP also showed DAG-specific membrane binding and induced tubulation of the planar bilayer (Fig. 7c, white arrowheads). Next, we tested how EndoB1 behaved on pre-formed membrane tubes. The binding of EndoB1^N-BAR^-EGFP to membrane tubes without DAG or PI was negligeable. Surprisingly, EndoB1^N-BAR^-mEGFP showed binding to curved tubes containing 5% DAG as well as 5% PI in a background of DOPC with 15% CL (Fig. 7d, e). Quantitation of membrane densities showed that EndoB1^N-BAR^-mEGFP bound DAG-containing curved membrane tubes and planar bilayer equally well. However, on PI-containing membranes, binding was restricted only to curved membrane tubes, with the planar bilayer showing negligeable protein density (Fig. 7e). These results indicate that the recruitment of the EndoB1^N-BAR^ depends on both the lipid composition and membrane curvature. Consequently, on PI-containing membranes, EndoB1 likely only senses membrane curvature, while on DAG-containing membranes, it can both sense and generate curvature. Together, these results suggest that DAG generation acts as a potent signal that recruits EndoB1 to the planar regions of the mitochondria and cause them to deform and undergo tubulation.

To better understand the membrane tubulation process, we imaged the planar bilayer containing 5% DAG and 15% CL as it was exposed to EndoB1^N-BAR^-mEGFP at a high frame rate. Seconds after flowing in the EndoB1^N-BAR^-mEGFP, numerous faint and spindly membrane tubules could be observed sprouting from the bilayer, which then grew both in length and intensity before finally coalescing into bright stubs (Fig. 7f and Supplementary Movie 9). A closer examination of the kinetics of this membrane deformation revealed that the process begins with the generation of a single tubule (Fig. 7f, inset, white arrowhead), which then starts acquiring varicosities along its length (Fig. 7f, inset, yellow arrowheads), likely reflecting regions where the tubule has coiled around itself. Subsequently, other tubules in the vicinity (Fig. 7f, inset, green arrowheads) then appear to bind together and ultimately coalesce into bright bundles of tubules. We analyzed single tubules before they underwent coiling and, using the established calibration procedure (*see Methods*), we estimated their size to be 15.5 ± 2.8 nm radius (*n* = 23), which is consistent with estimates reported from cryo-EM reconstructions of EndoB1^N-BAR^-coated tubules^116^. Members of the endophilin A (EndoA) family, which are involved in the formation of endocytic transport carriers at the plasma membrane (PM)^117,118^, have been reported to impose a barrier to the diffusion of lipids within the membrane^119^. However, other BAR domain-containing proteins have been shown to only restrict diffusion of specific phosphoinositide lipids that they bind to directly and do not affect the movement of bulk lipids^120^. We assessed the bulk lipid diffusion of EndoB1-coated tubules formed from planar bilayers using fluorescence recovery after photobleaching (FRAP). Photobleaching the fluorescent TR-DHPE lipid in a large area of the planar bilayer showed similar recovery kinetics for both the tubules and underlying bilayer (Fig. 7g and Supplementary Movie 10). This indicates that the assembled EndoB1 scaffold does not impose a diffusion barrier to bulk membrane lipids and behaves in a manner that is different from that reported for EndoA2.

We also examined the effects of local DAG production on the localization of the closely related EndoB1 homolog, EndoB2^111^ (Supplementary Fig. 10a). Notably, FKBP-*Bc*PI-PLC^3A^ recruitment similarly resulted in the dynamic assembly of EndoB2 on the OMM, however the extent of the membrane tubulations typically observed were generally reduced and more transient when compared to those induced by EndoB1 (Supplementary Fig. 10b). This difference in membrane localization is best exemplified in cells co-overexpressing both EndoB1 and EndoB2 (Supplementary Fig. 10b). This remarkable translocation of both EndoB isoforms and the associated constrictions of the OMM suggest that acute DAG generation-induced membrane assembly of EndoB oligomers can transiently stabilize OMM fission intermediates or potentially induce localized tubulations of the OMM. Consistent with the data from live-cell imaging, the N-BAR domain of EndoB2 (Residues 1–292; EndoB2^N-BAR^-mEGFP) also displayed DAG-specific binding (Supplementary Fig. 10c) on planar bilayers but formed smaller and self-limited clusters that showed variable degrees of membrane remodeling (Supplementary Fig. 10c, white arrowheads). On pre-formed membrane tubes, EndoB2^N-BAR^-mEGFP showed negligeable binding to PI-containing templates and significantly lower levels of binding in the presence of DAG compared to what was observed with EndoB1^N-BAR^-mEGFP (Supplementary Fig. 10d, e), which supports the conclusion that EndoB2^N-BAR^-mEGFP is generally less efficient than EndoB1^N-BAR^-mEGFP for membrane binding. Together, this difference signifies that EndoB2 has a reduced ability to bind and remodel the membrane bilayer, which is consistent with the relatively less prominent and more transient recruitment of EndoB2 to the OMM in response to *Bc*PI-PLC^3A^-induced DAG production in HEK293A cells (Supplementary Fig. 10b).

### EndoB1 does not directly bind Drp1 but interferes with Drp1-mediated fission

Endophilins contain a characteristic N-BAR domain at the N-terminus and an SH3 domain at the C-terminus that are connected by a disordered linker in between^110,111^. Direct interactions between the SH3 domain of EndoA1 and the proline-rich domain (PRD) of endocytic dynamins, Dnm1 and Dnm2^121–123^, helps to orchestrate the ordered recruitment of the fission machinery at constricted membrane necks formed on the PM^124^. However, unlike the EndoA subfamily, EndoB isoforms have not been reported to directly bind to dynamin family proteins. Given the fact that both EndoB1/2 and Drp1 get recruited to the OMM in response to DAG production, we wondered if these proteins directly interact. To assess this possibility in live cells, we used a rapamycin-induced rerouting approach that allows for investigations of protein-protein interactions using folded protein domains in real-time^125^. As a control for this approach, rapamycin-dependent rerouting of the FKBP-tagged extended C-terminus of EndoA1 (Residues 251–352; FKBP-EndoA1^CT^) to the OMM rapidly rerouted Dnm2-EGFP to the OMM, which is consistent with the well-documented interaction between the two proteins (Supplementary Fig. 11a). In contrast, acute mitochondrial rerouting of FKBP-EndoB1^CT^ (Residues 269–365) failed to alter the subcellular localization of either Drp1 (Supplementary Fig. 11b) or Dnm2 (Supplementary Fig. 11c). These data suggest that, unlike EndoA1, the extended C-terminus of EndoB1 does not directly interact with Drp1 or Dnm2. In addition, since over-expression of Drp1^K38A^ or Drp1^KO^ both enhanced the abundance and stability of EndoB1-coated OMM tubules (Fig. 6a, b, Supplementary Fig. 9a, b and Supplementary Movies 6 and 7), the cross-talk between the membrane remodeling activities of EndoB1 and Drp1 are likely independent of a direct interaction. In the endocytic pathway, Dnm2 displays an extreme sensitivity to membrane curvature and can only bind and sever highly-curved membrane tubes^126^, but Dnm2 can be made to vesiculate planar membranes by introducing the curvature-generating N-BAR domain of EndoA1^127,128^. Since the EndoA1^N-BAR^ lacks the SH3 domain that interfaces with the PRD of Dnm2, these data indicate that even in the absence of direct interaction, curvature-generating proteins can synergize with dynamin-like proteins to facilitate membrane fission. Therefore, even without direct interaction, Drp1 and EndoB1 could still work together to remodel the OMM, as Drp1 also exhibits strong sensitivity to membrane curvature, which is induced by EndoB1 self-assembly. Given these considerations, we wondered if the tubules formed by EndoB1^N-BAR^ from DAG-containing planar bilayers could act as a template for Drp1-catalyzed fission, especially considering that the ~ 15 nm radius of the EndoB1^N-BAR^-coated tubules fall within the compliant size range for Drp1-catalyzed fission (Fig. 5a–c). Flowing a mixture of EndoB1^N-BAR^-mEGFP and Drp1 mixed with GTP onto the planar bilayer sprouted membrane tubules that were decorated with EndoB1^N-BAR^-mEGFP (Supplementary Fig. 11d). However, fission was not readily apparent, as the EndoB1^N-BAR^-mEGFP-enriched tubules continued to grow and eventually coiled (Supplementary Fig. 11d and Supplementary Movie 11) with kinetics similar to what was observed in the absence of Drp1 (Fig. 7c, f). It is possible that the membrane tubules could have undergone fission, but their tendency to coil around each other could mask this effect by tethering them to the bilayer. To assess this, we again used FRAP and bleached the intrinsic membrane lipid probe to monitor the kinetics of the fluorescence recovery. These experiments revealed complete recovery of the membrane fluorescence within the EndoB1^N-BAR^-coated tubules, indicating that they were still connected to the underlying planar bilayer (Supplementary Fig. S11e and Supplementary Movie 12). Together, these results indicate that Drp1 lacks the capacity to efficiently sever tubules generated by and coated with EndoB1^N-BAR^. It is likely that the tendency of the EndoB1^N-BAR^-coated tubules to rapidly coil could sterically hinder the ability of Drp1 to bind the membrane, which would thereby prevent Drp1-catalyzed fission.

In addition to the remodeling of planar bilayers, we also assessed the combined actions of mixtures containing both the EndoB1^N-BAR^ and Drp1 on pre-formed membrane tubes containing 5% DAG or 5% PI together with 15% CL. For these studies, we flowed Drp1 mixed with GTP and EndoB1^N-BAR^-mEGFP onto the preformed membrane tubes. Treatments with Drp1 and GTP alone showed robust fission of the membrane tubes, however the presence of EndoB1^N-BAR^-mEGFP strongly inhibited membrane fission (Supplementary Fig. 11f). This was quite surprising because these tubes contained high (15%) concentrations of CL, which is normally extremely permissive to fission. The robust inhibition of Drp1-mediated fission suggests that EndoB1^N-BAR^ assembly likely directly competes with Drp1 to access the membrane surface. Consistent with this possibility, previous studies on the functional interactions between EndoA2 and Dnm2 in the context of endocytosis have shown inhibition of dynamin-dependent membrane fission by the presence of endophilin in vitro and upon over-expression in cells^129^. Thus, the global fragmentation of the mitochondrial network, even upon over-expression of EndoB1, could been mediated by Drp1 that is pre-assembled on the OMM surface. Consequently, the unique role of EndoB is likely to respond to local changes in lipid composition mediate the initial deformation of planar regions of the mitochondria, and restrict fission to sites that have preassembled Drp1, which may explain the importance of OMM-localized Drp1 receptors like MFF.

### Amphipathic H_0_ and H_1i_ helices facilitate DAG sensing by EndoB1

**Fig. 8:**
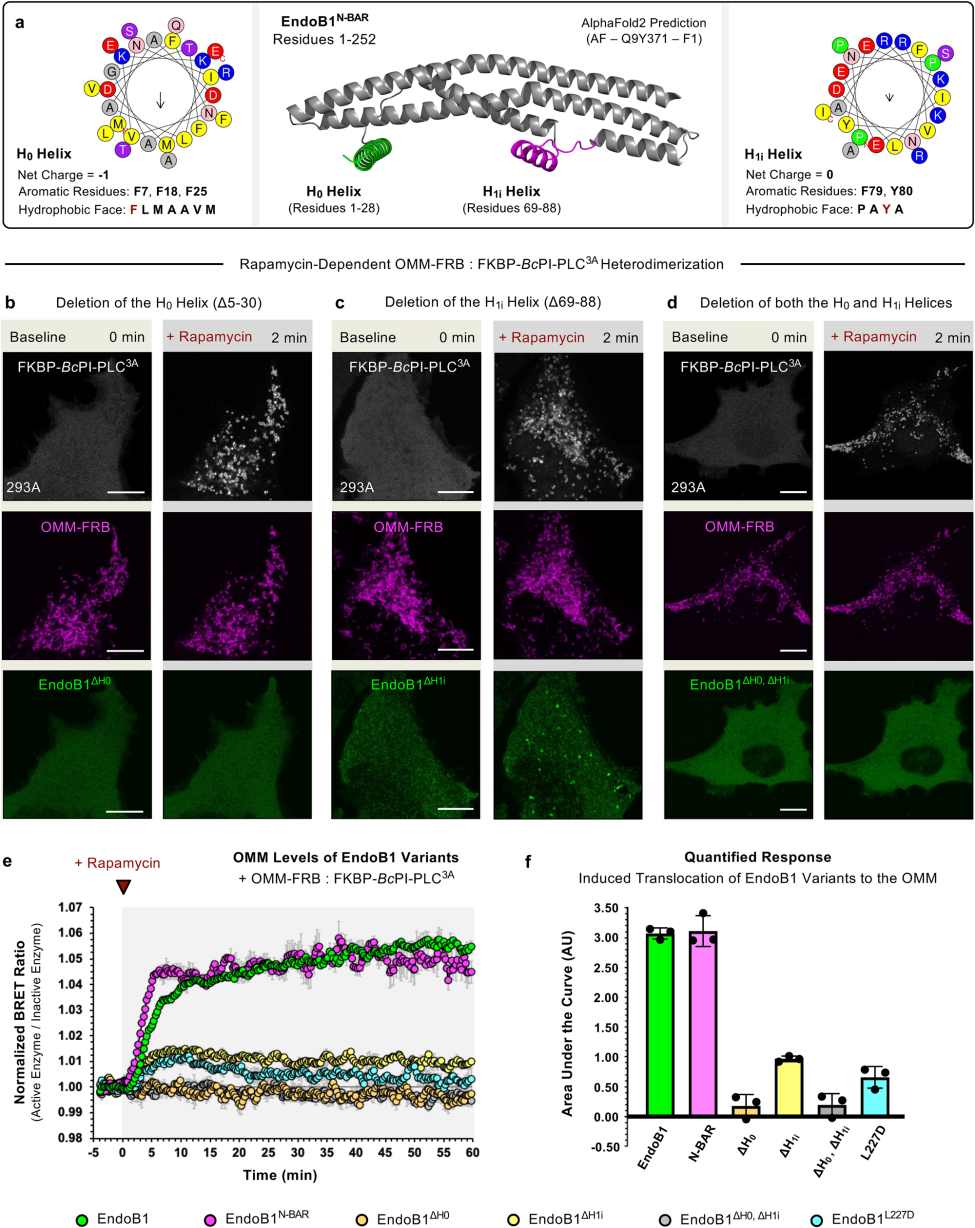
Deletion of the amphipathic H_0_ and H_1i_ helices or disruption of self-assembly significantly reduces *Bc*PI-PLC^3A^-induced translocation of EndoB1 to the OMM. **a** Domain architecture of the isolated N-BAR domain from EndoB1 (middle panel; AlphaFold2 prediction AF-Q9Y371-F1; DeepMind)^176,177^ presented along with a comparison of the physiochemical properties (HeliQuest)^178^ of the N-terminal amphipathic H_0_ (right side panel) and H_1i_ (left side panel) helices of EndoB1 (UniProt: Q9Y371). Protein structures were prepared using the PyMOL Molecular Graphics System (Version 3.0; Schrödinger, LLC). **b**–**d** Representative images of HEK293A cells (10 μm scale bar) showing the localization of the OMM-targeted FRB recruiter (OMM-FRB-ECFP, magenta) and EndoB1 mutants with deletions of either the H_0_ helix (**b**, EndoB1^△H0^ (△Residues 5–30)-mEGFP, green), H_1i_ helix (**c**, EndoB1^△H1i^ (△Residues 69–88)-mEGFP, green), or both amphipathic segments (**d**, EndoB1^△H0,△H1i^ (△Residues 5–30 + △Residues 69–88)-mEGFP, green) before (top row panels) or 10 min after (bottom row panels) rapamycin-induced (100 nM) recruitment of the catalytically active *Bc*PI-PLC^3A^ (mRFP-FKBP-*Bc*PI-PLC^3A^, gray) to the cytosolic membrane leaflet of the mitochondria. **e** Kinetics and (**f**) quantified area under the curve measured for the OMM localization of the wild-type EndoB1 (green), isolated N-BAR domain (EndoB1^N-BAR^, Residues 1–270, magenta), L227D point-mutant (EndoB1^L227D^, blue), as well as the EndoB1^△H0^ (△Residues 5–30, orange), EndoB1^△H1i^ (△Residues 69–88, yellow), and EndoB1^△H0,△H1i^ (△Residues 5–30 + △Residues 69–88, gray) deletion mutants in HEK293A cells following recruitment of FKBP-*Bc*PI-PLC^3A^ to the cytosolic membrane leaflet of the mitochondria, as measured using EndoB1 variant-specific OMM-EndoB1^BRET^ biosensors (AKAP^TM^-mVenus-tPT2A-EndoB1(or variants)-sLuc). To allow for direct comparisons between OMM-EndoB^BRET^ variants, the BRET values obtained for recruitment of the active iRFP-FKBP-*Bc*PI-PLC^3A^ enzyme were normalized to measurements made using the corresponding catalytically-inactive iRFP-FKBP-*Bc*PI-PLC^DEAD^ control. BRET measurements are presented as mean values ± SEM from three independent experiments carried out using triplicate wells.

EndoB1 contains two amphipathic segments, termed the H_0_ and H_1i_ helices, which are closely associated with the BAR domain (Fig. 8a) and are thought to facilitate membrane binding as well as participate in higher-order oligomerization on tubulated membranes^116^. We tested the requirement of these amphipathic helices in recruiting and subsequent remodeling of OMM upon acute DAG generation (Fig. 8b–d). To facilitate quantitative measurements of EndoB translocation after FKBP-*Bc*PI-PLC^3A^ recruitment, we designed BRET-based biosensors to monitor the enrichment of EndoB variants specifically within the cytosolic leaflet of the OMM at the cell population scale (Fig. 8e, f). These single-plasmid reporters are built using an OMM-targeted mVenus together with EndoB1 variants that are fused to super *Renilla* luciferase (AKAP^TM^-mVenus-tPT2A-EndoB1(or variant)-sLuc). Molecular proximity between the resident mVenus and the EndoB1-sLuc construct on the mitochondrial surface can be captured as an increase in resonance energy transfer that is measured by the ratio of the mVenus to sLuc emission intensities. Expression of EndoB1 (Fig. 6c) or the isolated EndoB1^N-BAR^ (Residues 1–270; Fig. 6d) domain showed that these proteins translocate to the OMM in response to the acute recruitment of FKBP-*Bc*PI-PLC^3A^, as indicated by a pronounced increase in the measured BRET ratio (Fig. 8e, f). Remarkably, deletion of the H_0_ helix (^△H0^, △Residues 5–30) completely abolished translocation of EndoB1 to the OMM in response to acute FKBP-*Bc*PI-PLC^3A^ recruitment, which is shown both by live-cell imaging and in the quantified BRET ratio (Fig. 8b, e, f). On the other hand, deletion of the H_1i_ helix (^△H1i^, △Residues 69–88), greatly reduced, but did not completely abolish the *Bc*PI-PLC^3A^-induced recruitment to the OMM (Fig. 8c, e, f). However, the characteristic tubulations of the OMM that are associated with the membrane enrichment of the wild-type EndoB1 were diminished with the EndoB1^△H1i^ deletion mutant (Fig. 8c). The isolated BAR domain of EndoB1 (ΔH_0_, Residues 5–30, and ΔH_1i_, Residues 69–88; EndoB1^ΔH0,ΔH1i^) also does not translocate to the OMM in response to acute DAG production (Fig. 8d, e, f), which suggests that the presumed curvature-sensing domain of EndoB1 alone is not sufficient for membrane recruitment and assembly. These data demonstrate that the H_0_ helix serves as the primary driving force for the DAG-induced translocation of EndoB1 to the OMM while suggesting that both the H_0_ and H_1i_ helices contribute to the acute membrane remodeling induced by localized EndoB1 self-assembly. As a result, oligomerization of EndoB1 is likely to rapidly increase the avidity of the growing EndoB1 complex^130^ and, therefore, is also likely to significantly contribute to OMM remodeling. To test this directly, we introduced a point mutation within the BAR domain that alters the charge complementarity of a conserved dimer interface found in all endophilins (Supplementary Fig. 12a; L227D, EndoB1 residue numbering)^131^. Remarkably, EndoB1^L227D^ showed a significantly blunted translocation to the OMM upon acute DAG production (Supplementary Fig. 12b and Fig. 8e, f). Moreover, the small and transient membrane enrichment of the L227D mutant also failed to elicit the pronounced tubulations of the OMM that were observed with the wild-type EndoB1 (Supplementary Fig. 12b). To assess EndoB1 oligomerization, we again used the rapamycin-induced rerouting approach. Specifically, we generated an FKBP-tagged EndoB1 variant (EndoB1-FKBP-mRFP) that could be acutely recruited to the OMM and monitored the localization of EndoB1-mEGFP upon rapamycin treatment. Consistent with the known homo-dimerization of EndoB1, the cytosolic EndoB1-mEGFP was rapidly co-recruited to the OMM upon rapamycin-dependent translocation of EndoB1-FKBP-mRFP, thereby indicating that both tagged variants of EndoB1 can co-oligomerize in response to membrane-induced self-assembly (Supplementary Fig. 12c). Interestingly, while the forced assembly of EndoB1 drastically altered the mitochondrial network morphology (Supplementary Movie 13), without the local production of DAG, we did not observe hyper-constrictions or tubulations of the OMM, nor could we detect bulk changes in mitochondrial fission. Alternatively, upon rapamycin treatment, EndoB1^L227D^-mEGFP was not co-recruited to the OMM by EndoB1-FKBP-mRFP (Supplementary Fig. 12d) and, in the inverse experiment, OMM rerouting of EndoB1^L227D^-FKBP-mRFP similarly failed to co-recruit EndoB1-mEGFP (Supplementary Fig. 12e); thereby confirming that the L227D point mutation in the dimer interface effectively abolishes the ability of EndoB1 to oligomerize. These findings indicate that while the H_0_ and H_1i_ helices are essential for the initial membrane recognition by EndoB1, self-assembly of the growing EndoB1 oligomer drives the resulting membrane deformation. Formation of higher-order EndoB1 complexes are likely to prolong the membrane residency through interactions with the extended N-BAR domain^130^, which simultaneously functions to increase the immersion depth of the H_0_ helix within the membrane interface^132^. Nascent changes to the local membrane curvature in response to EndoB1 binding may further reinforce the intermolecular assembly of EndoB1 subunits into the growing helical scaffold^133^ to promote OMM remodeling.

## Discussion

In this study, we used acute modifications of the OMM composition to investigate the role of specific lipids in regulating mitochondrial morphology. We complemented these unique live-cell approaches with a powerful in vitro reconstitution system consisting of topologically diverse supported membrane templates, to understand the mechanistic basis for the lipid-driven activities of unique mitochondrial membrane-shaping proteins. Our results reveal that the localized conversion of PI to DAG on the OMM initiates and sustains two distinct membrane remodeling processes on the mitochondria, which collectively lead to acute tubulation and fission of the organelle. The mechanistic basis for these phenomena lies in the ability of DAG to directly influence the recruitment and subsequent molecular activities of membrane-shaping proteins that promote mitochondrial remodeling. Foremost, the bulk mitochondrial fragmentation observed upon acute DAG production on the OMM was dependent on Drp1, as Drp1^KO^ or over-expression of dominant-negative Drp1 mutants both prevented the DAG-induced OMM fission. Upon fragmentation of the network, mitochondria lose the pre-assembled Drp1-enriched foci, which likely indicates active depolymerization and detachment from the OMM surface of Drp1 following the induced fission. Previous reports have suggested the involvement of another dynamin-like protein, Dnm2, in mitochondrial division^83^. However, our results indicate that Dnm2 does not get recruited to the OMM in response to local DAG production and, unlike Drp1, over-expression of Dnm2 did not rescue the mitochondrial fission defect observed following DAG generation in the OMM of Drp1^KO^ cells.

In vitro reconstitution using OMM-mimetic templates suggests that incorporation of DAG into CL-containing membranes causes a dramatic stimulation of Drp1-catalyzed fission. This results from an apparent synergy between DAG and CL, which facilitates all aspects of Drp1-dependent membrane remodeling, including both enhancing membrane binding as well as potentiating the GTPase-induced constriction and downstream fission. In cells, Drp1 is generally localized as punctate structures on the OMM that appear to become brighter upon acute DAG generation before rapidly disassembling upon mitochondrial fission (Fig. 3, Supplementary Fig. 3 and Supplementary Movies 1, 2, and 4). Notably, in vitro studies similarly show that Drp1 assemblies depolymerizes following GTP hydrolysis^134^. Our reconstitution data indicates that even low levels of DAG not only recruit more Drp1 to the membrane but also potentiates its mechanochemical activity to significantly enhance fission. Consequently, the localized change in lipid composition can potentially activate the OMM-localized pool of pre-assembled Drp1 to rapidly engage with the membrane directly, which can then recruit additional Drp1 from the cytosol to drive OMM constriction and fission leading to the widespread fragmentation of the mitochondrial network. Structural studies suggest that GTP hydrolysis induces a conformational rearrangement in Drp1 that results in the dissociation of bound adapter proteins and causes an associated shortening of Drp1 filaments into the closed rings that directly interface with the highly constricted underlying membrane^135^. Interestingly, the helical stalk of Drp1 selectively interacts with OMM-anchored adapters that facilitate its OMM localization^8^, while the binding and insertion of residues in the unstructured variable domain (VD) are necessary for its interaction with CL-containing membranes^136^. Positively charged lysine residues within the VD have been shown to facilitate the binding of Drp1 to anionic lipids^21,22^. More recently, however, the VD has been suggested to interact with CL-containing membranes through hydrophobic interactions^26^. Consequently, it is possible that the VD may be involved in sensing local packing defects created by DAG to potentiate the interfacial binding of Drp1 to CL-containing membranes.

Rapid generation of DAG in the OMM of Drp1^KO^ cells caused extensive mitochondrial constrictions and tubulations, suggesting that other membrane-shaping proteins may also respond to increased DAG levels in the OMM. We identified the BAR-domain containing proteins, EndoB1 and EndoB2, as curvature-sensitive effectors responsive to DAG generation in the OMM. However, relative to EndoB2, EndoB1 showed higher sensitivity to membrane DAG content and was more effective in promoting membrane remodeling. Interestingly, the N-BAR domain of EndoB2 contains a unique insertion within the helix 2-helix 3 linker that effectively lengthens the crescent formed by the anti-parallel helical bundle. This subtle difference in the size of the membrane-binding face could conceivably change the preference of growing oligomeric EndoB2 complexes towards less extreme membrane curvatures, such as those formed during the earliest phases of membrane bending. Furthermore, we show that the amphipathic H_0_ helix of EndoB1 is essential for DAG-dependent recruitment to the OMM, whereas deletion of the H_1i_ helix lowered the extent of the DAG-induced translocation, but did not abolish it entirely. These findings are consistent with previous in vitro experiments that suggested that the H_0_ helix is required for EndoB1 binding and tubulation of liposomes^116^, which collectively supports an essential role for the H_0_ helix of EndoB1 for sensing packing defects in DAG-containing membranes. Taken together, our work suggests a common mechanism among two critical membrane remodeling proteins, Drp1 and EndoB, in their ability to sense DAG within the membrane. Importantly, this DAG-mediated regulation of EndoB1 self-assembly on the OMM does not require Drp1. Moreover, our in vitro data further indicate that EndoB1/2 are uniquely capable of sensing DAG within a CL-containing membrane. Binding to these membranes cause them to tubulate the underlying membrane, and our results are the first to capture this process in real time. Thus, the acute production of DAG could recruit EndoB1, and to a lesser extent EndoB2, to relatively planar regions on the OMM and initiate the observed remodeling. Because EndoB1 can also bind PI on curved membrane tubes, this would result in a feed-forward cascade causing further recruitment of EndoB1, resulting in extensive tubulation of the OMM. EndoB2-induced tubulation would be restricted because of its lower affinity for DAG- and CL-containing membranes. Given that both the tubulating and fission machineries get recruited to the mitochondria upon DAG production, we wondered if they worked synergistically to impact mitochondrial remodeling, however, our results do not support a direct inter-regulation between Drp1 and EndoB1. In terms of kinetics, mitochondrial fission is likely to be a faster process than OMM tubulation, as the OMM-tethered Drp1 is GTP-bound^135^ and primed for fission, while tubulation requires the recruitment and self-assembly of cytosolic EndoB on the OMM. However, once recruited, EndoB1 has the ability to tubulate the OMM, and these tubules would persist, as they are resilient to fission by Drp1. This explains the prevalence of the acute OMM constrictions observed in cells over-expressing EndoB isoforms in response to the acute generation of DAG. Thus, the density and extent of EndoB-induced OMM remodeling and tubulation would depend directly on the availability of DAG within the OMM, which in turn would be influenced by competition with Drp1 for highly curved OMM segments. Together, our results explain the mechanistic basis for the observed tubulation and mitochondrial fragmentation upon acute generation of DAG and emphasize that the stoichiometries of EndoB and Drp1 are likely to be critical for the concerted action of these membrane-shaping proteins to promote OMM tubulation and fission.

Our findings indicate that DAG production in the OMM has significant consequences for the mitochondrial morphology and previous reports have suggested mechanisms for DAG production under normal physiology or pathological settings. In particular, localized production of DAG in the OMM has been inferred from reports showing changes to mitochondrial morphology by alterations to the relative expression of the Lipin family of PA-selective phosphatases^52,54,60^, which would regulate DAG levels through direct dephosphorylation of PA. However, despite the reported involvement of Lipins as an important regulator of cellular lipid metabolism and mitochondrial dynamics, defining the biosynthetic source of the PA that is generated in the OMM for Lipin activity has proven to be extremely challenging. Earlier studies identified an OMM-localized phospholipase D (PLD)-like phosphodiesterase, PLD6, which was proposed to increase mitochondrial PA levels by direct hydrolysis of CL^51–54^. However, the PA-generating hydrolytic activity attributed to this enzyme is not supported by in vitro assays using purified mammalian PLD6 or by structural descriptions of the enzyme active site in eukaryotic PLD6 homologs^137^. Alternatively, in addition to the acute enzymatic production of DAG or PA directly within the OMM, the local delivery of membrane lipids to the OMM may also be facilitated by transport at inter-organelle contacts with the ER; which serves as the major site of de novo glycerophospholipid biosynthesis as well as neutral lipid storage in mammalian cells^138–140^. In fact, membrane contacts between the OMM and ER are already established regulators of mitochondrial dynamics^141–145^, and potential regulatory roles for the supply of ER-derived lipids during both the mitochondrial fission and fusion processes has been proposed by numerous groups^14–16,41,146–151^. Since mitochondria do not receive lipids through vesicular trafficking, the delivery of DAG or PI to the OMM likely involves non-vesicular lipid transport^152,153^, including the possible involvement of cytosolic PI transfer proteins^154,155^. Understanding how the lipid composition of the OMM is actively regulated, especially with regard to the local DAG content, remains an exciting unanswered question.

Finally, a recent report suggests that DAG produced by Lipin1 can regulate PINK1/Parkin-mediated mitophagy^60^. Drp1-dependent OMM fission is also essential for clearance of damaged mitochondria^38^, and EndoB1 has been shown to directly bind to UVRAG and, in turn, recruit the autophagic machinery^156,157^. The responsiveness of these proteins to DAG essentially defines a mechanistic link for how the OMM lipid composition can simultaneously recruit multiple membrane-shaping proteins to facilitate organellar fission and clearance via mitophagy. Given the fact that alterations to mitochondrial remodeling are associated with numerous pathological conditions in humans^3,4,158^, including significant contributions to debilitating neurological disorders^159–161^, understanding the regulation of OMM lipid metabolism and transport may offer insights into novel therapeutic strategies that can modulate Drp1-dependent mitochondrial fission. More generally, our results highlight the central importance of membrane lipid composition for coordinating the activities of membrane-shaping proteins, which ultimately function in concert to control organelle dynamics.

**Limitations of the Study:** Our results rely on live-cell experiments that use an induced-proximity system to selectively remodel the lipid composition of the OMM as the result of localized PI hydrolysis to acutely generate DAG. Rapid recruitment of the catalytically active FKBP-*Bc*PI-PLC^3A^ may produce DAG at levels that exceed the physiological range for the OMM. However, the fact that the DAG-induced mitochondrial tubulation and fission are not associated with generic damage to the OMM integrity, and our findings in vitro using membrane templates showing prominent effects at moderate (~ 5%) DAG levels on Drp1 and EndoB1 activities, make us confident that the observed effects related to acute DAG production are relevant to mitochondrial physiology and pathology. Furthermore, it is possible that DAG exerts its influence on Drp1 and EndoB1 functions not locally but by changing bulk membrane properties, including a significant alteration to lipid packing in the OMM. We also stress that the exact physiological setting in which DAG exerts its effect on OMM remodeling remains to be determined, with Lipins identified as likely regulators of DAG content in the OMM. However, this proposed role for Lipins does not preclude the involvement of other lipid metabolic or transport machineries in the regulation of DAG levels within the OMM, and it is likely that numerous mechanisms function in concert to dynamically balance mitochondrial lipid homeostasis. Despite these limitations, our studies directly demonstrate that acute changes in OMM lipid composition, including the specific localized production of DAG, can directly engage essential membrane-shaping proteins to profoundly alter mitochondrial morphology and promote the fission process.

## Methods

### Cell Culture

HEK293A (Invitrogen), COS-7 (CRL-1651; ATCC), HeLa (CCL-2; ATCC), or Drp1^KO^ HeLa^38^ cell lines were cultured in Dulbecco’s Modified Eagle Medium (DMEM-high glucose; Gibco) containing 10% (vol/vol) FBS and supplemented with a 1% solution of penicillin/streptomycin (Gibco). Each of these cell lines were maintained at 37 °C and 5% CO_2_ in a humidified atmosphere. Cell lines were regularly tested for *Mycoplasma* contamination using a commercially available detection kit (InvivoGen). After thawing, cell cultures are also treated with plasmocin (InvivoGen) at 500 µg/mL for the initial three passages (6–9 days) as well as supplemented with 5 µg/mL of the prophylactic for all subsequent passages.

### Reagents

All compounds were prepared in the indicated solvent and stored in small aliquots (10–25 μL) at − 20°C. Rapamycin (Sigma Millipore; 100 μM stock) was dissolved in DMSO, and stock solutions were prepared at 100 μM in DMSO. Carbonylcyanide 4-(trifluoromethoxy)phenylhydrazone (FCCP; Sigma Millipore) and Coelenterazine h (Regis Technologies) were dissolved in 100% ethanol (vol/vol) at 5 mM. Angiotensin II (Human octapeptide; Bachem) was first dissolved in ethanol at 1 mM before being prepared as 100 μM aliquots for storage by dilution with ddH_2_O water. MitoTracker^162^ Red (ThermoFisher Scientific) and tetramethylrhodamine methyl ester perchlorate^65^ (TMRM; Sigma Millipore) were pre-diluted 1:100 in DMSO from the concentrated stock solutions for storage in small aliquots at − 20 °C. Diluted solutions of MitoTracker Red (1:100,000 final concentration) or TMRM (25 nM) were added directly to the medium of transfected cells at a 1:1000 dilution and allowed to equilibrate for 15–30 min at 37 °C prior to imaging.

### DNA Constructs for mammalian expression

Plasmids were constructed by standard restriction cloning using enzymes from New England Biolabs, while site-directed mutagenesis was done using the QuikChange II kit (Agilent). Complex reconfigurations of vector backbones and all point mutations were verified using standard Sanger sequencing (Psomagen, USA). The design of the following plasmids have been described elsewhere: OMM-FRB-ECFP^63^, NES-mEGFP-*Mm*PKD^C1a,b^ (C1a and C1b domains of *Mus musculus* protein kinase D, Residues 134–343)^64^, mRFP-FKBP-5-ptase-dom^163^, ACDB3-FKBP-mRFP^164^, and pcDNA3.1-EndophilinB1 (*Homo sapiens*)^112^. Assembly of mRFP-FKBP-*Bc*PI-PLC^3A^, mRFP-FKBP-*Bc*PI-PLC^DEAD^, iRFP-FKBP-*Bc*PI-PLC^3A^, iRFP-FKBP-*Bc*PI-PLC^DEAD^, OMM-FRB-ECFP^W66A^, AKAP1^TM^-mVenus-T2A-sLuc-NES-*Mm*PKD^C1a,b^, and AKAP1^TM^-FRB-mVenus-T2A-sLuc-FKBP-*Bc*PI-PLC^DEAD^ are all described in Pemberton, et al., 2020^61^. We also thank the laboratories of Dorus Gadella (pmScarlet-I-C1; Addgene Plasmid #85044)^165^, Masamitsu Iino (pCMV-CEPIA2mt; Addgene Plasmid #58218)^67^, Christien Merrifield (Dnm2 (*Homo sapiens*, Isoform 2)-mCherry; Addgene Plasmid #27689)^166^, Yasushi Okada (pN1-TOMM20-mNG; Addgene Plasmid #27689), Isei Tanida (pmNeonGreen(NG)^HO^ (Human Optimized)-G-C1; Addgene Plasmid #127912)^167^, Vladislav Verkhusha (pemiRFP670-N1; Addgene Plasmid #136556)^168^, and Gia Voeltz (mCherry-Drp1 (*Homo sapiens*, Isoform 3); Addgene Plasmid #49152)^141^ for generously providing plasmids. Alternatively, the cloning procedures used for generating the DNA constructs unique to this study are provided below, and the primers required for both PCR-mediated cloning or site-directed mutagenesis are listed in Supplementary Table 1. For both the construct design and any comparative analyses, protein sequences were aligned and visualized using the ENDscript server^169^.

Based on conservation with membrane-oriented residues previously identified in the high-resolution structure of a DAG-bound C1 domain^170^, sequential mutagenesis of the parent NES-mEGFP-*Mm*PKD^C1a,b^ backbone was done to introduce alanine substitutions at hydrophobic residues Phe157 (F157A), Trp166 (W166A), Val289 (V289A), and Phe157 (F301A) to generate the C1a (F157A / W166A) and combined C1a / C1b (F157A / W166A / V289A / F301A; 4 A Mutant) domain mutants. OMM-FRB-mRFP and OMM-FRB-mNG were made from OMM-FRB-ECFP by replacing ECFP with the fluorescent proteins from either pmRFP-C1 (Clonetech) or TOMM20-mNG, respectively, using AgeI and NotI restriction sites. To capitalize on significant recent improvements to near-infrared fluorescent proteins for multiplexed imaging, we made emiRFP670-FKBP-*Bc*PI-PLC^3A^ by replacing the existing mRFP module in mRFP-FKBP-*Bc*PI-PLC^3A^ with emiRFP670 amplified from pemiRFP670-N1 using NheI and BglII restriction sites. mEGFP-Drp1 and mNG^HO^-Drp1 were generated by moving the Drp1 (*Homo sapiens*, Isoform 3) coding sequence from mCherry-Drp1 into either the pmEGFP-C1 (Clonetech) or pmNG^HO^-G-C1 empty vectors, respectively, using BglII and BamHI restriction sites. GTPase-deficient mutations, K38A and T59A, were subsequently introduced into both pmCherry-Drp1 and mNG^HO^-Drp1 by site-directed mutagenesis. Dnm2-mEGFP was made by replacing mCherry in Dnm2 (*Homo sapiens*, Isoform 2)-mCherry with mEGFP derived from pmEGFP-N1 (Clonetech) using KpnI and NotI restriction sites. mNG^HO^-Dnm2 was generated by amplifying the Dnm2 (*Homo sapiens*, Isoform 2) coding sequence from Dnm2-mCherry and inserting this into the pmNG^HO^-G-C1 empty vector using BglII and KpnI restriction sites. The GTPase-deficient K44A mutant was introduced into mNG^HO^-Dnm2 by site-directed mutagenesis.

EndoB1 (Full-Length, Residues 1–365)-mEGFP and EndoB1^N-BAR^ (Residues 1–270)-mEGFP were made by amplifying the corresponding inserts from pcDNA3.1-EndophilinB1 (*Homo sapiens*) and inserting these into the pmEGFP-N1 (Clonetech) empty vector using EcoRI and AgeI restriction sites. An identical cloning strategy was used to generate EndoB2 (Full-Length, Residues 1–394)-mEGFP and EndoB2^N-BAR^ (Residues 1–292)-mEGFP using the MGC Fully Sequenced Human (Horizon Discovery) SH3GLB2 cDNA (Clone ID: 3677306, pOTB7 vector background) as the template for the PCR. EndoB2-mScarlet-I was made from EndoB2-mEGFP by replacing the mEGFP module with mScarlet-I from pmScarlet-I-C1 using Agel and BsrGI restriction sites. To generate EndoB1 mutants lacking the N-terminal amphipathic segments, we synthesized a short DNA fragment (GeneArt^TM^ Custom Gene Synthesis, ThermoFisher Scientific; pcDNA3.1 vector background) comprising residues 1–84 of EndoB1 with specific deletions of either the H0 (^ΔH0^; ΔResidues 5–30) or H1i helices (^ΔH1i^; ΔResidues 69–88). A common EcoRI site that is upstream of the start codon and unique restriction sites that sit between the H0 and H1i helices (PflMI) as well as after both helices (XcmI) were used to create the EndoB1^ΔH0^-mEGFP (EcoRI / PflMI double-digest), EndoB1^ΔH1i^-mEGFP (PflMI / XcmI double-digest), and EndoB1^△H0, △H1i^-mEGFP (EcoRI / XcmI double-digest) mutants. A conserved leucine residue in the dimer interface was changed to aspartate (L227D) in EndoB1-mEGFP by site-directed mutagenesis. For rerouting studies, the extended C-terminus (^CT^) of EndoA1 (Residues 251–352; GenScript SH3GL2 ORF, pcDNA3.1 vector background; Clone ID: OHu21985, Accession: NM_003026.5) or EndoB1 (Residues 269-365) were tagged with FKBP by amplifying the respective sequences and inserting them in place of the Type IV 5-phosphatase domain in mRFP-FKBP-5-ptase using either a HindIII / KpnI (EndoA1^CT^) or SacI / KpnI (EndoB1^CT^) double-digest. Alternatively, the full-length EndoB1-FKBP-mRFP or EndoB1L^227D^-FKBP-mRFP constructs were made by replacing the ACDB3 module in ACDB3-FKBP-mRFP with amplified inserts from the corresponding mEGFP-tagged template using NheI and EcoRI restriction sites.

Lastly, to monitor the relative translocation of EndoB1 variants to the OMM at the level of the cell population, BRET constructs were designed to monitor the resonance energy transfer between sLuc-tagged EndoB1 and an OMM-targeted mVenus. These constructs were built in sequential steps. In the first step, EndoB1-sLuc was made by amplifying sLuc from the AKAP1^TM^-mVenus-T2A-sLuc-NES-*Mm*PKD^C1a,b^ biosensor and inserting it in place of EGFP in EndoB1-mEGFP using PvuI and BsrGI restriction sites. The entire EndoB1-sLuc segment was then inserted into AKAP1^TM^-mVenus-T2A-sLuc-NES-*Mm*PKD^C1a,b^ in place of the DAG sensor using AgeI and KpnI restriction sites to create AKAP1^TM^-mVenus-T2A-EndoB1-sLuc. To further enhance the efficiency of the splitting at the viral 2A peptide sequence, the single T2A site was exchanged for a tandem tPT2A^171^, which was amplified from a short synthetic DNA fragment (Integrated DNA Technologies; gBlocks^TM^), using SalI and AgeI restriction sites. An added NotI site in the flexible linker region between the EndoB1 and sLuc modules was then used together with the upstream AgeI site that follows the tPT2A segment to exchange the full-length EndoB1 with the specified variants amplified from the corresponding mEGFP-tagged templates. These steps ultimately generated the AKAP1^TM^-mVenus-tPT2A-EndoB1-sLuc, AKAP1^TM^-mVenus-tPT2A-EndoB1^N-BAR^-sLuc, AKAP1^TM^-mVenus-tPT2A-EndoB1^△H0^-sLuc, AKAP1^TM^-mVenus-tPT2A-EndoB1^△H1i^-sLuc, AKAP1^TM^-mVenus-tPT2A-EndoB1^△H0,△H1i^-sLuc, and AKAP1^TM^-mVenus-tPT2A-EndoB1^L227D^-sLuc BRET sensors.

### Live-cell confocal microscopy

For imaging studies, HEK293A cells (2.5 × 10^5^ cells/dish) were plated with a final volume of 1.5 mL on 29 mm circular glass-bottom culture dishes (#1.5; Cellvis) pre-coated with 0.01% poly-L-lysine solution (Sigma), while the COS-7 (1.5 × 10^5^ cells/dish), HeLa (3 × 10^5^ cells/dish), and Drp1^KO^ HeLa (3 × 10^5^ cells/dish) cell lines were plated without any additional coating of the culture dishes. The cells were allowed to attach overnight prior to transfection with plasmid DNAs (0.1–0.2 μg/well) using Lipofectamine 2000 (2–5 μL/well; Invitrogen) within a small volume of Opti-MEM (200 μL; Invitrogen) according to the manufacturer’s instructions but with the slight modification of removing the media containing the Lipofectamine-complexed DNA at 4–6 hrs post-transfection and replacing it with complete DMEM. In general, cell densities were always kept between 50–80% confluence for the day of imaging. Also, please note that studies using the rapamycin-inducible protein heterodimerization system used a 1:2:1 ratio of plasmid DNA for transfection of the FKBP-tagged enzyme (0.1 μg), FRB-labeled recruiter (0.2 μg), and indicated protein or biosensor of interest (0.1 μg; total DNA: 0.4 μg/well). After 18–20 hrs of transfection, cells were incubated in 1 mL of modified Krebs-Ringer solution (containing 120 mM NaCl, 4.7 mM KCl, 2 mM CaCl_2_, 0.7 mM MgSO_4_, 10 mM glucose, 10 mM HEPES, and adjusted to pH 7.4) and images were acquired at room temperature using a Zeiss LSM 880 (63x/1.40 N.A. Plan-Apochromat Oil DIC M27 Objective) laser-scanning confocal microscopes (Carl Zeiss Microscopy). Image acquisition was performed using the ZEN software system (Carl Zeiss Microscopy), while image preparation and analysis was done using the open-source FIJI platform^172^.

### Measurements using BRET-based biosensors within intact cells

The construction of the BRET-based lipid reporters has been detailed above. Briefly, the design of these biosensors allows for the quantitative measurement of membrane lipid composition or EndoB enrichment within defined subcellular compartments at the population scale using intact cells. This methodology relies on a plasmid design that incorporates a viral 2 A peptide sequence to facilitate the production of two separate proteins in transfected cells at a fixed stoichiometry; specifically, a membrane-anchored BRET acceptor (mVenus) and a Luciferase-tagged protein of interest that serves as the BRET donor. BRET measurements were made at 37 °C using a Tristar2 LB 942 Multimode Microplate Reader (Berthold Technologies) with customized emission filters (540/40 nm and 475/20 nm). HEK293A cells (0.75 × 10^5^ cells/well) were seeded in a 200 μL total volume to white-bottom 96 well plates pre-coated with 0.01% poly-L-lysine solution (Sigma) and cultured overnight. Cells were then transfected with 0.35 μg of the specified BRET biosensor using Lipofectamine 2000 (1 μL/well) within OPTI-MEM (40 μL) according to the manufacturer’s protocol, once again with the slight modification of removing the media containing the Lipofectamine-complexed DNA and replacing it with complete culture medium at between 4–6 hrs post-transfection. Where indicated, additional plasmids, including components of the rapamycin-inducible heterodimerization system, were transfected together with the BRET biosensor at an empirically determined ratio of 1:1:5 for the FRB (0.05 μg), FKBP (0.05 μg), and BRET (0.35 μg) constructs, respectively (0.45 μg/well total). Between 20–24 hrs post-transfection, the cells were quickly washed before being incubated for 30 mins in 50 µL of modified Krebs-Ringer buffer (containing 120 mM NaCl, 4.7 mM KCl, 2 mM CaCl_2_, 0.7 mM MgSO_4_, 10 mM glucose, 10 mM HEPES, and adjusted to pH 7.4) at 37 °C in a CO_2_-independent incubator. After the pre-incubation period, the cell-permeable luciferase substrate, coelenterazine h (40 µL, final concentration 5 µM), was added and the signal from the mVenus fluorescence and sLuc luminescence were recorded using 485 and 530 nm emission filters over a 4 min baseline BRET measurement (15 sec / cycle). Following the baseline recordings, where indicated, the plates were quickly unloaded for the addition of various treatments, which were prepared in a 10 µL volume of the modified Krebs-Ringer solution and added manually. Detection time was always 500 ms for each wavelength and measurements were initiated at the 0 min demarcation indicated on all graphs and continued for 60 min (15 sec / cycle) after the addition of any treatments. All measurements were carried out in triplicate wells and repeated in three independent experiments. From each well, the BRET ratio was calculated by dividing the 530 nm and 485 nm intensities, which were then normalized to the baseline measurement. To facilitate the pooling of data from individual wells and between replicate experiments, the raw BRET ratios were processed by using a simple moving average with a five-cycle interval across the BRET kinetic. The processed BRET ratios obtained from drug-treated wells were then normalized to internal vehicle-treated controls. In addition, where indicated, values obtained for the same BRET biosensor from experiments using multiple recruitable enzymes or different FRB-tagged recruiters were also normalized to control measurements made simultaneously using the corresponding catalytically-inactive enzyme variants.

### Protein expression and purification

Drp1 (*Homo sapiens*, Isoform 3)-StrepII and Drp1 (*Homo sapiens*, Isoform 3)-EGFP-StrepII were cloned in pET15B^79^. The assembly of pHGT-2-6xHis-*Bc*PI-PLC and pHGT-2-6xHis-*Bc*PI-PLC^H32A^ was described in Pemberton, et al., 2020^61^. 6xHis-mEGFP-*Mm*PKD^C1a^ (C1a domain (^C1a^) of *Mus musculus* (*Mm*) protein kinase D (PKD), Residues 134–198), 6xHis-EndoB1 (Residues 1–365)-mEGFP, 6xHis-EndoB1^N-BAR^ (residues 1–270)-mEGFP, and 6xHis-EndoB2^N-BAR^ (Residues 1–292)-mEGFP were also all cloned into the pHGT-2 vector^173^ using BamHI and NotI restriction sites (primers listed in Supplementary Table 2). Briefly, the pHGT-2 plasmid is derived from the pRSFDuet-1 backbone (Novagen) but also contains an N-terminal 6xHis-GB1 solubility tag along with a tobacco etch virus (TEV) cleavage site. Constructs containing the Drp1 and *Bc*PI-PLC variants were expressed in NiCo21(DE3) cells grown under autoinduction conditions for 36 hrs at 18 ^°^C. EndoB constructs were expressed in T7 Express Competent *E. coli*. Cells were grown to an optical density (OD) of 0.4. Expression was induced with 0.1 mM isopropyl-D-1-thiogalactopyranoside (IPTG) and grown overnight at 18 ^°^C. Cells were pelleted at 6000 × *g* and stored at − 40 ^°^C. Pellets were thawed in lysis buffer (20 mM HEPES, pH 7.4, 500 mM NaCl, and 1 mM phenylmethylsulfonyl fluoride) and lysed by sonication in an ice-water bath. For the purification of 6xHis-tagged proteins, the lysis buffer additionally contained imidazole (10 mM). Lysates were spun at 30,000 × *g* for 30 min, and the supernatant was loaded onto either HiTrap Strep-Tactin column (5 mL; GE Lifesciences) for enrichment of StrepII-tagged constructs or HiTrap Chelating HP column (5 mL; GE Lifesciences) for enrichment of 6xHis tagged constructs. The columns were washed extensively with lysis buffer and then exchanged for assay buffer (20 mM HEPES, pH 7.4, 150 mM NaCl). For the Drp1-expressing constructs, the column was additionally washed with assay buffer containing 100 mM EDTA to remove any bound nucleotide. StrepII-tagged proteins were eluted in assay buffer containing 2.5 mM desthiobiotin. 6xHis-tagged proteins were eluted with 20 mM HEPES buffer (pH 7.4) with 150 mM NaCl against an imidazole gradient of 10–300 mM for the EndoB proteins and 10–500 mM for others. Eluates were run on a gel and fractions containing pure proteins were pooled and kept at 4 ^°^C. Drp1 was spun at 100,000 × *g* for 30 mins to remove any aggregates before use in assays. StrepII-MFF(ΔTMD)-6xHis was purified as described earlier^79^. Briefly, bacterial pellets were lysed, and the StrepII-MFF(ΔTMD)-6xHis construct was enriched through sequential purifications using a HiTrap Chelating HP column followed by a HiTrap Strep-Tactin column.

### Preparation of supported membrane templates (SMrTs)

Dioleoyl-phosphatidylcholine (DOPC) (Avanti Polar Lipids), bovine heart cardiolipin (CL; Avanti Polar Lipids), dioleoylglycerol (DAG; Avanti Polar Lipids), liver phosphatidylinositol (PI; Avanti Polar Lipids) and Texas Red™ 1,2-dihexadecanoyl-*sn*-glycero-3-phosphoethanolamine (TR-DHPE; Invitrogen) were aliquoted from their stock solutions and reconstituted at the desired molar ratio using chloroform to a final total lipid concentration of 1 mM. For experiments using the MFF adapter, 1,2-dioleoyl-sn-glycero-3-[(N-(5-amino-1-carboxypentyl)iminodiacetic acid)succinyl] (nickel salt, DGS-NTA(Ni^2+^); Avanti Polar Lipids) was added at a concentration of 5% within the lipid mixture. Supported membrane templates (SMrTs) were formed as described previously^87^. Briefly, 2 µL of the indicated lipid mix was spotted on a glass coverslip, which were covalently passivated with PEG400 or PEG8000. Lipids were spread on the coverslip, allowed to dry, and then assembled within an FCS2 flow cell (Bioptechs). The chamber was hydrated with 20 mM HEPES buffer (pH 7.4) with 150 mM NaCl, and the flow of buffer at high rates extruded the reservoir into an array of membrane tubes. The region where the lipid was initially spread formed planar bilayers. Tube sizes were estimated based on a previously described in situ calibration procedure^87^. Briefly, we first estimated the integrated fluorescence per unit area of the membrane using the planar bilayer for reference. Using this value, we converted the integrated fluorescence of a tube of defined length to its radius by assuming it to be a cylinder.

### SMrTs Assays

Proteins were spun at 100,000 × *g* for 30 mins to remove any aggregates before use in assays. Experiments were carried out in 20 mM HEPES buffer (pH 7.4) with 150 mM NaCl, and the temperature was maintained at 25 °C. Unless stated otherwise, protein concentrations were estimated from their theoretical molar extinction coefficients and absorbance measured at the 280 nm wavelength. For detecting DAG, templates were incubated with mEGFP-*Mm*PKD^C1a^ (10 µM) for 10 min prior to washing off excess protein before imaging. For lipid conversion studies, templates were incubated with *Bc*PI-PLC (1 µM) or the inactive *Bc*PI-PLC^H32A^ mutant (1 µM) for 30 mins, and excess protein was washed off before flowing in mEGFP-*Mm*PKD^C1a^ (10 µM) and imaging. For membrane fission assays, templates were incubated with Drp1 (1 µM) in assay buffer containing GTP (1 mM; Jena Bioscience) and MgCl_2_ (1 mM) for 10 or 30 mins before imaging. For Drp1 binding assays, templates were incubated with a mixture of Drp1-mEGFP (0.5 µM) and untagged Drp1 (0.5 µM) in assay buffer containing GppNHp (1 mM; Jena Bioscience) and MgCl_2_ (1 mM) for 10 mins and excess protein was washed off before imaging. For experiments on organelle mimics with membrane-anchored MFF, DGS-NTA(Ni^2+^)-containing templates were incubated with MFF (1 µM) for 10 mins, and excess protein was washed off before flowing in Drp1. Real-time analyses of Drp1-catalyzed fission reactions are acutely sensitive to the light-induced protein oxidation that can occur during continuous fluorescence imaging^174^. Therefore, for measurements of the fission time kinetics, templates were first incubated with Drp1 (1 µM) in assay buffer containing GppNHp (1 mM) and MgCl_2_ (1 mM) for 10 mins, followed by washing off excess protein with a buffer containing an oxygen scavenger cocktail (OSC) and DTT (1 mM)^87,174^. Templates were then imaged while flowing buffer containing the OSC and DTT mixed together with GTP (1 mM) and MgCl_2_ (1 mM). EndoB1 protein concentrations were estimated using the extinction coefficient of EGFP. For analyzing membrane tubulation, proteins (1 µM) were flowed onto templates incubated for 10 mins before washing of excess protein and imaging. For real-time analysis of EndoB1^N-BAR^-EGFP mediated membrane tubulation, templates were equilibrated with OSC and DTT. Thereafter, planar bilayers were imaged while flowing in EndoB1^N-BAR^-EGFP (1 µM) in a buffer containing OSC with DTT. To analyze the sizes of the EndoB1^N-BAR^-EGFP-coated tubes, line profiles across the tubes before coalescence or coiling were acquired, and this intensity was converted to a measurement of the tube size according to methods described previously^87^, and explained above. For analyzing the effects of Drp1 on EndoB1 tubules, templates were equilibrated with buffer containing OSC and DTT and imaged while flowing in EndoB1^N-BAR^-EGFP (0.1 µM) mixed with Drp1 (1 µM), GTP (1 mM) and MgCl_2_ (1 mM) in buffer containing OSC and DTT. Photobleaching experiments were carried out using widefield fluorescence microscopy. Templates were incubated with EndoB1^N-BAR^-EGFP (0.1 µM) mixed with Drp1 (1 µM) in buffer containing GTP (1 mM) and MgCl_2_ (1 mM) for 1 hr. Excess protein was washed off and the fluorescent TR-DHPE lipids in the planar bilayer were bleached by increasing the incident light intensity. The light intensity was then lowered, and the bleached region was imaged at 10 sec intervals to monitor the recovery of the fluorescence signal.

### Fluorescence microscopy of SMrTs and statistical analyses

Fluorescence imaging of SMrTs was carried out on an Olympus IX83 inverted fluorescence microscope attached to a stable LED light source (CoolLED) and an Evolve 512 EMCCD camera (Photometrics). Image acquisition was controlled by Micro-Manager^175^ and rendered using Fiji^172^. Statistical analyses were carried out on GraphPad Prism 9 (GraphPad Software Company).

### Reporting summary

Further information on research design is available in the Nature Portfolio Reporting Summary linked to this article.

## Supplementary information

Supplementary Information

Peer Review file

Description of Addtional Supplementary Files

Supplementary Movie 1

Supplementary Movie 2

Supplementary Movie 3

Supplementary Movie 4

Supplementary Movie 5

Supplementary Movie 6

Supplementary Movie 7

Supplementary Movie 8

Supplementary Movie 9

Supplementary Movie 10

Supplementary Movie 11

Supplementary Movie 12

Supplementary Movie 13

Reporting Summary

## Source data

Source Data

## Data Availability

Data supporting the findings of this study are available from the corresponding authors upon reasonable request. Source data for each of the graphs presented are provided in this paper and stored in files created using Microsoft® Excel for Mac (Version 16.78.3; Microsoft Corporation) and GraphPad Prism 9 (GraphPad Software Company). Source data are provided in this paper.
