# Supplementary material for: Acute diacylglycerol production activates critical membrane-shaping proteins leading to mitochondrial tubulation and fission
Source: Nat Commun. 2025 Mar 19;16:2685. doi: 10.1038/s41467-025-57439-9 (PMC11920102; doi:10.1038/s41467-025-57439-9)
Supplement: Supplementary file 1 — Supplementary Information [file 41467_2025_57439_MOESM1_ESM.pdf]

Supplementary Information

Compiled for:

Acute Diacylglycerol Production Activates Critical Membrane-Shaping Proteins Leading to Mitochondrial Tubulation and Fission

Joshua G. Pemberton[#,*], Krishnendu Roy[#], Yeun Ju Kim, Tara D. Fischer, Vijay Joshi, Elizabeth Ferrer, Richard J. Youle, Thomas J. Pucadyil*, and Tamas Balla*.

[#]These authors contributed equally; *Corresponding authors.

Contents:

Page 1,2: Supplementary Figure 1 and Legend.
Page 3,4: Supplementary Figure 2 and Legend.
Page 5,6: Supplementary Figure 3 and Legend.
Page 7,8: Supplementary Figure 4 and Legend.
Page 9,10: Supplementary Figure 5 and Legend.
Page 11,12: Supplementary Figure 6 and Legend.
Page 13,14: Supplementary Figure 7 and Legend.
Page 15,16: Supplementary Figure 8 and Legend.
Page 17,18: Supplementary Figure 9 and Legend.
Page 19,20: Supplementary Figure 10 and Legend.
Page 21,22: Supplementary Figure 11 and Legend.
Page 23,24: Supplementary Figure 12 and Legend.
Page 25,26: Supplementary Table 1.
Page 27: Supplementary Table 2.

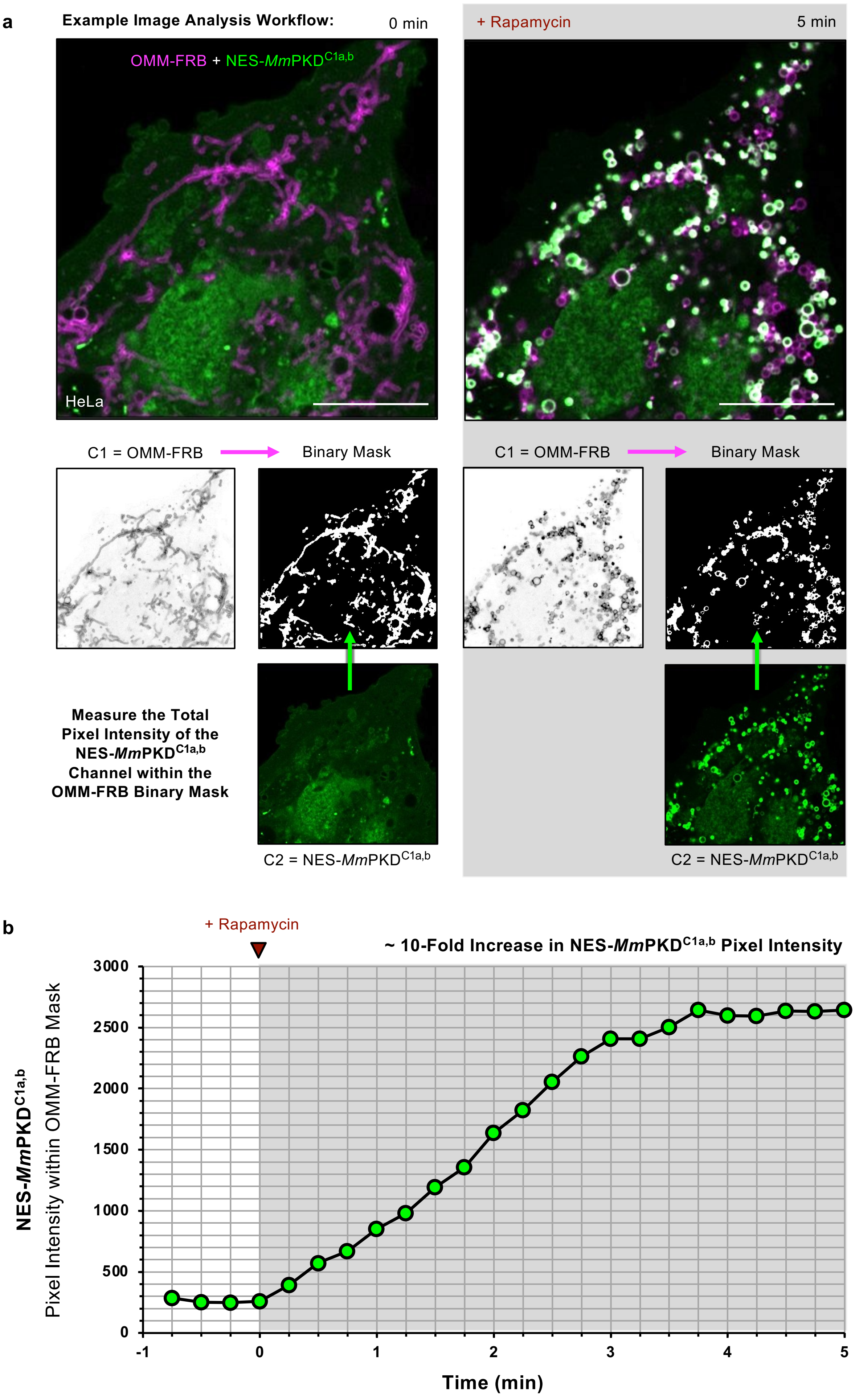
a
Example Image Analysis Workflow:
0 min
+ Rapamycin
5 min
OMM-FRB + NES-*Mm*PKD[C1a,b]
HeLa
C1 = OMM-FRB
Binary Mask
C1 = OMM-FRB
Binary Mask
Measure the Total Pixel Intensity of the NES-*Mm*PKD[C1a,b] Channel within the OMM-FRB Binary Mask
C2 = NES-*Mm*PKD[C1a,b]
C2 = NES-*Mm*PKD[C1a,b]
b
+ Rapamycin
~ 10-Fold Increase in NES-*Mm*PKD[C1a,b] Pixel Intensity
NES-*Mm*PKD[C1a,b]
Pixel Intensity within OMM-FRB Mask
3000
2500
2000
1500
1000
500
0
-1
0
1
2
3
4
5
Time (min)

Supplementary Figure 1.

Recruitment of FKBP-*Bc*PI-PLC^{3A} to the mitochondrial network induces rapid and robust translocation of the NES-*Mm*PKD^{C1a,b} sensor to the OMM.

(a) Representative images of a COS-7 cell (5 µm scale bar) present an overlay showing localization of the OMM-targeted FRB recruiter (OMM-FRB-ECFP, Channel 1 (C1), magenta) and the high-affinity DAG-binding probe (NES-mEGFP-*Mm*PKD^{C1a,b}, Channel 2 (C2), green) in response to rapamycin-induced (100 nM) recruitment of the catalytically active mRFP-FKBP-*Bc*PI-PLC^{3A} (*channel not shown*) to the cytosolic membrane leaflet of the mitochondria. For each image in the time series, thresholding of the signal from the OMM-FRB channel was used to create a binary mask that was then used to measure the pixel intensities in the NES-*Mm*PKD^{C1a,b} channel over time. **(b)** Quantified kinetics of the NES-*Mm*PKD^{C1a,b} translocation to the OMM as measured using the single-cell image analysis workflow outlined here.

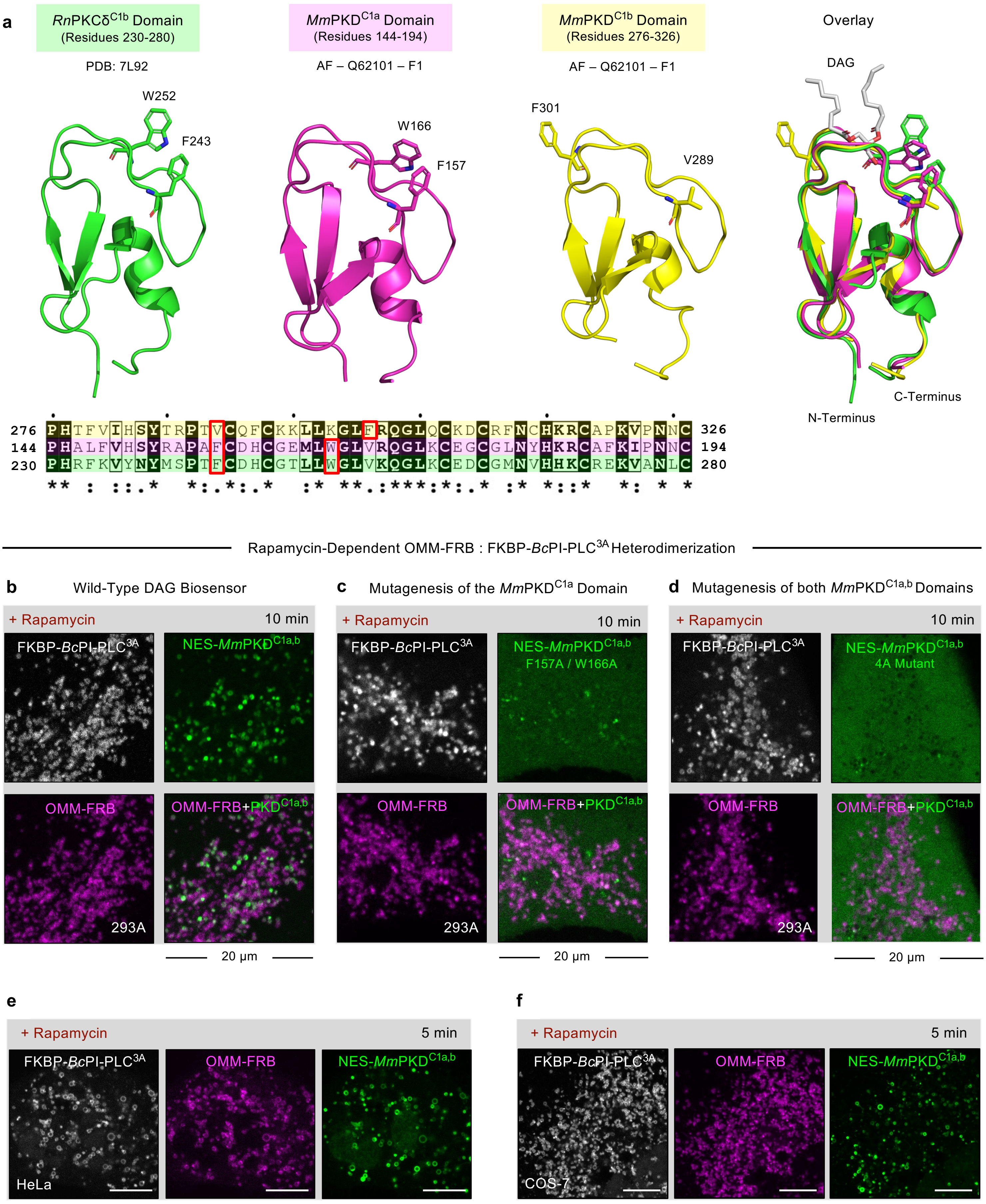
a
RnPKCδC1b Domain (Residues 230-280)
PDB: 7L92
MmPKDC1a Domain (Residues 144-194)
AF – Q62101 – F1
MmPKDC1b Domain (Residues 276-326)
AF – Q62101 – F1
Overlay
W252
F243
W166
F157
F301
V289
DAG
C-Terminus
N-Terminus
276 PHTFVIHSYTRPTVCQFCKKLLKGLFRQGLQCKDCRFNCHKRCAPKVPNNC 326
144 PHALFVHSYRAPAFCDHCGEMLWGLVRQGLKCEGCGLNYHKRCAFKIPNNC 194
230 PHRFKVYNYMSPTFCDHCGTLLWGLVKQGLKCEDCGMNVHHKCREKVANLC 280
Rapamycin-Dependent OMM-FRB : FKBP-BcPI-PLC3A Heterodimerization
b Wild-Type DAG Biosensor
c Mutagenesis of the MmPKDC1a Domain
d Mutagenesis of both MmPKDC1a,b Domains
+ Rapamycin
10 min
FKBP-BcPI-PLC3A
NES-MmPKDC1a,b
F157A / W166A
4A Mutant
OMM-FRB
OMM-FRB+PKDC1a,b
293A
20 μm
e
f
+ Rapamycin
5 min
HeLa
COS-7

Supplementary Figure 2.

Mutation of membrane-oriented hydrophobic residues prevents translocation of the high-affinity DAG biosensor to the OMM in response to local FKBP-*Bc*PI-PLC^{3A} recruitment.

(a) Comparison of the high-resolution structure solved for the DAG-bound C1b domain (Residues 230-280) from *Rattus norvegicus* (*Rn*) protein kinase Cδ (*Rn*PKCδ^{C1b}; PDB Accession: 7L92; green)[176] with AlphaFold2 (DeepMind)[173,174] predictions of the C1a (*Mm*PKD^{C1a}; Residues 144-194; extracted from AF-Q62101-F1, magenta) and C1b (*Mm*PKD^{C1b}; Residues 276-326; extracted from AF-Q62101-F1, yellow) domains from *Mus musculus* protein kinase D (*Mm*PKD). The tandem *Mm*PKD C1a and C1b domains are connected by an endogenous disordered linker, and this isolated segment (*Mm*PKD^{C1a,b}; Residues 134-343) has been characterized as a high-affinity biosensor that is capable of reliably monitoring membrane DAG levels. Sequence alignments (bottom; ESPript3.0)[177] and structural overlays (right) of these C1 domains shows that aromatic residues are involved in forming the membrane-oriented face of the DAG-binding pocket. These and other hydrophobic residues are shown boxed in the sequence alignment (red) and highlighted as stick-representations of the amino acid sidechains within the ribbon diagram generated for each structure. Protein structures were prepared using the PyMOL Molecular Graphics System (Version 3.0; Schrödinger, LLC). **(b-d)** Representative images of HEK293A cells (10 µm scale bar) after treatment with rapamycin (100 nM) for 10 min showing the localization of the OMM-targeted recruiter (OMM-FRB-ECFP, magenta) and catalytically active *Bc*PI-PLC^{3A} enzyme (mRFP-FKBP-*Bc*PI-PLC^{3A}, grey) together with either the wild-type **(b)** NES-mEGFP-*Mm*PKD^{C1a,b} biosensor (green) or variants with mutations to the hydrophobic residues positioned at the membrane-oriented interface of the **(c)** C1a (F157A / W166A) or **(d)** both the C1a and C1b (F157A / W166A / V289A / F301A, 4A Mutant) domains of the probe. **(e, f)** Representative images of HeLa (**e**) or COS-7 (**f**) cells (5 µm scale bars) showing localization of the OMM-targeted FRB recruiter (OMM-FRB-ECFP, magenta) and a high-affinity DAG-binding probe (NES-mEGFP-*Mm*PKD^{C1a,b}, green) after 5 min of rapamycin-induced (100 nM) recruitment of the catalytically active mRFP-FKBP-*Bc*PI-PLC^{3A} enzyme (not shown) to the cytosolic membrane leaflet of the mitochondria. Composite images present an overlay of the OMM-FRB with the DAG biosensor.

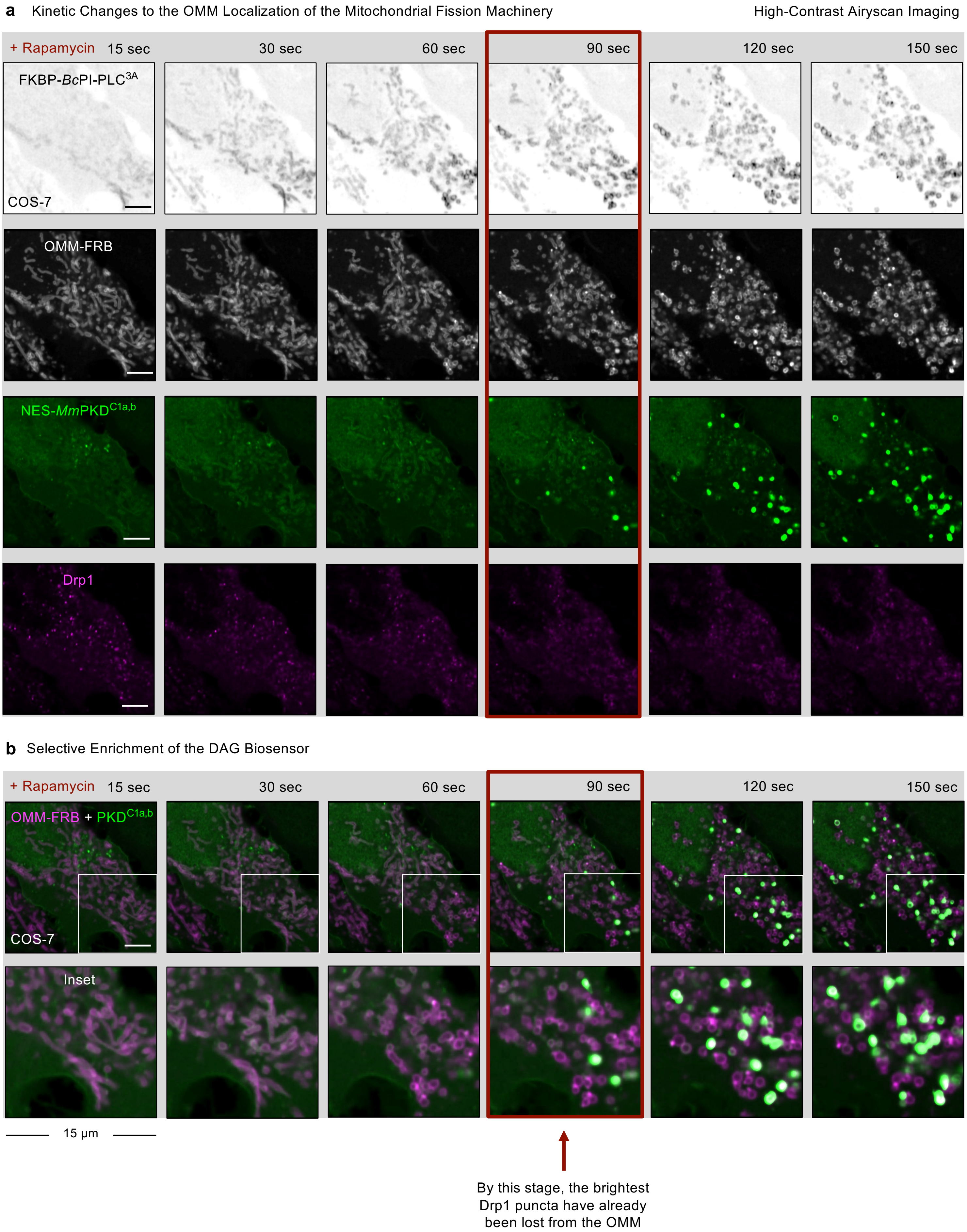
a Kinetic Changes to the OMM Localization of the Mitochondrial Fission Machinery
High-Contrast Airyscan Imaging
+ Rapamycin
15 sec
30 sec
60 sec
90 sec
120 sec
150 sec
FKBP-BcPI-PLC3A
COS-7
OMM-FRB
NES-MmPKDC1a,b
Drp1
b Selective Enrichment of the DAG Biosensor
+ Rapamycin
15 sec
30 sec
60 sec
90 sec
120 sec
150 sec
OMM-FRB + PKDC1a,b
COS-7
Inset
15 μm
By this stage, the brightest
Drp1 puncta have already
been lost from the OMM

Supplementary Figure 3.

Mitochondrial fission induced by recruitment of FKBP-*Bc*PI-PLC^{3A} and acute DAG production is associated with the rapid disassembly of OMM-associated Drp1 puncta.

(a) Representative images of COS-7 cells (5 μm scale bar) showing localization of the OMM-targeted FRB recruiter (OMM-FRB-ECFP, grey), high-affinity DAG biosensor (NES-mEGFP-*Mm*PKD^{C1a,b}, green), and Drp1 (mCherry-Drp1, magenta) in response to rapamycin-induced (100 nM) recruitment of the catalytically active FKBP-*Bc*PI-PLC^{3A} (iRFP-FKBP-*Bc*PI-PLC^{3A}, inverted grey) to the cytosolic membrane leaflet of the mitochondria. The bulk fragmentation response of the mitochondrial network is shown over the 150 sec period immediately after rapamycin treatment, which is the time frame consistently associated with the majority of the OMM fission events. Composite image series presenting an overlay of the OMM-FRB together with the NES-*Mm*PKD^{C1a,b} probe are included in **(b)**, while an overlay of OMM-FRB and Drp1 are presented separately in **Figure 2B**. The complete time series is also provided as **Supplementary Movie 1**.

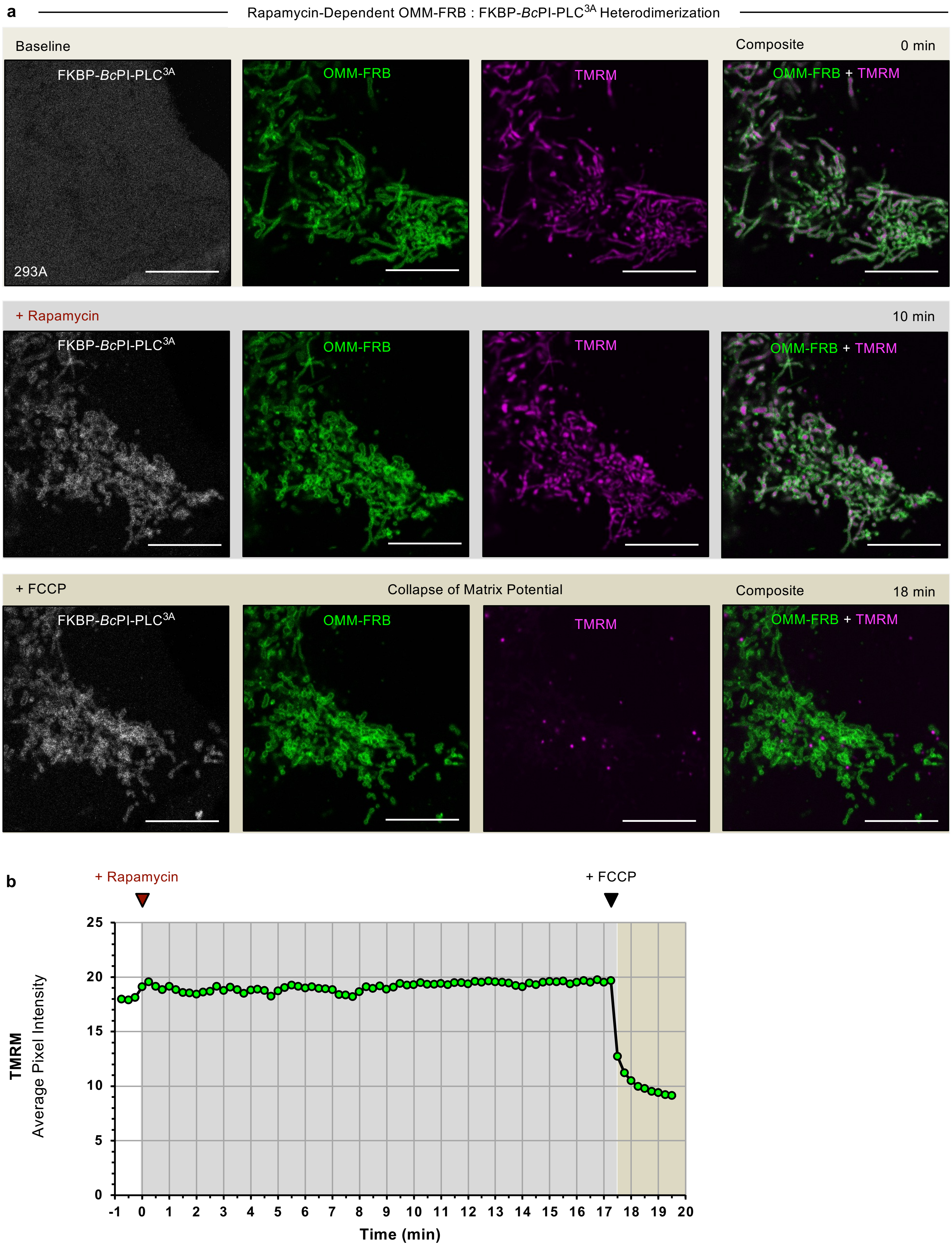
a
Rapamycin-Dependent OMM-FRB : FKBP-BcPI-PLC3A Heterodimerization
Baseline
Composite
0 min
FKBP-BcPI-PLC3A
OMM-FRB
TMRM
OMM-FRB + TMRM
293A
+ Rapamycin
10 min
+ FCCP
Collapse of Matrix Potential
18 min
b
+ Rapamycin
+ FCCP
TMRM
Average Pixel Intensity
Time (min)
25
20
15
10
5
0
-1
0
1
2
3
4
5
6
7
8
9
10
11
12
13
14
15
16
17
18
19
20

Supplementary Figure 4.

Mitochondrial membrane potential is not acutely altered by *Bc*PI-PLC^{3A}-induced mitochondrial fragmentation, and can still be uncoupled by treatment with FCCP.

(a) Representative images of HEK293A cells (10 μm scale bar) stably loaded with the mitochondrial membrane potential-sensitive dye, TMRM (25 nM, magenta), showing localization of the OMM-targeted FRB recruiter (OMM-FRB-ECFP, green) before (left panels), 10 min after (middle panels) rapamycin-induced (100 nM) recruitment of the catalytically active *Bc*PI-PLC^{3A} (mRFP-FKBP-*Bc*PI-PLC^{3A}, grey) to the cytosolic membrane leaflet of the mitochondria (middle panels), as well as immediately after treatment with FCCP (5 μM; right panels). The composite images (bottom row) show an overlay of the OMM-FRB together with the TMRM dye, which remains enriched in the matrix after the *Bc*PI-PLC^{3A}-induced mitochondrial fragmentation but is completely lost after the membrane potential is uncoupled by FCCP. **(b)** Quantified kinetics of the average pixel intensities measured for the TMRM channel.

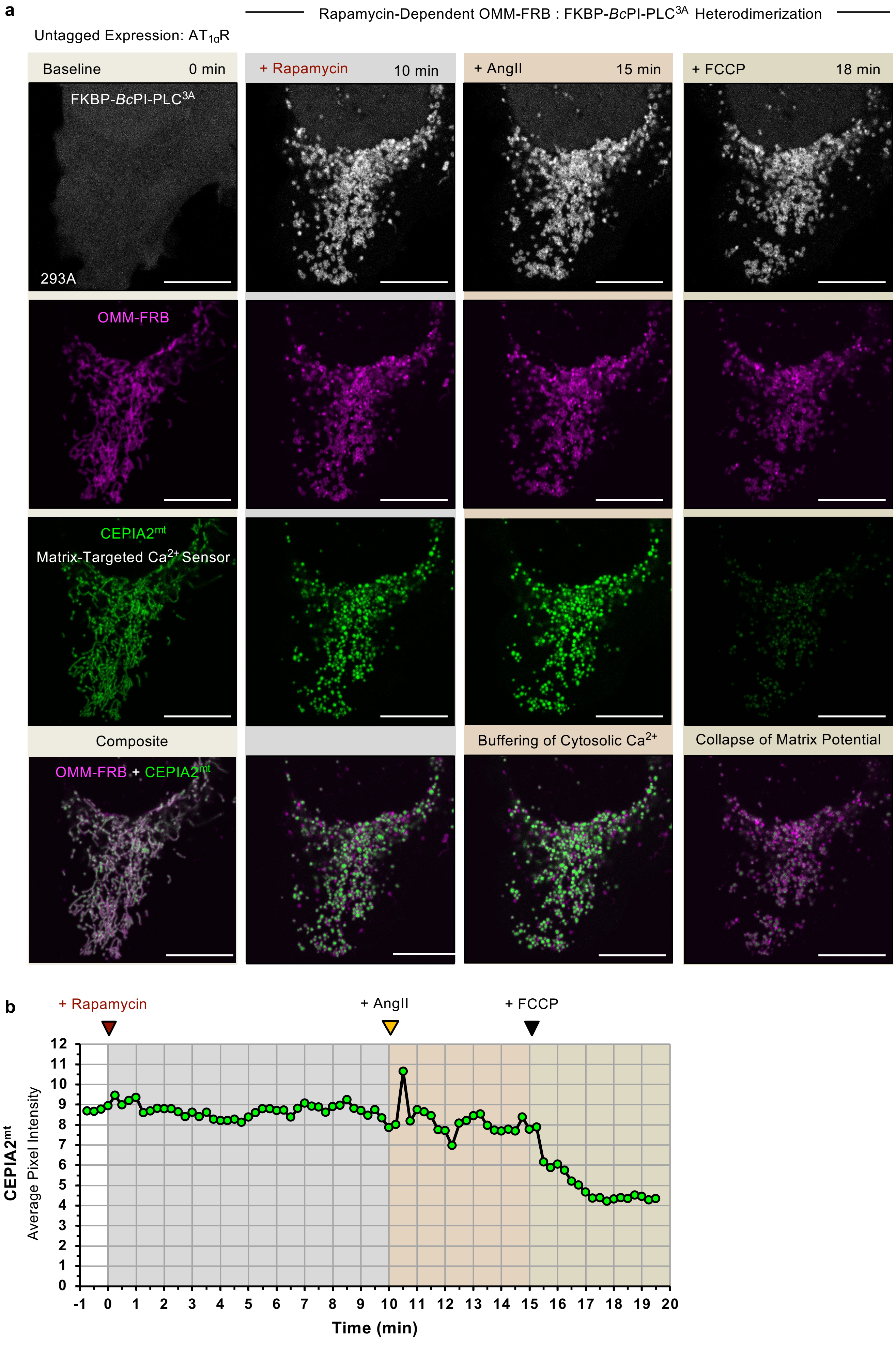
a
Rapamycin-Dependent OMM-FRB : FKBP-BcPI-PLC3A Heterodimerization
Untagged Expression: AT1αR
Baseline 0 min
+ Rapamycin 10 min
+ AngII 15 min
+ FCCP 18 min
FKBP-BcPI-PLC3A
293A
OMM-FRB
CEPIA2mt
Matrix-Targeted Ca2+ Sensor
Composite
Buffering of Cytosolic Ca2+
Collapse of Matrix Potential
OMM-FRB + CEPIA2mt
b
+ Rapamycin
+ AngII
+ FCCP
CEPIA2mt
Average Pixel Intensity
Time (min)

Supplementary Figure 5.

Recruitment of FKBP-*Bc*PI-PLC^{3A} to the OMM does not alter Ca^{2+} levels within the mitochondrial matrix or prevent buffering of increased cytosolic free Ca^{2+} by the mitochondrial network.

(a) Representative images of HEK293A cells (10 µm scale bar) expressing $AT_{1a}R$ (pcDNA3.1-HA-$AT_{1a}R$, *untagged construct not visible for live-cell imaging*) showing localization of the OMM-targeted FRB recruiter (OMM-FRB-ECFP, magenta) and a matrix-targeted Ca^{2+} sensor (CEPIA2-mt, green) before (left panels), 10 min after rapamycin-induced (100 nM) recruitment of the catalytically active *Bc*PI-PLC^{3A} (mRFP-FKBP-*Bc*PI-PLC^{3A}, grey) to the cytosolic membrane leaflet of the mitochondria (middle-left panels), as well as immediately after treatment with the Gq-coupled receptor agonist, angiotensin-II (AngII, 100 nM; middle-right panels), and then FCCP (5 µM; right panels). The composite images (bottom row) show an overlay of the OMM-FRB together with the mitochondrial Ca^{2+} sensor, which shows no apparent change in the relative intensity of the Ca^{2+} signal after the *Bc*PI-PLC^{3A}-induced mitochondrial fragmentation. However, the signal from the Ca^{2+} sensor immediately increases after an acute elevation to the cytosolic free Ca^{2+} levels by stimulation of the $AT_{1a}R$-expressing cells with AngII, which shows that, despite the widespread fragmentation, there is not generic damage to the OMM and that the molecular machinery necessary for Ca^{2+} uptake into the mitochondrial matrix remains functional. Finally, after treatment with FCCP, the signal intensity from the mitochondrial Ca^{2+} sensor is rapidly decreased throughout the entire mitochondrial network. **(b)** Quantified kinetics of the average pixel intensities measured for the CEPIA2-mt channel.

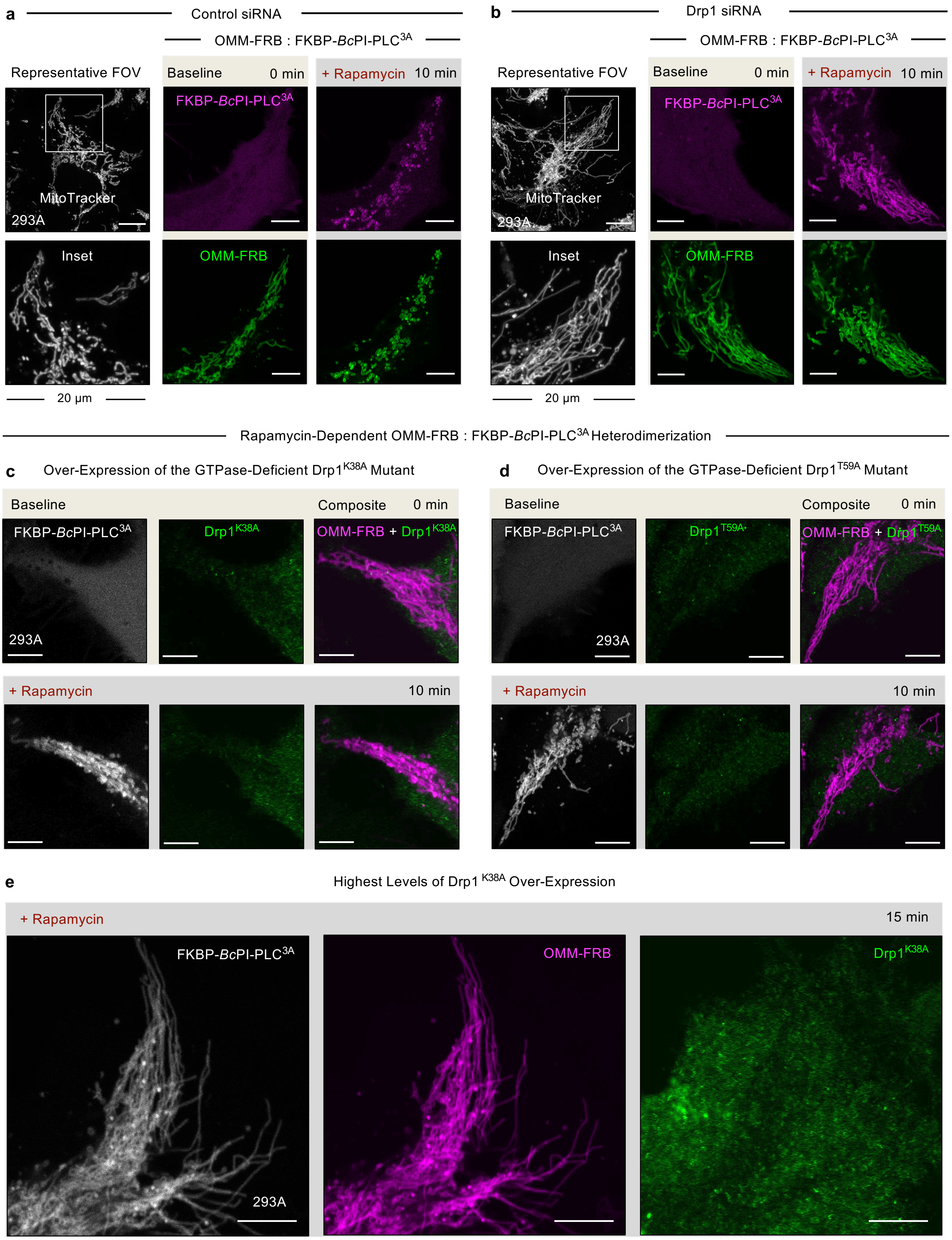
a
Control siRNA
OMM-FRB : FKBP-BcPI-PLC3A
Representative FOV
Baseline 0 min
+ Rapamycin 10 min
FKBP-BcPI-PLC3A
MitoTracker
293A
Inset
OMM-FRB
20 μm
b
Drp1 siRNA
OMM-FRB : FKBP-BcPI-PLC3A
Representative FOV
Baseline 0 min
+ Rapamycin 10 min
FKBP-BcPI-PLC3A
MitoTracker
293A
Inset
OMM-FRB
20 μm
Rapamycin-Dependent OMM-FRB : FKBP-BcPI-PLC3A Heterodimerization
c
Over-Expression of the GTPase-Deficient Drp1K38A Mutant
Baseline
Composite 0 min
FKBP-BcPI-PLC3A
Drp1K38A
OMM-FRB + Drp1K38A
293A
+ Rapamycin 10 min
d
Over-Expression of the GTPase-Deficient Drp1T59A Mutant
Baseline
Composite 0 min
FKBP-BcPI-PLC3A
Drp1T59A
OMM-FRB + Drp1T59A
293A
+ Rapamycin 10 min
e
Highest Levels of Drp1 K38A Over-Expression
+ Rapamycin 15 min
FKBP-BcPI-PLC3A
OMM-FRB
Drp1K38A
293A

Supplementary Figure 6.

***Bc*PI-PLC^{3A}-induced fission of the mitochondrial network requires the catalytic activity of Drp1.**

(a, b) Representative images of HEK293A cells (10 μm scale bar) treated with control siRNA **(a)** or Drp1 siRNA **(b)** and loaded with MitoTracker Red (grey) are presented as maximum intensity projections (control siRNA, 3.4480 μm total depth; Drp1 siRNA, 5.6031 μm total depth) to highlight the significant differences in mitochondrial morphology (left column panels). For both the control and Drp1 siRNA treatment conditions, also shown are representative images of the localization for the OMM-targeted FRB recruiter (OMM-FRB-mNG, green) before (middle column panels) or 10 min after (right column panels) rapamycin-induced (100 nM) recruitment of the catalytically active *Bc*PI-PLC^{3A} (mRFP-FKBP-*Bc*PI-PLC^{3A}, magenta) to the cytosolic membrane leaflet of the mitochondria. **(c, d)** Representative composite images of HEK293A cells (5 μm scale bar) showing the localization of the OMM-targeted FRB recruiter (OMM-FRB-mRFP, magenta) and GTPase-deficient mutants of Drp1 (**c**, mNG^{HO}-Drp1^{K38A}, green; **d**, mNG^{HO}-Drp1^{T59A}, green) before (top row panels) or 10 min after (bottom row panels) rapamycin-induced (100 nM) recruitment of the catalytically active *Bc*PI-PLC^{3A} (emiRFP670-FKBP-*Bc*PI-PLC^{3A}, grey) to the cytosolic membrane leaflet of the mitochondria. Composite images present an overlay of the OMM-FRB together with the mutant variants of Drp1. **(e)** Representative images of the experiment presented in (**c**) are also shown at 15 min post-rapamycin treatment for HEK293A cells (5 μm scale bars) with relatively high levels of Drp1^{K38A} over-expression.

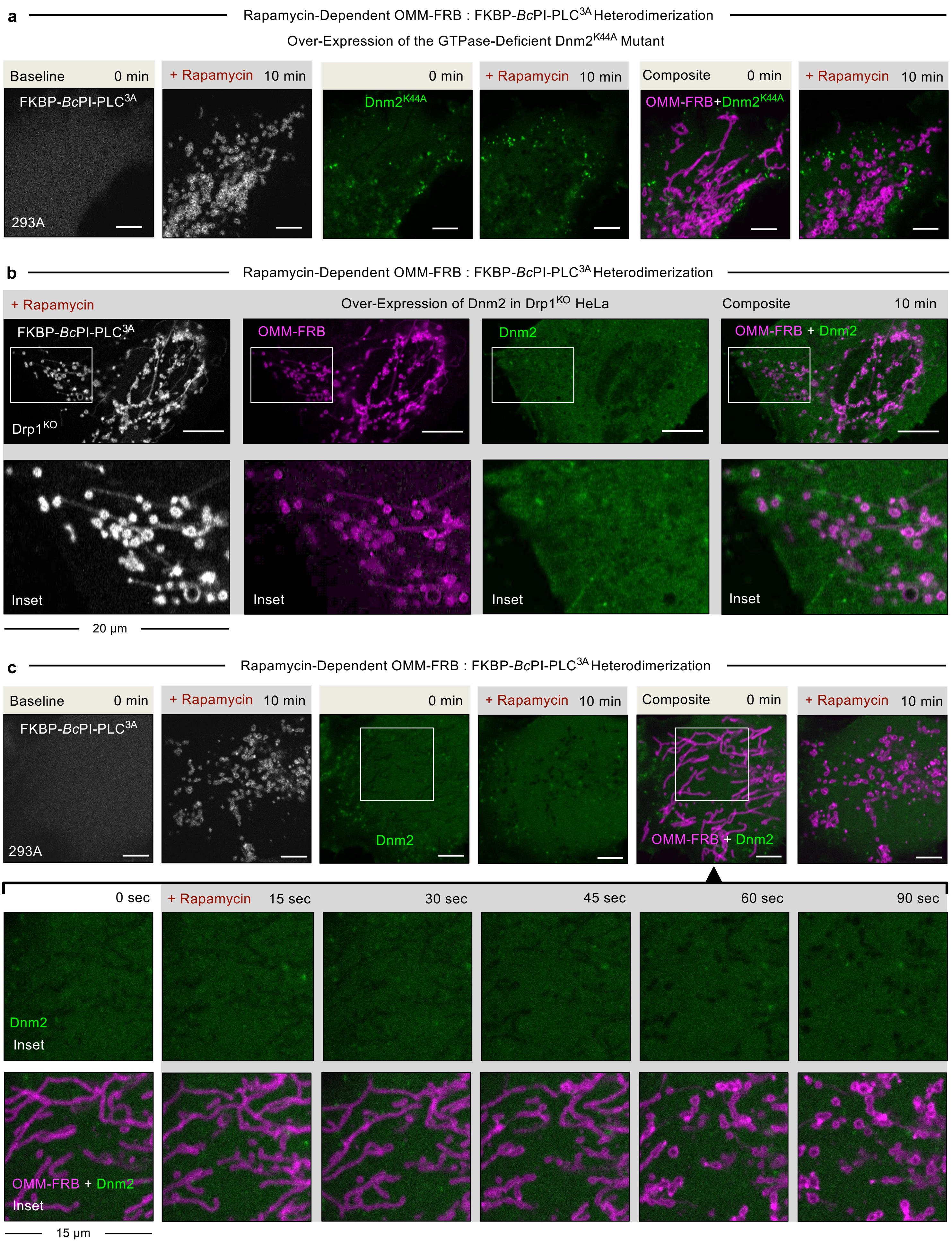
a
Rapamycin-Dependent OMM-FRB : FKBP-BcPI-PLC3A Heterodimerization
Over-Expression of the GTPase-Deficient Dnm2K44A Mutant
Baseline 0 min
+ Rapamycin 10 min
0 min
+ Rapamycin 10 min
Composite 0 min
+ Rapamycin 10 min
FKBP-BcPI-PLC3A
293A
Dnm2K44A
OMM-FRB+Dnm2K44A
b
Rapamycin-Dependent OMM-FRB : FKBP-BcPI-PLC3A Heterodimerization
+ Rapamycin
Over-Expression of Dnm2 in Drp1KO HeLa
Composite 10 min
FKBP-BcPI-PLC3A
Drp1KO
OMM-FRB
Dnm2
OMM-FRB + Dnm2
Inset
Inset
Inset
Inset
20 µm
c
Rapamycin-Dependent OMM-FRB : FKBP-BcPI-PLC3A Heterodimerization
Baseline 0 min
+ Rapamycin 10 min
0 min
+ Rapamycin 10 min
Composite 0 min
+ Rapamycin 10 min
FKBP-BcPI-PLC3A
293A
Dnm2
OMM-FRB + Dnm2
0 sec
+ Rapamycin 15 sec
30 sec
45 sec
60 sec
90 sec
Dnm2
Inset
OMM-FRB + Dnm2
Inset
15 µm

Supplementary Figure 7.

***Bc*PI-PLC^{3A}-induced fission of the mitochondrial network does not require Dnm2.**

(a) Representative composite images of HEK293A cells (5 µm scale bar) showing the localization of the OMM-targeted FRB recruiter (OMM-FRB-mRFP, magenta) and the GTPase-deficient mutant of Dnm2 (mNG^{HO}-Dnm2^{K44A}, green) before (left) or 10 min after (right) rapamycin-induced (100 nM) recruitment of the catalytically active *Bc*PI-PLC^{3A} (emiRFP670-FKBP-*Bc*PI-PLC^{3A}, grey) to the cytosolic membrane leaflet of the mitochondria. Composite images present an overlay of the OMM-FRB together with the Dnm2^{K44A} mutant. **(b)** Representative images of Drp1^{KO} HeLa cells (10 µm scale bar) showing localization of the OMM-targeted FRB recruiter (OMM-FRB-ECFP, magenta) and Dnm2 (Dnm2-mEGFP, green) 10 min after rapamycin-induced (100 nM) recruitment of the catalytically active *Bc*PI-PLC^{3A} (mRFP-FKBP-*Bc*PI-PLC^{3A}, grey) to the cytosolic membrane leaflet of the mitochondria. Composite images present an overlay of the OMM-FRB together with Dnm2. **(c)** Representative images of HEK293A cells (5 µm scale bar) showing the localization of the OMM-targeted FRB recruiter (OMM-FRB-mRFP, magenta) and Dnm2 (mNG^{HO}-Dnm2, green) before (left) or 10 min after (right) rapamycin-induced (100 nM) recruitment of the catalytically active *Bc*PI-PLC^{3A} (emiRFP^{670}-FKBP-*Bc*PI-PLC^{3A}, grey) to the cytosolic membrane leaflet of the mitochondria. The bulk fragmentation response of the mitochondrial network is shown over the 10 min period immediately after rapamycin treatment (top row panels), while a more detailed time series showing the initial 90 sec period post-rapamycin treatment, which is the time frame consistently associated with the majority of the OMM fission events, are presented separately (bottom row inset panels). Composite images present an overlay of the OMM-FRB with Dnm2.

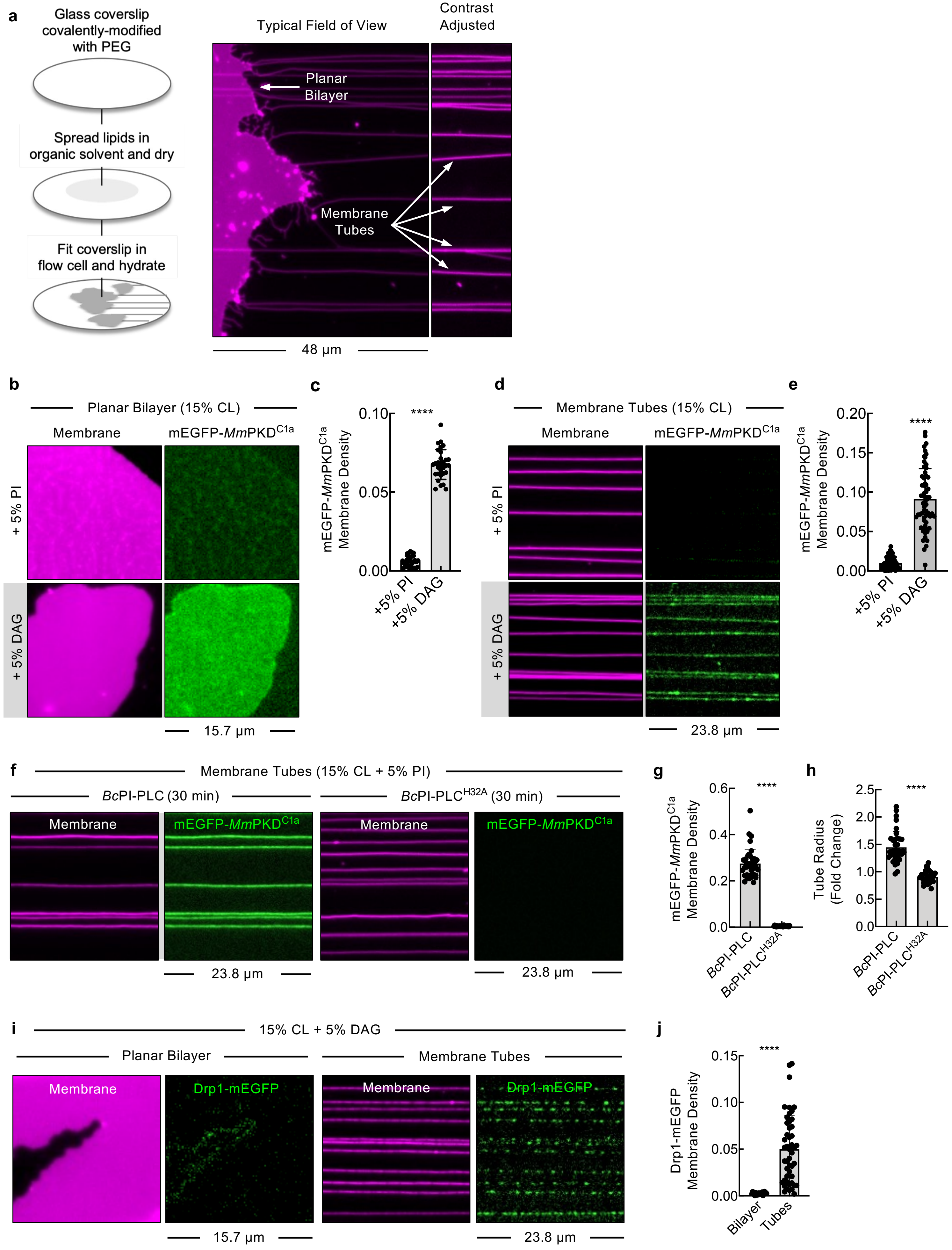
a
Glass coverslip covalently-modified with PEG
Spread lipids in organic solvent and dry
Fit coverslip in flow cell and hydrate
Typical Field of View
Contrast Adjusted
Planar Bilayer
Membrane Tubes
48 µm
b
Planar Bilayer (15% CL)
Membrane
mEGFP-MmPKDC1a
+ 5% PI
+ 5% DAG
15.7 µm
c
mEGFP-MmPKDC1a Membrane Density
0.10
0.05
0.00
****
+5% PI
+5% DAG
d
Membrane Tubes (15% CL)
Membrane
mEGFP-MmPKDC1a
+ 5% PI
+ 5% DAG
23.8 µm
e
mEGFP-MmPKDC1a Membrane Density
0.20
0.15
0.10
0.05
0.00
****
+5% PI
+5% DAG
f
Membrane Tubes (15% CL + 5% PI)
BcPI-PLC (30 min)
BcPI-PLCH32A (30 min)
Membrane
mEGFP-MmPKDC1a
23.8 µm
g
mEGFP-MmPKDC1a Membrane Density
0.6
0.4
0.2
0.0
****
BcPI-PLC
BcPI-PLCH32A
h
Tube Radius (Fold Change)
2.5
2.0
1.5
1.0
0.5
0.0
****
BcPI-PLC
BcPI-PLCH32A
i
15% CL + 5% DAG
Planar Bilayer
Membrane Tubes
Membrane
Drp1-mEGFP
15.7 µm
23.8 µm
j
Drp1-mEGFP Membrane Density
0.15
0.10
0.05
0.00
****
Bilayer
Tubes

Supplementary Figure 8.

Preparation and stability of supported membrane templates (SMrTs).

(a) Schematic of the steps leading to the formation of supported membrane templates (SMrTs) and a representative field of view showing planar bilayers and curved membrane tubes. Modified with permission from Swaminathan and Pucadyil, 2024[178]. **(b)** Representative images of the planar bilayer (magenta) of the indicated lipid composition incubated with the low-affinity DAG sensor mEGFP-*Mm*PKD^{C1a} (*Mus musculus* (*Mm*) protein kinase D (PKD) C1a domain; Residues 276-326; green). **(c)** mEGFP-MmPKD^{C1a} membrane density reported as the ratio of EGFP and membrane fluorescence. Data represents the mean ± SD of at least 30 regions on bilayers for each condition. Statistical significance was estimated using unpaired Mann-Whitney's test. **** denotes $p<0.0001$. **(d)** Representative images of membrane tubes (magenta) of the indicated lipid composition incubated with mEGFP-MmPKD^{C1a} (green). **(e)** mEGFP-MmPKDC1a membrane density reported as the ratio of the EGFP fluorescence and the membrane fluorescence. Data represents the mean ± SD of at least 59 tubes for each condition. Statistical significance was estimated using unpaired Mann-Whitney's test. **** denotes $p<0.0001$. **(f)** Representative images of membrane tubes (magenta) prepared using the indicated lipid composition and treated with recombinant *Bc*PI-PLC or catalytically inactive *Bc*PI-PLC^{H32A} for 30 mins prior to washing and subsequent incubation with mEGFP-*Mm*PKD^{C1a} (green). **(g)** mEGFP-*Mm*PKD^{C1a} membrane density reported as the ratio of the EGFP fluorescence and the membrane fluorescence after treatment with either the active *Bc*PI-PLC enzyme or the catalytically inactive mutant, *Bc*PI-PLC^{H32A}. Data represents the mean SD of at least 29 tubes for each treatment condition. Statistical significance was estimated using unpaired Mann-Whitney's test. **** denotes $p<0.0001$. **(h)** Plot showing the fold change in tube radii upon treating with *Bc*PI-PLC or *Bc*PI-PLC^{H32A}. Data represents the mean SD of at least 26 tubes for each treatment condition. Statistical significance was estimated using an unpaired Mann-Whitney's test. **** denotes $p<0.0001$. **(i)** Representative images of planar bilayer (magenta) and membrane tubes (magenta) prepared of the indicated lipid composition and incubated with Drp1-mEGFP (green). **(j)** Drp1-mEGFP membrane density reported as the ratio of the EGFP fluorescence and the membrane fluorescence. Data represents the mean SD of 22 regions on planar bilayers and 48 tubes. Statistical significance was estimated using an unpaired Mann-Whitney's test. **** denotes $p<0.0001$.

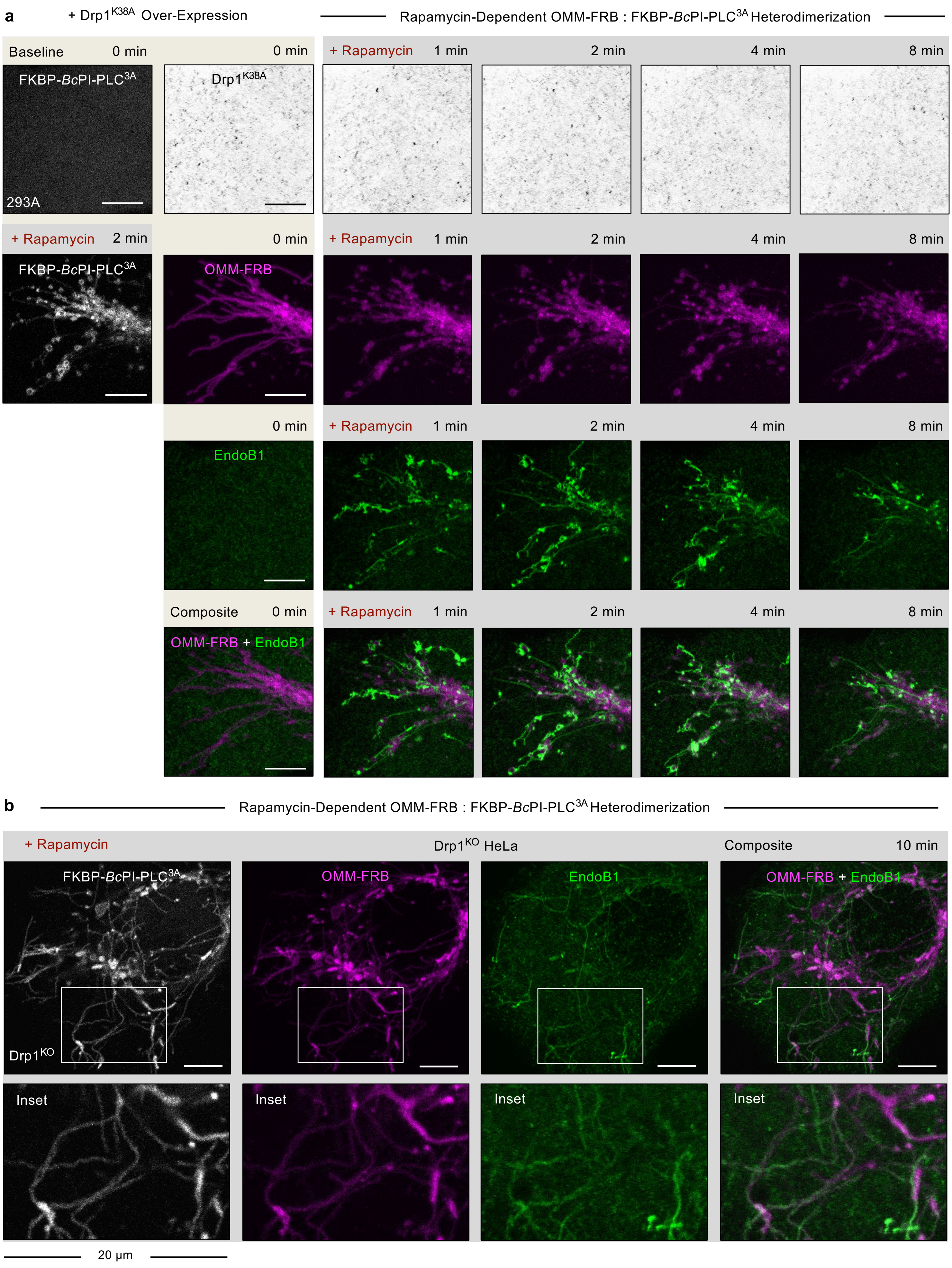

a
+ Drp1K38A Over-Expression
Rapamycin-Dependent OMM-FRB : FKBP-BcPI-PLC3A Heterodimerization
Baseline
0 min
+ Rapamycin
1 min
2 min
4 min
8 min
FKBP-BcPI-PLC3A
Drp1K38A
293A
OMM-FRB
EndoB1
Composite
OMM-FRB + EndoB1
b
Rapamycin-Dependent OMM-FRB : FKBP-BcPI-PLC3A Heterodimerization
Drp1KO HeLa
10 min
Drp1KO
Inset
20 μm

Supplementary Figure 9.

Inhibition of Drp1-dependent fission enhances *Bc*PI-PLC^{3A}-induced translocation of EndoB1 to the OMM and causes formation of mitochondrial tubulations.

(a) Representative images of HEK293A cells (10 µm scale bar) showing the localization of the OMM-targeted FRB recruiter (OMM-FRB-mRFP, magenta), endophilin B1 (EndoB1-mEGFP, green), and the GTPase-deficient mutant of Drp1 (mCherry-Drp1^{K38A}; inverted grey) in response to rapamycin-induced (100 nM) recruitment of the catalytically active FKBP-*Bc*PI-PLC^{3A} (emiRFP^{670}-FKBP-*Bc*PI-PLC^{3A}, grey) to the cytosolic membrane leaflet of the mitochondria. The composite images shown present an overlay of the OMM-FRB together with EndoB1. **(b)** Representative images of Drp1^{KO} HeLa cells (10 µm scale bar) showing the localization of the OMM-targeted FRB recruiter (OMM-FRB-mRFP, magenta) and EndoB1 (EndoB1-mEGFP, green) after 10 min of rapamycin-induced (100 nM) recruitment of the catalytically active FKBP-*Bc*PI-PLC^{3A} (emiRFP^{670}-FKBP-*Bc*PI-PLC^{3A}, grey) to the cytosolic membrane leaflet of the mitochondria. The composite images shown present an overlay of the OMM-FRB together with EndoB1.

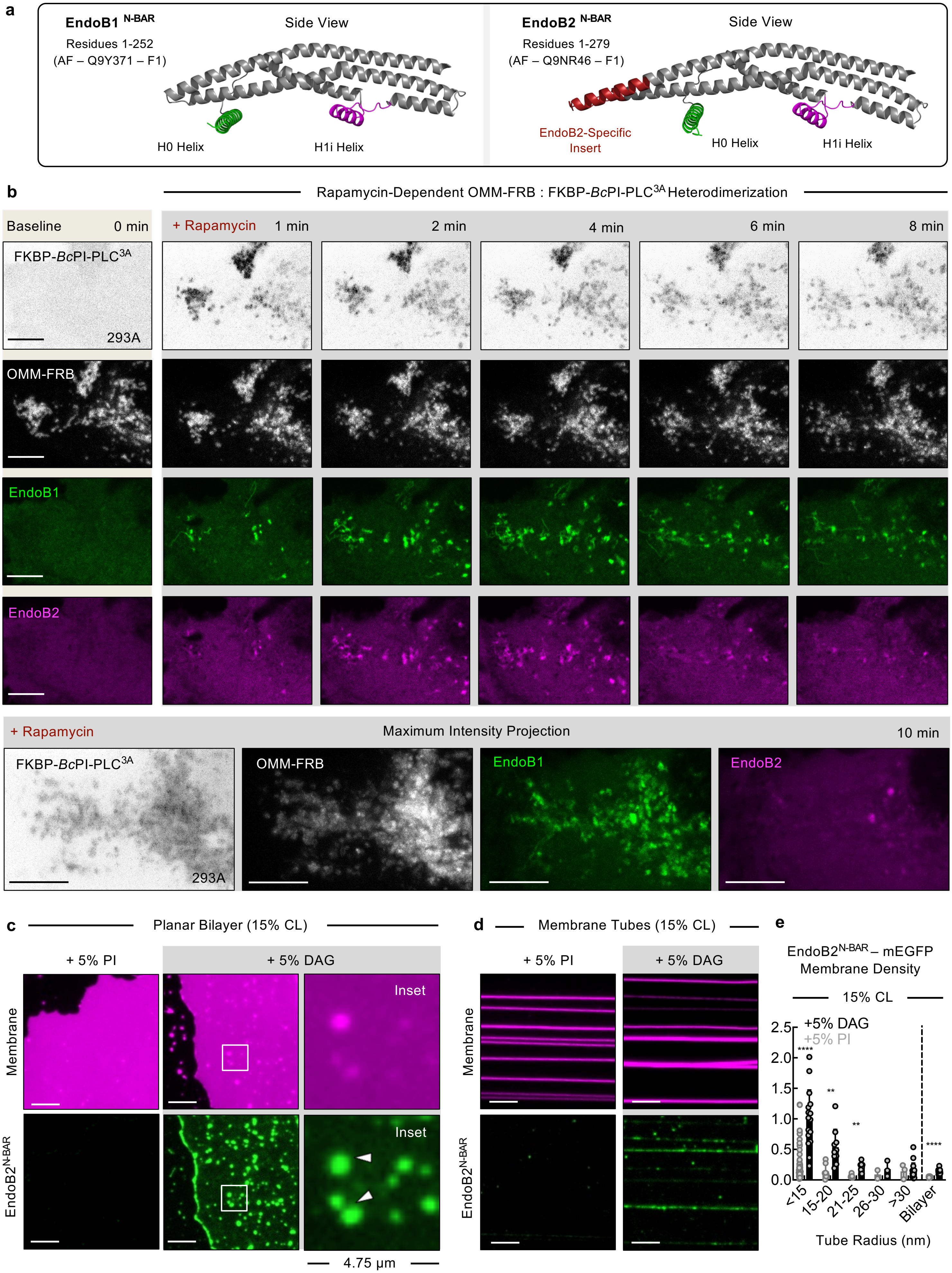
a
EndoB1 N-BAR
Residues 1-252
(AF – Q9Y371 – F1)
Side View
H0 Helix
H1i Helix
EndoB2 N-BAR
Residues 1-279
(AF – Q9NR46 – F1)
Side View
EndoB2-Specific Insert
H0 Helix
H1i Helix
b
Rapamycin-Dependent OMM-FRB : FKBP-BcPI-PLC3A Heterodimerization
Baseline 0 min
+ Rapamycin 1 min
2 min
4 min
6 min
8 min
FKBP-BcPI-PLC3A
293A
OMM-FRB
EndoB1
EndoB2
+ Rapamycin
Maximum Intensity Projection
10 min
FKBP-BcPI-PLC3A
293A
OMM-FRB
EndoB1
EndoB2
c
Planar Bilayer (15% CL)
+ 5% PI
+ 5% DAG
Inset
Membrane
EndoB2N-BAR
Inset
4.75 µm
d
Membrane Tubes (15% CL)
+ 5% PI
+ 5% DAG
Membrane
EndoB2N-BAR
e
EndoB2N-BAR – mEGFP
Membrane Density
15% CL
+5% DAG
+5% PI
2.5
2.0
1.5
1.0
0.5
0.0
****
**
**
****
<15
15-20
21-25
26-30
>30
Bilayer
Tube Radius (nm)

Supplementary Figure 10.

EndoB2 is sensitive to membrane DAG content and translocates to the OMM in response to local FKBP-*Bc*PI-PLC^{3A} recruitment.

(a) Structural comparison of the isolated N-BAR domains from the EndoB1 (AF-Q9Y371-F1) and EndoB2 (AF-Q9NR46-F1) AlphaFold2 predictions (DeepMind)[173,174], specifically highlighting the position of the H_0 (green) and H_{1i} (magenta) helices as well as the location of the EndoB2-specifc insertion (red) that effectively extends the overall length of the curved helical bundle. Protein structures were prepared using the PyMOL Molecular Graphics System (Version 3.0; Schrödinger, LLC). **(b)** Representative images of HEK293A cells (10 µm scale bar) showing the localization of the OMM-targeted FRB recruiter (OMM-FRB-mRFP, grey) together with endophilin B1 (EndoB1-mEGFP, green) and B2 (EndoB2-mScarletI, magenta) in response to rapamycin-induced (100 nM) recruitment of the catalytically active *Bc*PI-PLC^{3A} (mRFP-FKBP-*Bc*PI-PLC^{3A}, inverted grey) to the cytosolic membrane leaflet of the mitochondria. In the bottom-most row, enlarged representative images from this time series are also presented at 15 min post-rapamycin treatment to compare the relative intensities of the EndoB isoforms in OMM-associated tubulations. Note that these images show maximum intensity projections generated from confocal slices along the Z-axis (6.5740 µm total depth). **(c)** Representative images of a planar bilayer (magenta) of the indicated lipid composition after incubation with EndoB2$^{N\text{-}BAR}$-mEGFP (residues, green). White arrows in magnified panels mark protein clusters that coincide with regions of high membrane fluorescence, indicating tubulation. **(d)** Representative images of membrane tubes (magenta) of the indicated lipid composition incubated with EndoB2$^{N\text{-}BAR}$-mEGFP. **(e)** Membrane densities of EndoB2$^{N\text{-}BAR}$-mEGFP are reported as the ratio of EGFP and membrane fluorescence across a range of tube sizes as well as on the planar bilayer of the indicated lipid composition. Data represents the mean ± SD of 54 tubes and 36 different regions on planar bilayers for DAG-containing and 73 tubes and 28 different regions on planar bilayers for PI-containing membranes. Statistical significance was estimated using unpaired Mann-Whitney's test. **** denotes $p<0.0001$, ** denotes $p=0.0027$.

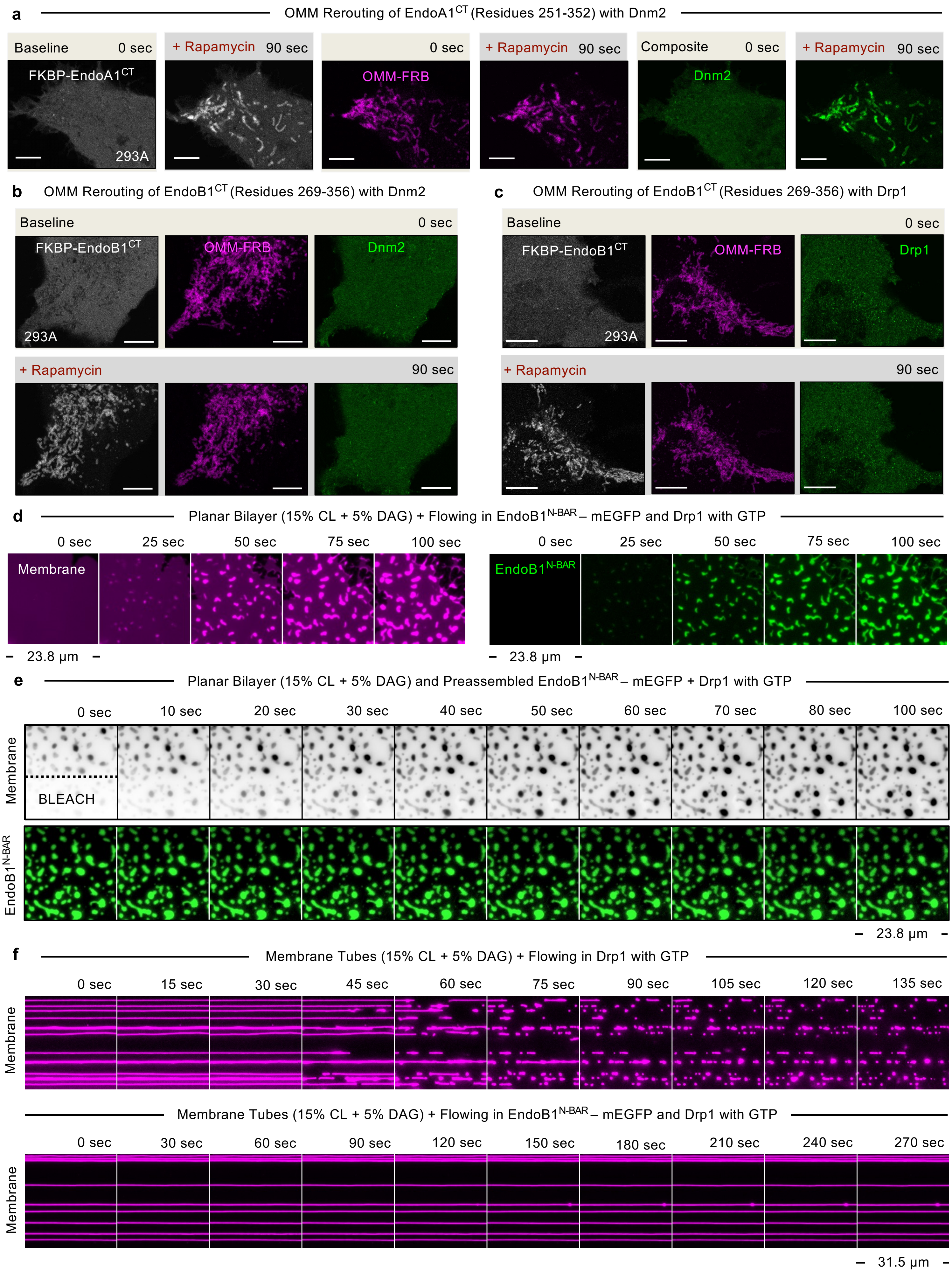
a
OMM Rerouting of EndoA1CT (Residues 251-352) with Dnm2
Baseline
0 sec
+ Rapamycin
90 sec
Composite
FKBP-EndoA1CT
293A
OMM-FRB
Dnm2
b
OMM Rerouting of EndoB1CT (Residues 269-356) with Dnm2
FKBP-EndoB1CT
c
OMM Rerouting of EndoB1CT (Residues 269-356) with Drp1
Drp1
d
Planar Bilayer (15% CL + 5% DAG) + Flowing in EndoB1N-BAR – mEGFP and Drp1 with GTP
25 sec
50 sec
75 sec
100 sec
Membrane
EndoB1N-BAR
23.8 μm
e
Planar Bilayer (15% CL + 5% DAG) and Preassembled EndoB1N-BAR – mEGFP + Drp1 with GTP
10 sec
20 sec
30 sec
40 sec
60 sec
70 sec
80 sec
BLEACH
f
Membrane Tubes (15% CL + 5% DAG) + Flowing in Drp1 with GTP
15 sec
45 sec
105 sec
120 sec
135 sec
Membrane Tubes (15% CL + 5% DAG) + Flowing in EndoB1N-BAR – mEGFP and Drp1 with GTP
150 sec
180 sec
210 sec
240 sec
270 sec
31.5 μm

Supplementary Figure 11.

The extended C-terminus of EndoB1 does not directly bind Drp1 or Dnm2, but membrane assembly of EndoB1 interferes with Drp1-mediated fission.

(a) Representative images of HEK293A cells (10 µm scale bar) showing localization of the OMM-targeted FRB recruiter (OMM-FRB-ECFP, magenta) and Dnm2 (Dnm2-mEGFP, green) before (top row panels) and 90 sec after (middle and bottom row panels) rapamycin-induced (100 nM) recruitment of the extended C-terminus of EndoA1 (mRFP-FKBP-EndoA1^{CT}, Residues 251-352, grey) to the cytosolic membrane leaflet of the mitochondria. **(b, c)** Representative images of HEK293A cells (10 µm scale bar) showing localization of the OMM-targeted FRB recruiter (OMM-FRB-ECFP, magenta) and either Dnm2 (**b**; Dnm2-mEGFP, green) or Drp1 (**c**; mEGFP-Drp1, green) before (top row panels) and 90 sec after (middle and bottom row panels) rapamycin-induced (100 nM) recruitment of the extended C-terminus of EndoB1 (mRFP-FKBP-EndoB1^{CT}, Residues 269-356, grey). **(d)** Representative time-lapse images of the planar bilayer (magenta) of the indicated lipid composition responding to flowing in EndoB1$^{N\text{-}BAR}$-mEGFP (green) mixed with Drp1 and GTP, which are also presented as **Supplementary Movie 9**. **(e)** Representative time-lapse images showing fluorescence recovery after photobleaching the intrinsic fluorescent lipid probe in a large area of the planar bilayer (gray scale, inverted) incubated with EndoB1$^{N\text{-}BAR}$-mEGFP (green) and mixed with Drp1 and GTP, which are also presented as **Supplementary Movie 10**. Region below the dotted line is where the lipid probe was bleached. **(f)** Representative time-lapse images of the membrane tubes (magenta) of the indicated lipid composition while flowing in Drp1 with GTP (top row panels) or Drp1 and GTP mixed with EndoB1$^{N\text{-}BAR}$-mEGFP (green channel not shown; bottom row panels).

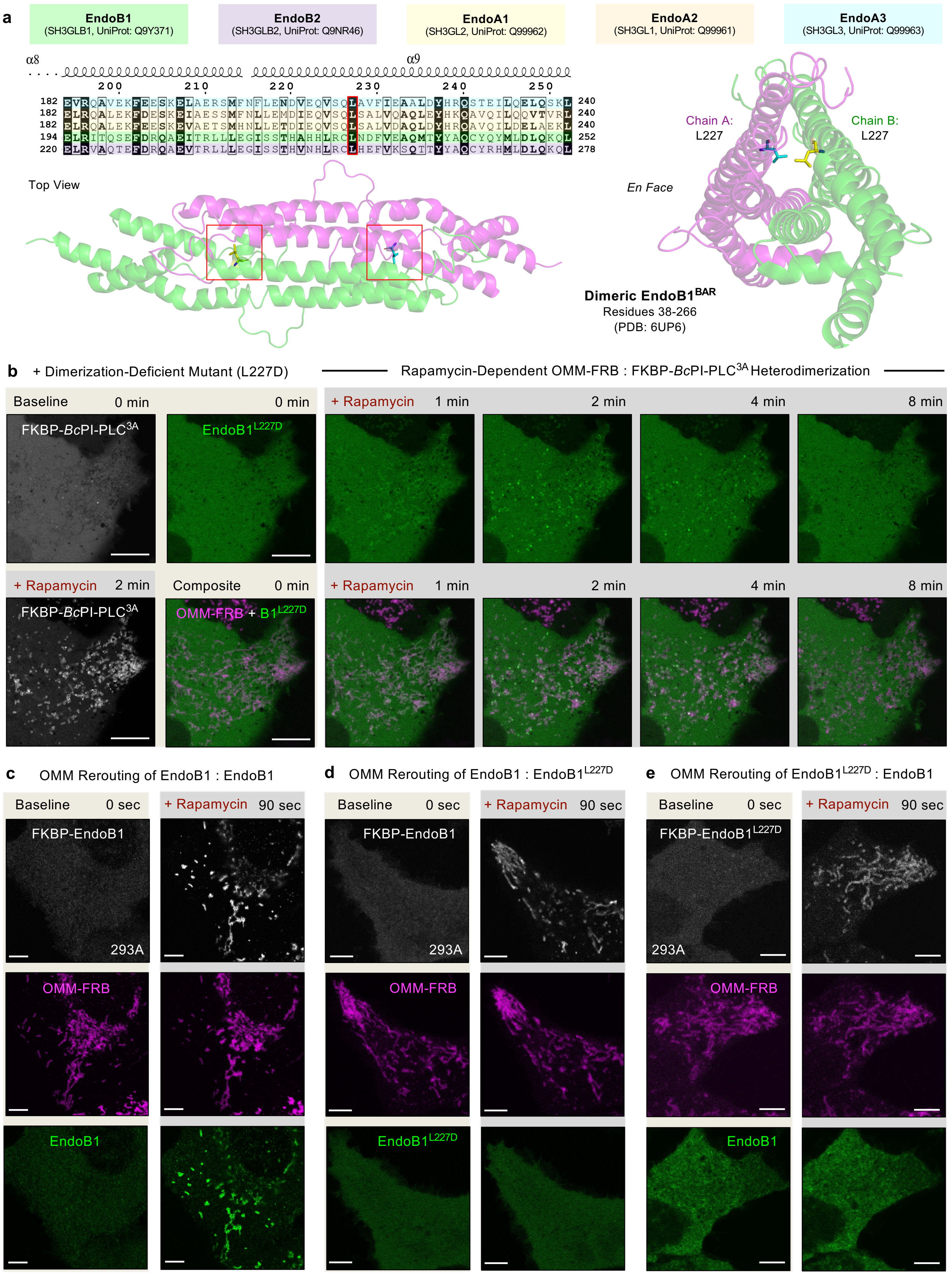
a
EndoB1
(SH3GLB1, UniProt: Q9Y371)
EndoB2
(SH3GLB2, UniProt: Q9NR46)
EndoA1
(SH3GL2, UniProt: Q99962)
EndoA2
(SH3GL1, UniProt: Q99961)
EndoA3
(SH3GL3, UniProt: Q99963)
α8
α9
200
210
220
230
240
250
Chain A:
L227
Chain B:
L227
En Face
Top View
Dimeric EndoB1^BAR
Residues 38-266
(PDB: 6UP6)
b
+ Dimerization-Deficient Mutant (L227D)
Rapamycin-Dependent OMM-FRB : FKBP-BcPI-PLC^3A Heterodimerization
Baseline 0 min
0 min
FKBP-BcPI-PLC^3A
EndoB1^L227D
+ Rapamycin 1 min
2 min
4 min
8 min
+ Rapamycin 2 min
Composite 0 min
OMM-FRB + B1^L227D
c
OMM Rerouting of EndoB1 : EndoB1
Baseline 0 sec
+ Rapamycin 90 sec
FKBP-EndoB1
293A
OMM-FRB
EndoB1
d
OMM Rerouting of EndoB1 : EndoB1^L227D
FKBP-EndoB1
293A
OMM-FRB
EndoB1^L227D
e
OMM Rerouting of EndoB1^L227D : EndoB1
FKBP-EndoB1^L227D
293A
OMM-FRB
EndoB1

Supplementary Figure 12.

Inhibition of homodimerization inhibits the DAG-induced membrane assembly of EndoB1.

(a) Sequence alignment highlighting the conservation of leucine 227 (EndoB1 residue numbering, top left) among the five human endophilin isoforms (ESPript3.0)[177] alongside the dimeric structure of the BAR domain of EndoB1 (Residues 38-266; chain A, magenta; chain B, green), which was extracted from the Cryo-EM map of the EndoB1 homo-oligomer (PDB: 6UP6)[115]. Please note the positioning of the L227 residues, which are highlighted as stick representations, at the core interface between the individual chains of the EndoB1 homodimer. Protein structures were prepared using the PyMOL Molecular Graphics System (Version 3.0; Schrödinger, LLC). **(b)** Representative images of HEK293A cells (10 µm scale bar) showing the localization of the OMM-targeted FRB recruiter (OMM-FRB-mRFP, magenta) and an EndoB1 point-mutant (L227D) with significantly reduced homodimerization ($EndoB1^{L227D}$-mEGFP, green) in response to rapamycin-induced (100 nM) recruitment of the catalytically active *Bc*PI-PLC^{3A} (mRFP-FKBP-*Bc*PI-PLC^{3A}, grey) to the cytosolic membrane leaflet of the mitochondria. Composite images present an overlay of the OMM-FRB together with $EndoB1^{L227D}$. **(c)** Representative images of HEK293A cells (10 µm scale bar) showing localization of the OMM-targeted FRB recruiter (OMM-FRB-ECFP, magenta) and EndoB1 (EndoB1-mEGFP, green) before (left) and 90 sec after (right) rapamycin-induced (100 nM) recruitment of EndoB1 (EndoB1-FKBP-mRFP, grey) to the cytosolic membrane leaflet of the mitochondria. The complete time series associated with the images presented is provided as **Supplementary Movie 11**. **(d)** Representative images of HEK293A cells (10 µm scale bar) showing localization of the OMM-targeted FRB recruiter (OMM-FRB-ECFP, magenta) and the L227D mutant of EndoB1 ($EndoB1^{L227D}$-mEGFP, green) before (left) and 90 sec after (right) rapamycin-induced (100 nM) recruitment of EndoB1 (EndoB1-FKBP-mRFP, grey) to the cytosolic membrane leaflet of the mitochondria. **(e)** Representative images of HEK293A cells (10 µm scale bar) showing localization of the OMM-targeted FRB recruiter (OMM-FRB-ECFP, magenta) and of EndoB1 (EndoB1-mEGFP, green) before (left) and 90 sec after (right) rapamycin-induced (100 nM) recruitment of the $EndoB1^{L227D}$ mutant ($EndoB1^{L227D}$-FKBP-mRFP, grey) to the cytosolic membrane leaflet of the mitochondria.

Supplementary Table 1: Primers used in this study

DNA Constructs for Mammalian Expression

Construct	PCR Template	Primers	Sequence (5' to 3')	Cloning Enzymes	Cloning Backbone
NES-mEGFP-*Mm*PKD^{C1a,b} F157A	NES-mEGFP-*Mm*PKD^{C1a,b}	Forward	CATTCATACAGAGCCCCAGCTGCCTGTGATCACTGTGGAGAAATG	N/A	N/A
		Reverse	CATTTCTCCACAGTGATCACAGGCAGCTGGGGCTCTGTATGAATG		
NES-mEGFP-*Mm*PKD^{C1a,b} W166A	NES-mEGFP-*Mm*PKD^{C1a,b}	Forward	GTGATCACTGTGGAGAAATGCTGGCGGGGCTGGTGCGTCAAGGCC	N/A	N/A
		Reverse	GGCCTTGACGCACCAGCCCCGCCAGCATTTCTCCACAGTGATCAC		
NES-mEGFP-*Mm*PKD^{C1a,b} V289A	NES-mEGFP-*Mm*PKD^{C1a,b}	Forward	GCCCACGGCCTGCCAGTTCTG	N/A	N/A
		Reverse	TGGCAGGCCGTGGGCCGTG		
NES-mEGFP-*Mm*PKD^{C1a,b} F301A	NES-mEGFP-*Mm*PKD^{C1a,b}	Forward	GGCCTCGCCCGGCAGGGCTTGC	N/A	N/A
		Reverse	CTGCCGGGCGAGGCCCTTGAGGAGC		
mNG^{HO}-Drp1 K38A	mNG^{HO}-Drp1	Forward	GCAGCGGAGCGAGCTCCGTGC	N/A	N/A
		Reverse	GGAGCTCGCTCCGCTGCTCTGCG		
mNG^{HO}-Drp1 T59A	mNG^{HO}-Drp1	Forward	GGAATTGTCGCCCGGAGACCTCTCATTCTGC	N/A	N/A
		Reverse	GGTCTCCGGGCGACAATTCCAGTACC		
emiRFP670-FKBP-*Bc*PI-PLC^{3A}	pemiRFP670-N1	Forward	ATATGCTAGCGCTACCGGTCGCCACCATGGCGGAAGGCTCCGTCGC	NheI	mRFP-FKBP-*Bc*PI-PLC^{3A}
		Reverse	ATATAGATCTGCTCTCAAGCGCGGTGATCCG	BglII	
mNG^{HO}-Dnm2	Dnm2-mCherry	Forward	ATATAGATCTACCATGGGCAACCGCG	BglII	pmNG^{HO}-C1
		Reverse	ATATGGTACCTCAGTCGAGCAGGGATGGCTCGG	KpnI	
mNG^{HO}-Dnm2 K44A	mNG^{HO}-Dnm2	Forward	GCCGGCGCGAGCTCGGTGC	N/A	N/A
		Reverse	CGAGCTCGCGCCGGCGCTCTGG		
EndoB1-mEGFP	pcDNA3.1-EndoB1	Forward	ATATGAATTCGCCGCCTAGGATGAATATCATGGACTTCAACGTGAAGAAGCTGG	EcoRI	pEGFP-N1
		Reverse	ATATACCGGTGGACTTCCGCCTCCGCCGCTATTGAGCAGTTCTAAGTAGGTAATTGGCACCTTGC	AgeI	
EndoB1^{N-BAR}-mEGFP	pcDNA3.1-EndoB1	Forward	ATATGAATTCGCCGCCTAGGATGAATATCATGGACTTCAACGTGAAGAAGCTGG	EcoRI	pEGFP-N1
		Reverse	ATATACCGGTGGACTTCCGCCTCCGCCGCTAGGTGTCACAGAAGTCTGATTGTTGTTACTAAGATAATTGG	AgeI	
EndoB2-mEGFP	pOTB7-SH3GLB2 (EndoB2)	Forward	ATATGAATTCCACGCCATGGACTTCAACATGAAGAAGC	EcoRI	pEGFP-N1
		Reverse	ATATACCGGTGGACTTCCGCCTCCGCCGCTGCTGAGCAGTTCCAAGTAGGTGACAGG	AgeI	
EndoB2^{N-BAR}-mEGFP	pOTB7-SH3GLB2	Forward	ATATGAATTCCACGCCATGGACTTCAACATGAAGAAGC	EcoRI	pEGFP-N1
		Reverse	ATATACCGGTGGACTTCCGCCTCCGCCGCTGGGCTCTGTGGTGCCCACGAAGG	AgeI	
EndoB1^{L227D}-mEGFP	EndoB1-mEGFP	Forward	CGCTGTGACAATGACTTTGTAGAAGCCC	N/A	N/A
		Reverse	GTCATTGTCACAGCGAAGGTGATGGG		
mRFP-FKBP-EndoA1^{CT}	pcDNA3.1-SH3GL2 (EndoA1)	Forward	ATATAAGCTTTGCCTAGAAGGGAATATCAACCTAAACCACGAATGAGC	HindIII	mRFP-FKBP-Type IV 5-ptase Domain
		Reverse	ATATGGTACCCTAATGGGGCAGGGCAACCAGAATTTC C	KpnI	
mRFP-FKBP-EndoB1^{CT}	EndoB1-mEGFP	Forward	ATATGAGCTCCTGTACCATCAGTTTTACCAAATGCGATTGG	SacI	mRFP-FKBP-Type IV 5-ptase Domain
		Reverse	ATATGGTACCTCAATTGAGCAGTTCTAAGTAGGTAATTGGCACCTTGC	KpnI	

Supplementary Table 1: Primers used in this study (continued)

DNA Constructs for Mammalian Expression

Construct	PCR Template	Primers	Sequence (5’ to 3’)	Cloning Enzymes	Cloning Backbone
EndoB1-FKBP-mRFP	EndoB1-mEGFP	Forward	ATATGCTAGCGCCGCCTAGGATGAATATCATGGACTTCAACGTGAAGAAGCTGG	NheI	ACDB3-FKBP-mRFP
		Reverse	ATATGAATTCGCATTGAGCAGTTCTAAGTAGGTAATTGGCACCTTGC	EcoRI	
EndoB1^{L227D}-FKBP-mRFP	EndoB1^{L227D}-mEGFP	Forward	ATATGCTAGCGCCGCCTAGGATGAATATCATGGACTTCAACGTGAAGAAGCTGG	NheI	ACDB3-FKBP-mRFP
		Reverse	ATATGAATTCGCATTGAGCAGTTCTAAGTAGGTAATTGGCACCTTGC	EcoRI	
EndoB1-sLuc	AKAP1^{TM}-mVenus-T2A-sLuc-NES-*Mm*PKD^{C1a,b}	Forward	ATATCGATCGGGTGGTAGTGCTGGTGGTAGTGCTGGTGGTAGTGCTGGTGGTCCGGTCGCCACCATGGCTTCC	PvuI	EndoB1-EGFP
		Reverse	ATATTGTACAGCTGCTCGTTCTTCAGCACTCTCTCCACGAAGC	BsrGI	
AKAP1^{TM}-mVenus-T2A-EndoB1-sLuc	EndoB1-sLuc	Forward	ATATACCGGTCATGAATATCATGGACTTCAACGTGAAGAAGCTGG	AgeI	AKAP1^{TM}-mVenus-T2A-sLuc-NES-*Mm*PKD^{C1a,b}
		Reverse	ATATGGTACCTCACTGCTCGTTCTTCAGCACTCTCTCCACG	KpnI	
AKAP1^{TM}-mVenus-tPT2A-EndoB1-sLuc	tPT2A (IDT gBlock™)	Forward	ATATGTCGACGGCAGCGGCGCCACAAACTTCTCTCTGC	SalI	AKAP1^{TM}-mVenus-T2A-EndoB1-sLuc
		Reverse	ATATACCGGTGGGCCGGGATTCTCCTCCACG	AgeI	
AKAP1^{TM}-mVenus-tPT2A-EndoB1$^{N\text{-}BAR}$-sLuc	EndoB1$^{N\text{-}BAR}$-mEGFP	Forward	ATATACCGGTCATGAATATCATGGACTTCAACGTGAAGAAGCTGG	AgeI	AKAP1^{TM}-mVenus-tPT2A-EndoB1-sLuc
		Reverse	ATATCGATCGTCCCGCGGCCGCGCTAGGTGTCACAGAAGTCTGATTGTTGTTACTAAGATAATTGG	NotI	
AKAP1^{TM}-mVenus-tPT2A-EndoB1$^{\Delta H0}$-sLuc	EndoB1$^{\Delta H0}$-mEGFP	Forward	ATATACCGGTCATGAACATCATGGGCCAAGCCGAGAAAACC	AgeI	AKAP1^{TM}-mVenus-tPT2A-EndoB1-sLuc
		Reverse	ATATCGATCGTCCCGCGGCCGCGCTATTGAGCAGTTCTAAGTAGGTAATTGGCACCTTGC	NotI	
AKAP1^{TM}-mVenus-tPT2A-EndoB1$^{\Delta H1i}$-sLuc	EndoB1$^{\Delta H1i}$-mEGFP	Forward	ATATACCGGTCATGAATATCATGGACTTCAACGTGAAGAAGCTGG	AgeI	AKAP1^{TM}-mVenus-tPT2A-EndoB1-sLuc
		Reverse	ATATCGATCGTCCCGCGGCCGCGCTATTGAGCAGTTCTAAGTAGGTAATTGGCACCTTGC	NotI	
AKAP1^{TM}-mVenus-tPT2A-EndoB1$^{\Delta H0, \Delta H1i}$-sLuc	EndoB1$^{\Delta H0, \Delta H1i}$-mEGFP	Forward	ATATACCGGTCATGAACATCATGGGCCAAGCCGAGAAAACC	AgeI	AKAP1^{TM}-mVenus-tPT2A-EndoB1-sLuc
		Reverse	ATATCGATCGTCCCGCGGCCGCGCTATTGAGCAGTTCTAAGTAGGTAATTGGCACCTTGC	NotI	
AKAP1^{TM}-mVenus-tPT2A-EndoB1^{L227D}-sLuc	EndoB1^{L227D}-mEGFP	Forward	ATATACCGGTCATGAATATCATGGACTTCAACGTGAAGAAGCTGG	AgeI	AKAP1^{TM}-mVenus-tPT2A-EndoB1-sLuc
		Reverse	ATATCGATCGTCCCGCGGCCGCGCTATTGAGCAGTTCTAAGTAGGTAATTGGCACCTTGC	NotI	

Supplementary Table 2: Primers used in this study					
DNA Constructs for Bacterial Expression					
Construct	PCR Template	Primers	Sequence (5' to 3')	Cloning Enzymes	Cloning Backbone
pHGT-2-mEGFP-*Mm*PKD^{C1a}	NES-mEGFP-*Mm*PKD^{C1a,b}	Forward	ATATGGATCCATGGTGAGCAAGGGCGAGGAGC	BamHI	pHGT-2-*Bc*PI-PLC
		Reverse	ATATGCGGCCGCTTAAACTCCACTGCAATTGTTGGGGATTTTAAATGC	NotI	
pHGT-2-EndoB1-mEGFP	EndoB1-mEGFP	Forward	ATATGGATCCAATATCATGGACTTCAACGTGAAGAAGCTGG	BamHI	pHGT-2-*Bc*PI-PLC
		Reverse	ATATGCGGCCGCTTACTTGTACAGCTCGTCCATGCCGAGAGTGATCC	NotI	
pHGT-2-EndoB1$^{N\text{-}BAR}$-mEGFP	EndoB1$^{N\text{-}BAR}$-mEGFP	Forward	ATATGGATCCAATATCATGGACTTCAACGTGAAGAAGCTGG	BamHI	pHGT-2-*Bc*PI-PLC
		Reverse	ATATGCGGCCGCTTACTTGTACAGCTCGTCCATGCCGAGAGTGATCC	NotI	
pHGT-2-EndoB2$^{N\text{-}BAR}$-mEGFP	EndoB2$^{N\text{-}BAR}$-mEGFP	Forward	ATATGGATCCGACTTCAACATGAAGAAGCTGGCGTCG	BamHI	pHGT-2-*Bc*PI-PLC
		Reverse	ATATGCGGCCGCTTACTTGTACAGCTCGTCCATGCCGAGAGTGATCC	NotI	
