# Supplementary material for: Acute diacylglycerol production activates critical membrane-shaping proteins leading to mitochondrial tubulation and fission
Source: Nat Commun. 2025 Mar 19;16:2685. doi: 10.1038/s41467-025-57439-9 (PMC11920102; doi:10.1038/s41467-025-57439-9)
Supplement: Supplementary file 3 — Description of Addtional Supplementary Files [file 41467_2025_57439_MOESM3_ESM.pdf]

Description of Additional Supplementary Files

Supplementary Movie 1. Recruitment of FKBP-*Bc*PI-PLC^{3A} to the OMM induces acute DAG production and subsequent fragmentation of the mitochondrial network. Time series associated with the images presented in Fig. 3b and Supplementary Fig.3.

Supplementary Movie 2. Acute DAG production within the OMM and the resulting mitochondrial fission is associated with the disassembly Drp1 puncta. Composite time series showing overlay of the OMM-FRB together with either the NES-*Mm*PKD^{C1a,b} probe (left; see also Supplementary Fig.3) or Drp1 (right; see also Fig.3b).

Supplementary Movie 3. Drp1^{KO} prevents complete OMM fission in response to localized recruitment of FKBP-*Bc*PI-PLC^{3A}. Confocal sections through the Drp1^{KO} HeLa cell volume (3.1571 µm total depth) associated with the images presented in Fig.2b (10 µm scale bar).

Supplementary Movie 4. Recruitment of FKBP-*Bc*PI-PLC^{3A} to the OMM induces Drp1-dependent mitochondrial fission. Time series associated with the inset image panels presented in Fig.3b.

Supplementary Movie 5. DAG facilitates the Drp1-catalyzed membrane fission reaction. (A) Time series showing fission of membrane templates containing 15% CL and 5% PI upon flowing Drp1 with GTP (see also Figs.5a,b). **(B)** Time series showing fission of membrane templates containing 15% CL and 5% DAG upon flowing in Drp1 with GTP (see also Figs.5a,b).

Supplementary Movie 6. Inhibition of Drp1 GTPase activity enhances *Bc*PI-PLC^{3A}-induced translocation of EndoB1 to the OMM and causes formation of hyper-constricted mitochondrial tubulations. Time series associated with the images presented in Fig.6a and Supplementary Fig.9a.

Supplementary Movie 7. *Bc*PI-PLC^{3A}-induced translocation of EndoB1 to the OMM causes formation of hyper-constricted mitochondrial tubulations in Drp1^{KO} cells. Time series from a replicate experiment showing EndoB1 enrichment on OMM constrictions formed after recruitment of FKBP-*Bc*PI-PLC^{3A} in Drp1^{KO} HeLa cells (see also Fig.6b and Supplementary Fig.9b).

Supplementary Movie 8. Recruitment of FKBP-*Bc*PI-PLC^{3A} to the OMM induces translocation of EndoB1 and the formation of transient OMM constrictions and tubulations. Time series associated with the images presented in Fig.6c.

Supplementary Movie 9. Real-time tubulation of planar membrane bilayer islands by EndoB1$^{N\text{-}BAR}$. Time series associated with the images presented in Fig.7f, which shows a planar bilayer composed of 5% DAG and 15% CL responding to flowing EndoB1$^{N\text{-}BAR}$-mEGFP. Images are acquired in the membrane fluorescence channel and are shown in gray scale that has been inverted in contrast for clarity.

Supplementary Movie 10. Bulk membrane lipid diffusion is not restricted by EndoB1$^{N\text{-}BAR}$ self-assembly. Time series associated with the images presented in Fig.7g, which shows fluorescence recovery after photobleaching of the intrinsic fluorescent lipid probe on a planar bilayer displaying EndoB1$^{N\text{-}BAR}$-mEGFP-coated tubules. The left panel shows membrane fluorescence in gray scale that has been inverted in contrast for clarity. Right panel shows EndoB1$^{N\text{-}BAR}$-mEGFP-coated tubules in green. The region above the gray line is where the lipid probe was bleached.

Supplementary Movie 11. EndoB1$^{N\text{-}BAR}$-mediated tubulation in the presence of Drp1 and GTP. Time series associated with the images presented in Supplementary Fig.11d, which shows a planar bilayer responding to flowing EndoB1$^{N\text{-}BAR}$-mEGFP mixed with Drp1 and GTP. The left panel shows membrane fluorescence (magenta) and the right panel shows EndoB1$^{N\text{-}BAR}$-mEGFP (green).

Supplementary Movie 12. Bulk membrane lipid diffusion takes place in tubes generated by EndoB1$^{N\text{-}BAR}$ in the presence of Drp1 and GTP. Time series associated with the images presented in Supplementary Fig.11e, which shows fluorescence recovery after photobleaching the intrinsic fluorescent lipid probe on a planar bilayer incubated with EndoB1$^{N\text{-}BAR}$-mEGFP as well as Drp1 and GTP. The left panel shows membrane fluorescence in gray scale that has been inverted in contrast for clarity. Right panel shows EndoB1$^{N\text{-}BAR}$-mEGFP-coated tubules in green. The region below the gray line is where the lipid probe was bleached.

Supplementary Movie 13. OMM rerouting of EndoB1 causes rapid remodeling of the mitochondrial network, but does not induce membrane tubulations. Time series associated with the images presented in Supplementary Fig.12c.
